# Identification of Novel Gymnodimines and Spirolides from the Marine Dinoflagellate *Alexandrium ostenfeldii*

**DOI:** 10.3390/md16110446

**Published:** 2018-11-14

**Authors:** Christian Zurhelle, Joyce Nieva, Urban Tillmann, Tilmann Harder, Bernd Krock, Jan Tebben

**Affiliations:** 1 Marine Chemistry, University of Bremen, Leobener Straße 6, 28359 Bremen, Germany; zurhelle@uni-bremen.de (C.Z.); tharder@uni-bremen.de (T.H.); 2 Alfred Wegener Institute, Helmholtz Centre for Polar and Marine Research, Section Ecological Chemistry, Am Handelshafen 12, 27570 Bremerhaven, Germany; joyce.nieva@awi.de (J.N.); Urban.Tillmann@awi.de (U.T.)

**Keywords:** gymnodimine, spirolide, structure elucidation, neuro-toxin, *Alexandrium ostenfeldii*, harmful algal boom (HAB)

## Abstract

Cyclic imine toxins are neurotoxic, macrocyclic compounds produced by marine dinoflagellates. Mass spectrometric screenings of extracts from natural plankton assemblages revealed a high chemical diversity among this toxin class, yet only few toxins are structurally known. Here we report the structural characterization of four novel cyclic-imine toxins (two gymnodimines (GYMs) and two spirolides (SPXs)) from cultures of *Alexandrium ostenfeldii*. A GYM with *m*/*z* 510 (**1**) was identified as 16-desmethylGYM D. A GYM with *m*/*z* 526 was identified as the hydroxylated degradation product of (**1**) with an exocyclic methylene at C-17 and an allylic hydroxyl group at C-18. This compound was named GYM E (**2**). We further identified a SPX with *m*/*z* 694 as 20-hydroxy-13,19-didesmethylSPX C (**10**) and a SPX with *m*/*z* 696 as 20-hydroxy-13,19-didesmethylSPX D (**11**). This is the first report of GYMs without a methyl group at ring D and SPXs with hydroxyl groups at position C-20. These compounds can be conceived as derivatives of the same nascent polyketide chain, supporting the hypothesis that GYMs and SPXs are produced through common biosynthetic genes. Both novel GYMs **1** and **2** were detected in significant amounts in extracts from natural plankton assemblages (**1**: 447 pg; **2**: 1250 pg; **11**: 40 pg per mL filtered seawater respectively).

## 1. Introduction

Cyclic imines are fast acting phycotoxins associated with harmful marine algal blooms and shellfish toxicity. Their chemical structures have a macrocycle of 14 to 27 atoms in common and two conserved features that include the cyclic imine group and spiroketal ring system. These toxins have been detected in extracts from plankton net tows, in vitro dinoflagellate cultures, and in shellfish tissue. Cyclic imine toxins are further divided into gymnodimines, pinnatoxins/pteriatoxins, portimine, prorocentrolides, spirolides, and spiro-prorocentrimine; for reviews see [1,2]. Currently, 36 of these toxins have been structurally elucidated; however, a much greater structural diversity has been inferred from mass spectrometric fragmentation data of microalgal and shellfish extracts [3,4]. 

**Figure 1:**
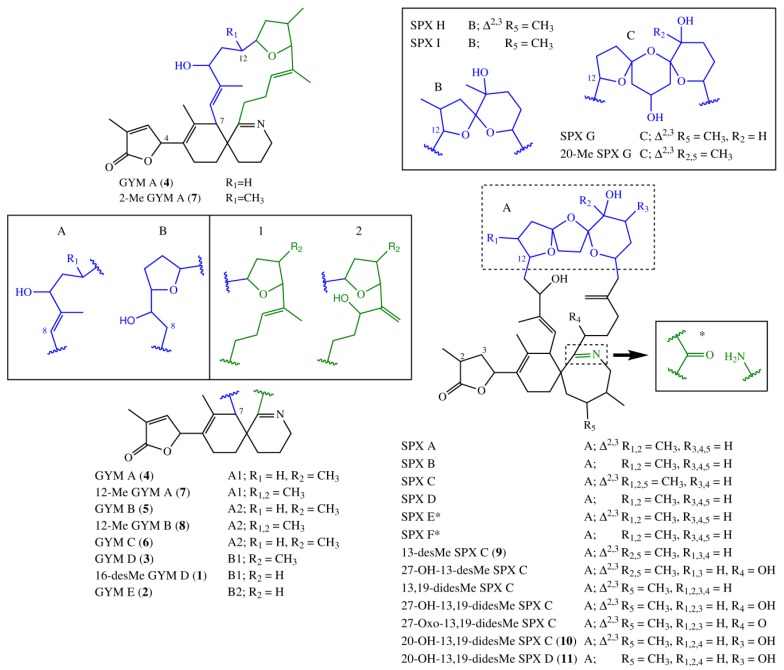
Structural variants of spirolides and gymnodimines. In case of SPX E and SPX F, the imine group is replaced by the structure fragment marked with an asterisk. SPX, spirolide derivative.

Fifteen spirolide derivatives (herein referred to as SPXs) and six gymnodimine derivatives (GYMs) are structurally characterized (Figure 1). Structurally, SPXs and GYMs are highly similar. The dinoflagellate *Karenia selliformis* produces GYMs [1] while some strains of *Alexandrium ostenfeldii* produce both GMYs and SPXs [5,6]. Van Wagoner et al. [7] suggested that this structural similarity is due to common biosynthetic genes. SPXs and GYMs are derived from a linear nascent polyketide chain which is formed by incorporation of small acid units such as acetate and glycine [7]. Originating from an unfolded nascent polyketide chain (NPC), ring B is formed by a Diels Alder reaction, ring A by ester formation, and ring C by nucleophilic attack of a terminal amine group on a carbonyl carbon (C-21 in GYM A), resulting in an imine group [8]. An ether formation via epoxidation is suggested as mechanism to form ring D to F in 13-desmethyl SPX C or ring D and E in GYM D [7,8]. 

Cyclic imines are classified as “fast-acting”, because they induce rapid onset of neurological symptoms followed by death within minutes in mouse bioassays [9]. Both GYMs and SPXs bind to acetylcholine receptors [10,11]. The effect of 13-desmethyl SPX C was diminished after protection of the primary binding site of muscarinic acetylcholine receptors with high concentrations of atropine, suggesting an interaction of the spirolide with the orthologous binding site of the muscarinic acetylcholine receptor [11]. Competition-binding assays confirmed that GYM A reversibly inhibits broad range of nicotinic acetylcholine receptors [10]. 

Martens et al. [6] postulated the presence of various unknown SPXs and two unknown GYMs in addition to GYM A (originally characterized by Seki et al. [12]) and SPX 1 [13] from LC-MS analysis of extracts obtained from cultures of *A. ostenfeldii*. The overarching objective of this work was to purify and structurally characterize these novel cyclic imines and to investigate if all structural derivatives of SPXs and GYMs can be linked back to the same nascent polyketide chain.

## 2. Results and Discussion

### 2.1. Structure Elucidation of 16-Desmethylgymnodimine D (***1***)

**Figure 2:**
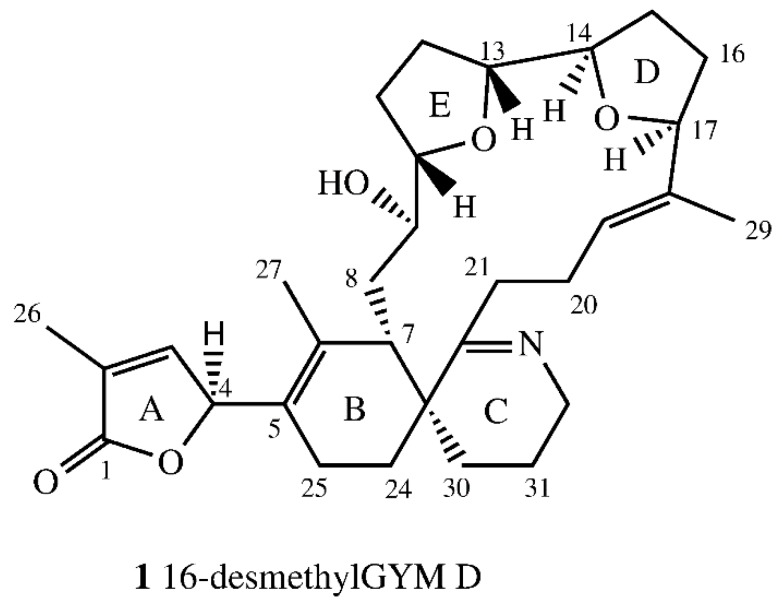
Structure of 16-desmethylgymnodimine D (**1**, numeration as per gymnodimine D).

The empirical formula of compound **1** was determined as C_31_H_43_NO_5_ by high resolution mass spectrometry (HR-ESI-MS) experiments. HR-MS/MS data (Table 1) indicated one less CH_2_ group between C16–21 compared to GYM D. The 600 MHz NMR spectroscopic data of **1** in Pyr-d5 are summarized in Table 2. The NMR-data confirmed a high similarity between the new GYM **1** and GYM D [8]. Larger deviation between carbon shifts of **1** and GYM D was only observed for C-6 and both furan rings (D and E, Figure 2). Due to the overlap of chemical shifts of C-6 and the pyridine signal, a direct determination of the ^13^C-chemical signal was not possible. The carbon shift for C-6 was estimated from HMBC data. The highest deviation in chemical shifts in comparison to GYM-D was observed at C-15 and C-16. No methyl group was observed at C-16. This was confirmed by a DEPT experiment, which showed a negative signal for C-21, characteristic for a CH_2_-group. Initial multiplicity-edited HSQC measurements showed a positive signal for C-21, suggesting a methine or methyl group at that position. We suspect that a partial proton transfer from C-21 to the nitrogen atom caused the positive signal in that experiment. 

The relative stereochemistry of **1** was determined by NOESY and ROESY experiments. C-19 and C-20 showed *E* conformation based on the nuclear Overhauser effect (NOE) between H-20_ab_ and H-29. Further NOE signals were observed between H-9 and H-21. Both showed a NOE with H-7 and H-19 suggesting these protons were directed to the center of macro cyclic ring. The NOE between H-7 and H-21 suggested a small dihedral angle between H7, C-7, C-23 and C-22. Therefore, H-10 and H13 (ring E), as well as H-14 and H-17 (ring D), were on the same side of the tetrahydrofuran rings. H-10 showed a spatial proximity to H-27, suggesting an outward direction of H-10, H-13, H-14, and H-17 from the macro cyclic ring. The complete assignment of centers of chirality at ring E was not possible due to the missing methyl-group at C-16 in comparison to GYM D and the accompanying distinction of groups bound to C-16. No coupling was observed between H-9 and H-10, suggesting a dihedral angle between C-9 and H-9, as well as C-10 and H-10 of circa 90°. To determine stereochemistry at C-4, the circular dichroism (CD) spectrum of **1** was compared to the CD spectrum of GYM A and compared with simulated CD spectra for both (B3LYP optimized) stereoisomers. The experimental CD spectra and simulated CD spectra (Figure S23) of **1** suggest an *S* configuration at C-4, the same as for GYM A (**4**). Based on all available data, the proposed structure of 16-desmethyl GYM D is shown in Figure 2.

### 2.2. Structure Elucidation of Gymnodimine E (***2***)

The structure of GYM E was determined by HRMS/MS spectra, NMR spectra (^1^H-NMR, COSY, HSQC, HSQC-TOCSY) and comparison of NMR data of **2** with GYM B (**5**), GYM D (**3**), and 16-desmethylGYM D (**1**). The empirical formula of **2** was determined as C_31_H_43_NO_6_ by HR-ESIMS. The HRMS/MS spectra of **2** and **1** were almost identical with an up-shift of 15.9950 Da for the fragments larger than *m*/*z* 300, suggesting one additional oxygen (Table 1). HR-MS/MS spectra of **2** also showed a downshift of 2 Da of the fragments above *m*/*z* 258 in comparison with **1** (Table 1) suggesting an additional double bond associated with ring D, introduced by elimination of an additional hydroxyl group in the parent ion. These data suggested that **2** had a similar structure as **1** with an additional hydroxyl group located either at the sidechain between ring C and ring D, or at ring D. 

**Figure 3:**
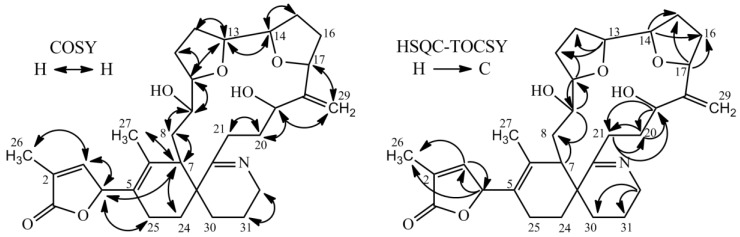
Selected COSY and HSQC-TOCSY correlations in GYM E.

Proton and carbon chemical shifts of **2** revealed a high similarity to **1**, with the exception for C-19, C-20 and C-29 (Table 3). The signal at C-19 suggested a hydroxyl group in comparison to a double bond for **1** at that position. Additionally, **2** showed a characteristic signal for an exocyclic double bond at C-29. Chemical shifts for sidechain between ring C and ring D (C-19 to C-21 and C-29) exhibited closer similarity of chemical shifts to GYM B and GYM C than to **1** (see Table 3, [14,15]). The spin systems for **2** as derived from COSY and HSQC-TOCSY spectra are shown in Figure 3. HSQC-TOCSY only showed correlations without overlap in proton dimension. Therefore, the spin system of ring A and the macrocyclic ring were unambiguous, whereas a lower number of correlations were observed for ring B and C. Ring C was assigned by correlations with C-32. COSY-correlations with C-4 and C-7 led to determinations of C-24 and C-25. The sample amount was in sufficient to assign the stereochemistry; hence, we provide the planar structure of **2** as per Figure 3. The planar structure of **2** is similar to that of 16-desmethylGYM D but contains an exocyclic methylene at C-17 and an allylic hydroxyl group at C-18. 

### 2.3. Structure Elucidation of 20-Hydroxy-13,19-didesmethyl-SPX C (***10***)

The empirical formula of compound **10** was determined as C_41_H_60_O_8_N by HR-ESIMS. Based on CID spectra, Martens et al. (2017) previously proposed a structure similar to 11,23-dihydroxy-19-dehydroxy-13-desmethyl-SPX C for this compound. The CID spectra showed a downshift of 16 Da in the A-type fragment cluster (*m*/*z* 444 to *m*/*z* 428), indicating two hydroxyl-groups between C-1 to C-11 and one between C-22 and C-23. 

**Figure 4:**
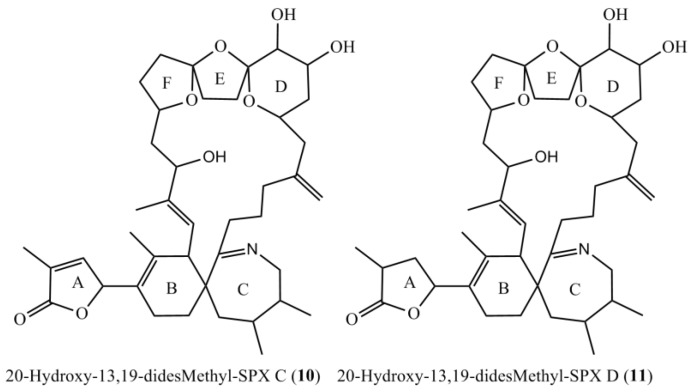
Planar structures of the two novel spirolides 20-Hydroxy-13,19-didesmethyl-SPX C (**10**) and 20-Hydroxy-13,19-didesmethyl-SPX D (**11**).

**Figure 5:**
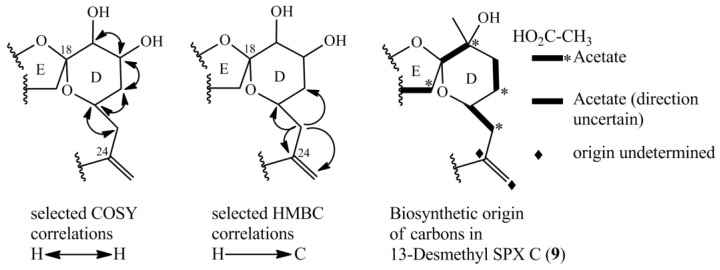
Selected COSY and HMBC correlations in the D-ring system for determining the position of hydroxyl-groups and part of biosynthetic origin of carbons of 13-desmethylSPX C (**9**) [16].

**Figure 6:**
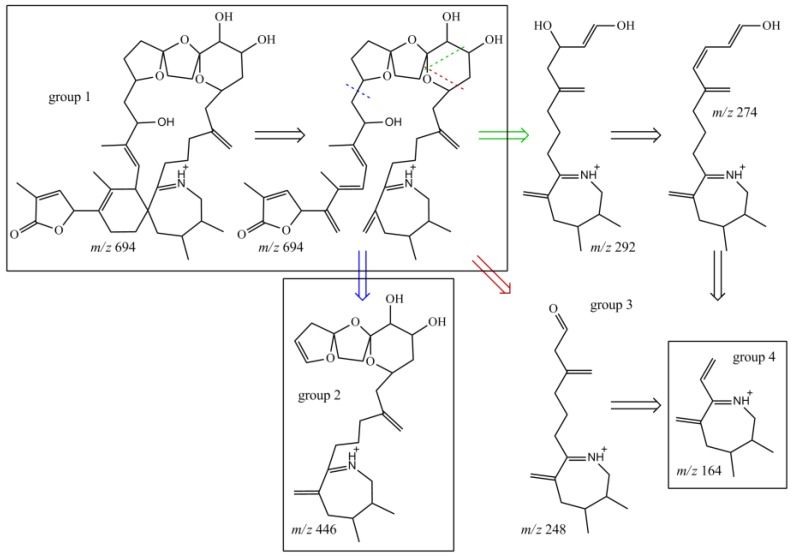
Structures of characteristic fragments in CID-spectra of compound **10**; fragmentation sites marked with dashed line; resulting structure is indicated by arrow in the same color.

The NMR experiments revealed no methyl group at C-19, but instead an additional hydroxyl-group at C-20 (Table 4, Figure 4). The COSY and HMBC experiments clearly revealed correlations from H-23 to C-21 (HMBC) and from H-20 to H-19 and H-21 (COSY), supporting a six-membered ether diol (ring D) structure element (Figure 5). The signals corresponding to C-27 were not detected in MeOD. This was likely due to imine-enamine tautomerism induced proton exchange at this position and reminiscent of similar observations with GYM A [12]. For this reason, GYMs are generally measured in Pyr-d5. Upon re-analysis of **10** in Pyr-d5, the signal for C-27 was clearly detected (Table 4). The signal intensity for C-28 also improved in Pyr-d5. The NMR data contradicted the earlier structural determination of **10** by CID [6]. Therefore, we reanalyzed **10** by HR-MS/MS (Table 5) and propose a fragmentation pathway as shown in Figure 6. Martens et al. interpreted the fragment at *m*/*z* 446 as a Group 1-type fragment corresponding to the *m*/*z* 444 fragment in **9** (Figure 6) [6]. Instead, we suggest, this fragment is formed by dissociation of the bond between C-11 and C-12 leading to the A-type fragment with two hydroxyl groups. This is supported by the observation of the dehydration of the hydroxyl groups resulting in fragments *m*/*z* 428 (C_26_H_40_O_5_N^+^) and *m*/*z* 410 (C_26_H_38_O_4_N^+^). In conclusion, we interpret the A-type fragment upshift in comparison to **9** as 2 Da contrary to the previously proposed downshift by 16 Da [6]. The observed fragment of *m*/*z* 464 (C_26_H_42_O_5_N^+^) as analogous to the *m*/*z* 462 fragment observed for **9**. Therefore, both NMR and HR-MS/MS data supported 20-Hydroxy-13,19-didesMethyl-SPX C as proposed structure of compound **10**. Yields were insufficient to assign the stereochemistry of **10**; therefore, the planar structure of **10** is shown in Figure 4.

### 2.4. Structure Elucidation of 20-Hydroxy-13,19-didesmethyl-SPX D

The empirical formula of compound **11** was determined as C_41_H_62_O_8_N by HR-ESI-MS. CID-spectra showed the same fragmentation pathway as for **10** except for an upshift of 2 Da, suggesting a reduced double bond between C-1 and C-12. NMR data supported this, showing a reduced double bond in the butenolide ring (C-2/3, Table 4) in comparison to **10** and in accordance with the spectra for SPX D [17]. Therefore, we propose the structure of **11** as 20-Hydroxy-13,19-didesmethyl-SPX D as shown in Figure 4. 

### 2.5. Biosynthesis of GYMs and SPXs

SPXs and GYMs share many structural features (Figure 1) [5,8,12,13,14,15,17,18,19,20,21,22,23,24] and are likely biosynthesized by common genes [7]. The compounds elucidated in this study introduce a new degree of variability to the structural diversity of GYMs and SPXs. This is the first report of a GYM without a methyl group at ring D, and that of a SPX with an additional hydroxyl group at C-20. In future studies aiming at the discovery of novel toxins, this new structural diversity should be taken into consideration. 

**Figure 7:**
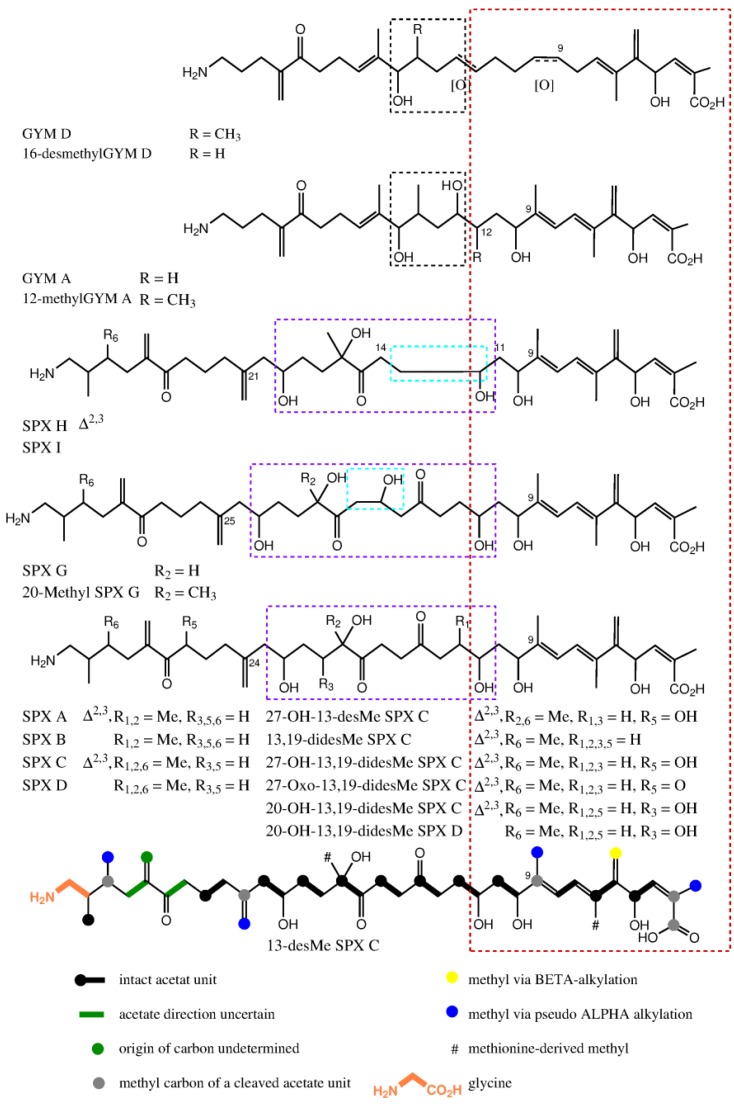
Stacked view of proposed nascent polyketide chains for spirolides and gymnodimines; part with high similarity cornered in red; ring D of GYMs cornered in black; difference between nascent polyketide chains of spirolides are cornered in light blue and origins for rings D, E and F are cornered in violet. The proposed nascent polyketide chain of 13-Desmethyl spirolide C is shown at the bottom with the colored biological origin of nuclei.

Compound **1** and GYM D [8] can be conceived from the same putative nascent polyketide chain (NPC), the only difference being the missing methyl alkylation at C-16. In comparison to GYM A, both of these compounds have one additional carbon between C-7 and C-12 and one less methyl group around C-9 (Figure 7). In the assembled toxin, this difference in NPCs, leads to one additional ring (Figure 1). An additional carbon could indicate a cleaved acetate unit in GYM A, which is a common moiety in dinoflagellate polyketides [7].

The novel spirolides **10** and **11** differ by an unsaturated bond between C-2 and C-3. These SPXs can be conceived from the same NPC with an additional hydroxyl-group at C-20 and one less methyl-group at C-19 in comparison to 13-desmethylSPX C (**9**). In **9**, C-20 and C-21 originate from an intact acetate (Figure 5, C20 from the carboxylic acid, C21 from the methyl group of the acetate) [16]. An incomplete reduction during assembly of NPC would lead to a hydroxylation at C-20 as in **10** and **11** (Figure 7). Therefore, only small changes in the biosynthetic pathway may explain most structural differences between GYMs and SPXs, supporting the hypothesis that these compounds are synthesized by expression of common genes.

### 2.6. Formation of GYM E

**Figure 8:**
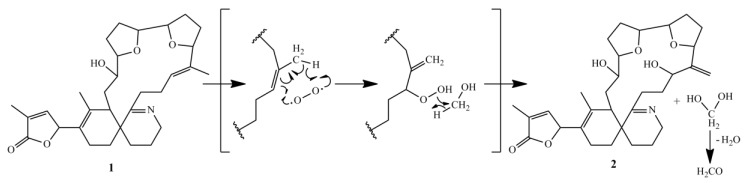
Proposed reaction mechanism of degradation of 16-desmethyl GYM D to GYM E.

An artificial degradation of GYM A to either GYM B or GYM C is generally considered as unlikely, because this would require both isomerization and oxidation [15]. However, we found evidence for an abiotic reaction of 16-desmethyl GYM D to GYM E. The formation of GYM E in a methanolic extract was observed by sequential mass spectrometric quantification of a 16-desmethyl GYM D containing extract, whereby the relative amount of 16-desmethyl GYM D decreased whereas the concentration of GYM E increased (Data not shown). The proposed mechanism of this reaction (Figure 8) combines isomerization and oxidation in a single reaction step. 

### 2.7. Quantification of the Novel Cyclic Imines in Natural Plankton Assemblagess

To confirm the presence of the novel toxins in natural plankton assemblages, extracts of plankton filtered onto glass fiber filters were analyzed by LC-MS/MS with the transitions for **1**, **2**, **10** and **11** (Table 6). The samples were collected in July 2013 during a bloom in Ouwerkerkse Kreek (51°62′ N, 3°99′ E, The Netherlands) from which the cultured strain *A. ostenfeldii* OKNL48 was also first isolated [25]. GYM **1** (447 pg·mL^−1^) was detected in concentrations similar to GYM A (561 pg·mL^−1^) while concentrations of GYM **2** (1250 pg·mL^−1^) even exceeded concentrations of GYM A. 

## 3. Materials and Methods 

### 3.1. Cell Culture and Sample Preparation

*Alexandrium ostenfeldii* was isolated in July 2013 during a bloom in Ouwerkerkse Kreek, The Netherlands and characterized by LC-MS (as strain OKNL48) [6]. Batches of 15 L of OKNL48 were treated with Acetone (7% final concentration) and the toxin content extracted with conditioned HP-20 (25 g per 15 L, 48 h, Diaion Supelco). The resin was collected by filtration and desalted, dried, and stored at −20 °C. The pooled resin (460 g) from a total of 270 L was eluted with methanol, dried under vacuo, and applied to preparative reversed phase chromatography (C18, 25 mm × 310 mm, 5 mL min^−1^) and eluted with a stepwise gradient from water–ACN (80:20) to 100% ACN (30 fractions total). The presence of **1** and **2** was confirmed by LC-MS and the toxin-containing fraction dried, taken up in water–ACN (1:1) (2 mL) and applied to HPLC reversed-phase purification on a C8 column (10 × 150 mm, Machery & Nagel) with solvent A: water and solvent B: acetonitrile (ACN) both containing 0.1% FA. After injection, the samples were eluted isocratically at 15% B for 5 min, followed by a 20 min gradient to 100% B and held for 5 min. The reequilibration phase at 15% B was 5 min. The A final purification step was performed under isocratic elution with water–ACN 45:55 over for 30 min on a Phenyl-Hexyl column (4.6 mm × 150 mm, 1.5 mL·min^−1^, Machery & Nagel). The yields of these fractions were insufficient for structure elucidation of **10** and **11**, therefore, a total of 60 L additional culture was extracted and purified as described above. 

Water samples for the analysis of natural plankton assemblages were taken with a bucket from the surface at three Ouwerkerkse Kreek stations: SL92-1 (51°37′33.7″ N 3°59′23.7″ E), SL92-2 (51°37′45.5″ N 3°59′35.9″ E), and SL92-3 (51°37′44.0″ N 3°59′25.8″ E). The water was filtered onto GFF filters (0.4 μm) and frozen until analysis. Briefly, GFF filters were extracted by reciprocal shaking at maximum speed (6.5 m·s^−1^) for 45 s in a FP 120 FastPrep instrument (Bio101, Thermo Savant, Illkirch, France) containing 0.5 g lysing matrix D (Thermo Savant, Illkirch, France), and 750 µL methanol (Merck, Darmstadt, Germany). After homogenization, the samples were centrifuged (16,000 *g*, 15 min, 4 °C, Centrifuge 5415R, Eppendorf, Hamburg, Germany). Each supernatant was transferred to a spin-filter (pore-size 0.45 mm, Millipore Ultrafree, Eschborn, Germany) and centrifuged for 30 s at 3220 *g*. Filtrates were transferred into HPLC vials (Agilent Technologies, Waldbronn, Germany) for LC-MS/MS analysis. 

### 3.2. Analyses of Cyclic Imines by Chromatography Tandem Mass Spectrometry (LC-MS/MS)

Chromatographic fractions were diluted 1:1000 in 96-well plates and analyzed on a LC-MS/MS system in SRM mode (UPLC: I-Class, MS/MS: Xevo; Waters). The flow-rate was 0.6 mL·min^−1^ isocratic elution with ACN-water (95:5) containing 2.0 mM ammonium formate and 50 mM formic acid resulting in immediate co-elution of all analytes. The runtime was 0.5 min. Cyclic imine containing fractions, filter extracts and the purified toxins were quantified against 13-desMethyl-spirolide C and GYM A (certified reference material; NRC, Halifax, NS, Canada) and expressed as reference standard equivalents (GYM A or SPX1 equivalents) [6] on the same instrumentation as above as follows: A UPLC BEH C18 reverse phase column (Acquity 50 mm × 2.1 mm, 1.7 µm, Waters, Milford, CT, USA) was used with a flow-rate of 0.6 mL·min^−1^ at 40 °C. A gradient elution was performed with two eluants, where eluant A was water and eluent B was acetonitrile/water (95:5 *v*/*v*), both containing 2.0 mM ammonium formate and 50 mM formic acid. Initial conditions were 0.5 min column equilibration with 30% B, followed by a linear gradient to 100% B in 3 min, and isocratic elution for 1 min with 100% B. The system was then returned to initial conditions. The fragments used for the detection of the cyclic imines are given in Table S1.

### 3.3. HR-MS/MS

Accurate mass measurements and fragmentation spectra were acquired with a QExactive Plus mass spectrometer (Thermo Fisher Scientific, Bremen, Germany), using electrospray ionization at a flow-rate of 5 µL per minute. MS measurements were performed in full MS mode with a resolution of 280,000, a scan range of 150 to 2000 *m*/*z* in positive mode using a spray voltage of 3 kV. Capillary temperature was set to 320 °C and the sheath gas was set to 5. Calibration was done against the Calmix standard (Thermo Fisher Scientific).

### 3.4. NMR Analyses

Purified compounds were dried under vacuo and taken up in 45 µL deuterated pyridine (Pyr-d_5_) containing 0.03% TMS (compounds GYM A, **1**, **2** and **10**) or 45 µL deuterated methanol (MeOD; SPX STD, **10** and **11**). NMR experiments were performed in 1.7 mm microtubes at 292 K with a AVANCE II 600 MHz NMR spectrometer (BRUKER) and a CPTCI microcryoprobe. Chemical shift referencing was performed against TMS. BRUKER standard pulse programs were used except for heteronuclear multiple bond correlation (IMPACT-HMBC) [26].

### 3.5. Quantum Chemical Simulation of CD Spectra

Minimal energy geometry was calculated with the GAMESS software package [27,28]. GAMESS was run in parallel on the Linux cluster Cray CS400 “Ollie” at Alfred Wegener Institute’s computing centre, using 36 MPI-tasks on one compute node with two Intel Xeon E5-2697v4 “Broadwell” 18-core CPUs. A semi empirical PM3 level optimization was used prior to density functional theory (DFT) optimization. B3LYP was used in combination with the 6-31G(d) basis set and the “COnductor-like continuum Solvent MOdel” (COSMO) in the “Self-Consistent Reaction Field” method (SCRF). Geometries with minimal energy were used for calculation of rotatory strengths applying time depended DFT with B3LYP, 6-31G(d), and SCRF with COSMO in the ORCA software package [29]. The simulated CD spectrum of **1** was obtained by applying Gaussian broadening to each transition as previously described by Li et al. [30] and adjusted manually to the height of experimental data. 

## 4. Conclusions

Here, we report the structural elucidation of two novel gymnodimines (16-desmethyl GYM D and GYM E) and two novel spirolides (20-Hydroxy-13,19-didesMethyl-SPX C and 20-Hydroxy-13,19-didesMethyl-SPX D) originally detected in cultures of *A. ostenfeldii* [6]. The analysis of a plankton sample obtained during an *A. ostenfeldii* bloom revealed high concentrations of the two novel gymnodimines and one of the spirolides in natural plankton assemblages. 16-desmethyl GYM D and GYM E add new structural variability to the class of GYM toxins. The nascent polyketide chain of GYM D, 16-desmethyl GYM D and GYM E has one more carbon between ring B (C-7) and a furan ring D (C-14) in comparison to all other known SPXs and GYMs. We suggest that this difference in chain length is due to a biosynthetic cleavage of the acetate unit at C-9. This applies to all other cyclic imine toxins whereas this acetate unit is preserved in GYM D-type gymnodimines and spirolides. In comparison, 20-Hydroxy-13,19-didesMethyl-SPX C and 20-Hydroxy-13,19-didesMethyl-SPX D are synthesized from an incomplete reduction of the acetate at C-20. Notably, despite a high structural similarity of SPXs and GYMs, only strains of *A. ostenfeldii* have been confirmed to produce both compound classes. A comparison of the genetic or proteomic differences between *Karenia selliformis* (only GYMs reported) and *A. ostenfeldii* (producing either only SPXs or both toxin groups) may be a promising approach to identify the biosynthetic mechanisms underlying these structural differences. 

## Figures and Tables

**Table 1:** Exact and measured accurate masses (*m*/*z*) for [M + H]^+^ at *m*/*z* 510 and *m*/*z* 526 and their product ions obtained with LC-HRMS.

16-Desmethylgymnodimine D (1)				Gymnodimine E (2)			
Formula	Measured	Calculated	Δ/ppm	Formula	Measured	Calculated	Δ/ppm
C_31_H_44_O_5_N	510.3212	510.3214	−0.33	C_31_H_44_O_6_N	526.3163	526.3163	−0.04
				C_31_H_42_O_5_N	508.3060	508.3057	0.43
C_31_H_42_O_4_N	492.3111	492.3108	−0.54	C_31_H_40_O_4_N	490.2951	490.2952	−0.12
C_30_H_44_O_4_N	482.3264	482.3265	−0.07	C_30_H_42_O_4_N	480.3108	480.3108	−0.14
				C_30_H_42_O_3_N	464.3159	464.3159	−0.12
				C_23_H_32_O_4_N	386.2324	386.2326	−0.46
C_20_H_30_O_3_N	332.2218	332.2220	−0.18	C_20_H_28_O_3_N	330.2063	330.2064	−0.12
C_16_H_24_O_2_N	262.1800	262.1802	−0.16	C_16_H_22_O_2_N	260.1645	260.1645	−0.17
C_17_H_26_ON	260.2007	260.2009	−0.17	C_17_H_24_ON	258.1852	258.18524	−0.12
C_14_H_20_N	202.1589	202.1590	−0.09	C_14_H_20_N	202.1590	202.1590	0.09
C_14_H_18_N	200.1433	200.1434	−0.05	C_14_H_18_N	200.1434	200.1434	0.14
				C_12_H_16_ON	190.1227	190.1226	0.07
C_13_H_18_N	188.1433	188.1434	−0.05	C_13_H_18_N	188.1434	188.1434	0.16
C_13_H_16_N	186.1277	186.1277	−0.02	C_13_H_16_N	186.1278	186.1277	0.25
				C_13_H_14_N	184.1120	184.1120	0.19
C_12_H_16_N	174.1277	174.1277	−0.02	C_12_H_16_N	174.1277	174.1277	0.21
C_12_H_14_N	172.1120	172.1121	−0.05	C_12_H_14_N	172.1121	172.1121	0.09
C_11_H_16_N	162.1276	162.1277	−0.08	C_11_H_16_N	162.1277	162.1277	0.1
C_11_H_14_N	160.1121	160.1121	−0.02	C_11_H_14_N	160.1121	160.1121	0.2
C_11_H_12_N	158.0965	158.0964	0.03	C_11_H_12_N	158.0965	158.0964	0.36

**Table 2:** NMR spectroscopic data of 16-desmethylgymnodimine D (**1**). * Position numbering is analog to gymnodimine D.

Position *	δ (^13^C)/ppm	δ (^1^H)/ppm		COSY	HSQC-TOCSY H -> C	HMBC H -> C
1	175.5					
2	130.2					
3	148.6	7.05		4, 26	4, 26	1, 2, 4, 26
4	81.6	5.93		3, 26	3, 26	2, 3, 5, 6, 25
5	125.9					
6	136	(by HMBC)				
7	43.6	3.16		8, 24	8, 25, 27	5, 6, 8, 9, 22, 23
8	31.8	1.9	1.43	8	7, 11	11, 13
9	71.6	3.66		10, 11	10, 8	7, 8, 10
10	83.4	3.94		9, 15	9, 15, 8	8, 9, 12, 14
11	27	1.79	1.56	10	8, 9, 12	9
12	24.9	1.77		13		13,14,15
13	78.6	4.36		11, 14	11, 14, 16	14, 12
14	82.5	4.13		16	11, 13, 16	12, 13
15	29.4	1.99	1.76		12	9, 14
16	32.1	1.79			15	14, 15
17	82.9	4.15		15	12, 15, 16	18, 19, 28
18	133.1					
19	124.9	5.99		17, 20, 21, 28	20, 21	18, 28, 17, 20, 21
20	21.9	3	2.1	21, 20, 19	19, 21, 29	18, 19, 21, 22
21	31.8	2.76		20	20	19, 20, 22
22	173.3					
23	42.7					
24	33.6	1.59	1.37		7, 8/21, 25, 25, 27	7, 22, 23, 25, 30
25	19.7	1.49	1.94		27, 30, 24, 32	
26	11	1.99		3, 4	3, 4	1, 2, 3
27	18.1	1.92			25, 8, 24, 7	5, 6, 7
29	15.3	1.61			20, 21	17, 18, 19
30	26	1.54	1.44		32, 31	23, 32
31	20.5	1.47			32, 30, 24?	23, 32
32	50.3	3.71	3.48	32, 31	31, 30	22, 30, 31

**Table 3:** Proton and carbon chemical shifts of **1** and **2** in comparison to GYM D [8], GYM B [14] and GYM C [15]. The signals for the sidechain between ring C and D are underlined. GYM, gymnodimine derivative.

	1			GYM D [8]			2			GYM B [14]			GYM C [15]	
No.	^13^C	^1^H		^13^C	^1^H		^13^C	^1^H		^13^C	^1^H		^1^H	
1	175.5			175.2						174.7				
2	130.2			130.2						130.3				
3	148.6	7.05		148.5	6.96		149.8	6.93		147.1	6.91		6.91	
4	81.6	5.93		81.1	5.95		81	5.88			5.84		5.85	
5	125.9			125.7						125.2				
6	136.0	by HMBC		137.4						132.8				
7	43.6	3.16		44.2	3.09		44.5	3.11			3.63		3.63	
8	31.8	1.9	1.43	30.9	1.82	1.43	30.3	1.74	1.22	125.9	5.28		5.31	
9	71.6	3.66		72.5	3.96		74.9	3.66		140.4				
10	83.4	3.94		84.3	4.02		84.5	3.92		80	3.94		3.94	
11	27.0	1.79	1.56	28.4	1.99	1.85	29	1.74			2.08	1.48	1.97	1.57
12	24.9	1.77		25.9	1.85		25.2	1.73	1.52		1.4	1.15	1.36	1.17
13	78.6	4.36		80.3	4.27		81.1	4.12			4.09		4	
14	82.5	4.13		78.7	4.09		81.6	3.89		34.8	1.77–1.82		1.78	1.71
15	29.4	1.99	1.76	34.5	1.91	1.22	26.4	1.76	1.56	41.1	2.71		2.65	
16	32.1	1.79		36	2.3	2.3	29.7	1.84		90.9	3.85		3.89	
17	82.9	4.15		84.5	4.09		82.1	4.21						
18	133.1			129.7						81.7	4.11		3.97	
19	125	5.99		127.8	5.98		73.4	4.58			2.4	1.49	2.06	1.57
20	21.9	3	2.1	21.8	3	2.14	36.9	2.36			2.64	2.23	2.62	2.21
21	31.8	2.76		32.1	2.64	2.38	32.4	2.79						
22	173.3			172.8										
23	42.7			43.5							1.77	1.54	1.77	1.57
24	33.6	1.59	1.37	33.6	1.64	1.33	30.3	1.51	1.3		2.06	1.54	2.06	1.57
25	19.7	1.49		19.8	1.93	1.53	19.9	1.88	1.42		1.96		1.96	
26	11.0	1.99		10.7	1.96		10.7	1.85			1.71		1.71	
27	18.1	1.92		17.9	2.06		17.3	2.03			1.91		1.78	
28				16.7	0.86						0.96		0.98	
29	15.3	1.61		15.4	1.56		109.2	5.69	5		5.32	5.19	5.18	4.99
30	26.0	1.54	1.44	26.9	1.52	1.44	25.2	1.72	1.62		1.95	1.54	1.91	1.57
31	20.5	1.47		20.3	1.44		20.2	1.36	1.24		1.54	1.54	1.57	1.57
32	50.3	3.71	3.48	50.1	3.73	3.51	50.3	3.75	3.3		3.57	3.4	3.52–3.45	

**Table 4:** Proton and carbon chemical shift of SPX A, SPX C, 13-desmethyl SPX C (all in CD_3_OD by Hu et al.), 20-hydroxy-13,19-didesmethyl-SPX D (**11**, CD_3_OD), and 20-hydroxy-13,19-didesmethyl-SPX C (**10**, recorded in CD_3_OD and C_5_D_5_N); * was not detected in MeOD.

	10 (CD_3_OD)			10 (C_5_D_5_N)			11			SPX A [13]			SPX C [13]			13-DesMe SPX C [13]		
No.	^13^C	^1^H		^13^C	^1^H		^13^C	^1^H		^13^C	^1^H		^13^C	^1^H		^13^C	^1^H	
1	176.7			175.7			182.3			177.1			177.1			176.8		
2	130.7			130			36.3	2.84		130.8			130.7			131		
3	149.5	7.18		148.3	7		35.7	2.58	1.69	150	7.12		149.9	7.12		149.5	7.13	
4	82	6		80.9	5.82		79	5.43		82.5	5.94		82.5	5.95		82	5.98	
5	126			124.9			129.7			125.9			126			126.4		
6	133.4			133.5			131			134.9			134.7			133.2		
7	48.2	3.76		47.6	3.39		47.6	3.72		48.8	3.57		49.1	3.56		48.1	3.78	
8	123.3	5.22		123.6	5.25		123.4	5.21		124.4	5.34		124.2	5.2		122.5	5.16	
9	145.9			143.5			144.8			144.5			144.6			146		
10	76.5	4.1		76.4	4.38		76.6	4.09		76.7	4.16		76.8	4.15		76.8	4.15	
11	45.4	2.34	1.35	45.4	2.73	1.66	44.8	2.34	1.34	39.7	1.61	2.14	39.6	1.57	2.14	45.2	1.37	2.25
12	79.6	4.37		79.4	4.71		79.6	4.37		81.7	4.33		81.7	4.31		79.8	4.3	
13	32.2	2.33	1.66	31.8	2.16		32	2.32	1.66	35.3	2.42		35.4	2.41		32.8	1.7	2.27
14	37.4	2.31	2.03	37.2	1.96	1.77	37.2	2.31	2.02	45.7	2.13	2.26	45.8	2.14	2.26	38.2	1.95	2.29
15	118.5			117.0			118.8			117.3			117.4			118.1		
16	34.7	2.1		34.8	2.3	2.1	34.6	2.1		36.6	2.04	2.19	36.5	2.04	2.22	35.2	2.07	2.21
17	35.8	2.29	2.03	35.5	2.5	2.19	36.2	2.3	2	31.5	1.76	2.14	31.5	1.74	2.11	32.1	1.79	2.2
18	110.7			110.3			110.9			112.5			112.5			112.2		
19	71.4	3.45		71.3	3.68		71.2	3.46		71.2			71.1			71.1		
20	69.7	3.96		69.3	4.18		69.8	3.95		35.8	1.47	1.84	35.8	1.49	1.81	35.7	1.49	1.81
21	38.3	1.95	1.41	38.4	2.06	1.36	37.8	1.96	1.4	30.2	1.23	1.59	30.2	1.24	1.55	29.9	1.28	1.58
22	64.6	4.17		63.7	4.54		64.4	4.16		69.4	4		69.3	3.97		69.1	3.97	
23	46.3	2.42	2.14	46.9	2.69	2.29	46.2	2.42	2.14	47.5	2.02	2.34	47.6	2.01	2.37	46.3	2.06	2.41
24	145.4			147.5			146			147.8			147.8			145.6		
25	36.4	1.99		35.6	2.55	1.75	37.1	2.04		35.9	1.6	2.12	36	1.58	2.1	34.6	1.83	2.05
26	24.3	1.96	1.7	23.0	2.47	1.46	24	1.94	1.7	23.7	1.39	2.02	23.4	1.4	2.02	21.8	1.83	2.01
27	*	*	*	34.9	2.3	2.1	35.8	2.25		35.6	2.34	2.41	35.6	2.32	2.43	36	2.82	3.1
28	*			174.4			181			179.3			178.6			201.3		
29	51.6			52.7			52.1			51.4			50.8			52.4		
30	36.9	1.95	1.75	37.5	1.47		36.4	1.97	1.76	28	1.65	1.9	38.3	1.55	1.73	36.7	1.79	2.01
31	37	1.08		35.8	1.22		36.7	1.09		32.1	1.06	1.78	36.9	1.16		37.5	1.04	
32	39.2	1.58		40.6	1.25		39.2	1.58		33.6	1.88		41.2	1.36		38.8	1.67	
33	52	4.07	3.57	52.8	3.67	3.61	51.9	4.1	3.57	53.1	3.48	3.72	53.3	3.44	3.76	51.8	3.55	4.18
34	31.8	1.96	1.78	31.4	1.64	1.32	33.2	1.82		32.2	1.55	1.8	32.4	1.52	1.8	32.4	1.67	1.98
35	20	2.25	1.67	19.2	2.07	1.47	20.2	2.41	2.09	20.4	1.56	2.11	20.3	1.51	2.14	20.3	1.72	2.27
36	10.2	1.93		10.7	1.85		14.5	1.26		10.4	1.88		10.4	1.86		10.5	1.9	
37	16.6	1.77		16.7	1.53		16.4	1.67		17	1.71		17.1	1.72		16.7	1.74	
38	12.3	1.92		12.6	1.98		12.2	1.92		12.2	1.85		12.3	1.87		12.9	1.91	
39										15.8	1.2		15.6	1.19				
40										22.5	1.19		22.5	1.19		22.7	1.2	
41	113.8	4.92	4.89	110.6	4.83	4.81	113.5	4.93		111.4	4.77	4.75	111.3	4.75	4.78	112.6	4.81	4.92
42	19.1	1.09		20.1	1.29		19.2	1.09		21	0.92		19.4	0.98		18.9	1.05	
43	20	1.09		20.0	0.85		19.2	1.09					21.1	0.95		20.1	1.11	

**Table 5:** Exact and measured accurate masses (*m*/*z*) for [M + H]^+^ at *m*/*z* 694 and *m*/*z* 696 and their product ions obtained with LC-HRMS.

10				11			
Formula	Measured	Calculated	Δ/ppm	Formula	Measured	Calculated	Δ/ppm
C_41_H_62_O_8_N	696.44727	696.4470	0.4	C_41_H_60_O_8_N	694.4307	694.4313	−0.98
C_41_H_60_O_7_N	678.4365	678.4364	0.14	C_41_H_58_O_7_N	676.4203	676.4208	−0.64
C_41_H_58_O_6_N	660.4261	660.4259	0.34	C_41_H_56_O_6_N	658.4100	658.4102	−0.37
C_41_H_56_O_5_N	642.4155	642.4153	0.35	C_41_H_54_O_5_N	640.3995	640.3997	−0.27
C_41_H_54_O_4_N	624.4050	624.4047	0.47	C_41_H_52_O_4_N	622.3890	622.3891	−0.17
C_26_H_42_O_6_N	464.3011	464.3007	0.84	C_26_H_42_O_6_N	464.3007	464.3007	0.12
C_26_H_40_O_5_N	446.2903	446.2901	0.55	C_26_H_40_O_5_N	446.2900	446.2901	−0.14
C_26_H_38_O_4_N	428.2798	428.2795	0.73	C_26_H_38_O_4_N	428.2794	428.2795	−0.2
C_26_H_36_O_3_N	410.2692	410.2690	0.47	C_26_H_36_O_3_N	410.2689	410.269	−0.2
C_26_H_34_O_2_N	392.2585	392.2584	0.35	C_26_H_34_O_2_N	392.2584	392.2584	0.04
C_18_H_30_O_2_N	292.2271	292.2271	0.13	C_18_H_30_O_2_N	292.2269	292.2271	0.7
C_18_H_28_ON	274.2166	274.2165	0.04	C_18_H_28_ON	274.2164	274.2165	−0.51
C_16_H_26_ON	248.2009	248.2009	0.21	C_16_H_26_ON	248.2008	248.2009	−0.41
C_16_H_24_N	230.1904	230.1903	0.45	C_16_H_24_N	230.1903	230.1903	−0.21
C_14_H_22_N	204.1748	204.1747	0.8	C_14_H_22_N	204.1747	204.1747	−0.04
C_11_H_18_N	164.1435	164.1434	0.876	C_11_H_18_N	164.1434	164.1434	0.16

**Table 6:** Concentrations of GYM A (**4**), 16-desmethylgymnodimine D (**1**), Gymnodimine E (**2**), SPX 1 (**9**), 20-Hydroxy-13,19-didesMethyl-SPX C (**10**), and 20-Hydroxy-13,19-didesMethyl-SPX D (**11**) in a re-analysis of plankton net samples collected from three stations during previous study by van der Waal et al. [25].

Station	GYM A	1	2	SPX 1	10	11
	All in pg per mL Filtered Sea Water					
SL92-1	204	160	619	61	2	15
SL92-2	561	447	1250	211	8	40
SL92-3	2	1	3	0	0	0

## Supplementary Materials

The following are available online at http://www.mdpi.com/1660-3397/16/11/446/s1, Figure S1: Structures of known and novel gymnodimines; Figure S2: Structures of known and novel spirolides; Figures S4–S22 NMR-spectra of **1**; Figure S23: CD-spectra of **1** and **4** and simulated CD spectra of **1,** S24-S31 NMR-spectra of **2**; Figures S32–S53 NMR-spectra of **10**; Figures S54–S62 NMR-spectra of **11**; Table S1: Mass transitions of spiroimines included in LC-MS/MS analysis; Figure S63: LC-MS/MS chromatogram of station SL92-2. Raw NMR data (Topspin) and annotated Mestre files are available in the data repository PANGAEA https://doi.pangaea.de/10.1594/PANGAEA.895116.
