# Supplementary material for: Identification of Novel Gymnodimines and Spirolides from the Marine Dinoflagellate Alexandrium ostenfeldii
Source: Mar Drugs. 2018 Nov 14;16(11):446. doi: 10.3390/md16110446 (PMC6266918; doi:10.3390/md16110446)
Supplement: Supplementary file 1 [file marinedrugs-16-00446-s001.pdf]

Identification of novel gymnodimines and spirolides from the marine dinoflagellate *Alexandrium ostenfeldii*

Supporting Information

Christian Zurhelle, Joyce Nieva, Urban Tillmann, Tilmann Harder, Bernd Krock*, Jan Tebben*

All raw NMR data (Topspin) and annotated Mestre files are available in the data repository PANGAEA
https://doi.pangaea.de/10.1594/PANGAEA.895116

Figure S1: Structures of known and novel gymnodimines. 3
Figure S2: Structures of known and novel spirolides. 4
Figure S3: 1D Proton spectra of **1** (600 MHz, pyridine-d_5). 5
Figure S4: 1D ^{13}C-spectra of **1**. 6
Figure S5: Multiplicity-edited 2D HSQC spectra of **1**. 7
Figure S6: Slice 1 of multiplicity-edited 2D HSQC spectra of **1**. 8
Figure S7: Slice 2 of multiplicity-edited 2D HSQC spectra of **1**. 9
Figure S8: 2D COSY spectra of **1**. 10
Figure S9: Slice 1 of 2D COSY spectra of **1**. 11
Figure S10: Slice 2 of 2D COSY spectra of **1**. 12
Figure S11: 2D double quantum filtered COSY spectra of **1**. 13
Figure S12: 2D TOCSY spectra of **1**. 14
Figure S13: Slice 1 of 2D TOCSY spectra of **1**. 15
Figure S14: Slice 2 of 2D TOCSY spectra of **1**. 16
Figure S15: HSQC-TOCSY spectra of **1**. 17
Figure S16: Slice 1 of HSQC-TOCSY spectra of **1**. 18
Figure S17: Slice 2 of HSQC-TOCSY spectra of **1**. 19
Figure S18: Slice 3 of HSQC-TOCSY spectra of **1**. 20
Figure S19: 2D IMPACT-HMBC spectra of **1**. 21
Figure S20: Slice 1 of 2D IMPACT-HMBC spectra of **1**. 22
Figure S21: Slice 2 of 2D IMPACT-HMBC spectra of **1**. 23
Figure S22: ROESY spectra of **1**. 24
Figure S23: Measured CD-spectra of **1** and **4** and simulated CD-spectra of **1** 25
Figure S24: 1D Proton spectra of **2**. 26
Figure S25: Multiplicity-edited 2D HSQC spectra of **2**. 27
Figure S26: Slice 1 of Multiplicity-edited 2D HSQC spectra of **2**. 28
Figure S27: Slice 2 of Multiplicity-edited 2D HSQC spectra of **2**. 29
Figure S28: 2D COSY spectra of **2**. 30
Figure S29: Slice of COSY spectra of **2**. 31
Figure S30: 2D HSQC-TOCSY spectra of **2**. 32
Figure S31: Slice of 2D HSQC-TOCSY spectra of **2**. 33
Figure S32: 1D Proton spectra of **10**. 34
Figure S33: 1D ^{13}C-spectra of **10**. 35
Figure S34: Slice of 1D ^{13}C-spectra of **10**. 36

Figure S35: 2D HSQC spectra of **10**. 37
Figure S36: COSY spectra of **10**. 38
Figure S37: 2D TOCSY spectra of **10**. 39
Figure S38: Slice of 2D TOCSY spectra of **10**. 40
Figure S39: HSQC-TOCSY spectra of **10**. 41
Figure S40: Slice of HSQC-TOCSY spectra of **10**. 42
Figure S41: HMBC spectra of **10**. 43
Figure S42: 1D Proton spectra of **10**. 44
Figure S43: 2D HSQC spectra of **10**. 45
Figure S44: Slice 1 of 2D HSQC spectra of **10**. 46
Figure S45: Slice 2 of 2D HSQC spectra of **10**. 47
Figure S46: 2D COSY spectra of **10**. 48
Figure S47: Slice of 2D COSY spectra of **10**. 49
Figure S48: 2D TOCSY spectra of **10**. 50
Figure S49: Slice of 2D TOCSY spectra of **10**. 51
Figure S50: 2D HSQC-TOCSY spectra of **10**. 52
Figure S51: Slice of 2D HSQC-TOCSY spectra of **10**. 53
Figure S52: 2D HMBC spectra of **10**. 54
Figure S53: Slice of 2D HMBC spectra of **10**. 55
Figure S54: 1D Proton spectra of **11**. 56
Figure S55: 2D HSQC spectra of **11**. 57
Figure S56: Slice of 2D HSQC spectra of **11**. 58
Figure S57: 2D COSY spectra of **11**. 59
Figure S58: Slice of 2D COSY spectra of **11**. 60
Figure S59: 2D TOCSY spectra of **11**. 61
Figure S60: Slice of 2D TOCSY spectra of **11**. 62
Figure S61: 2D HMBC spectra of **11**. 63
Figure S62: Slice of 2D HMBC spectra of **11**. 64
Table S1: Mass transitions of spiroimines included in LC-MS/MS analysis. 65
Figure S63: LC-MS/MS chromatogram of station SL92-2. 66

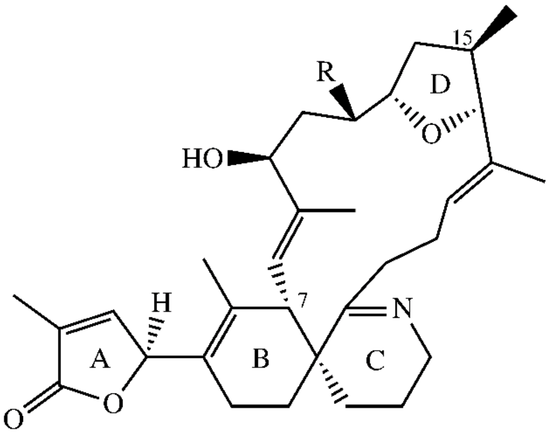

4 GYM A: R=H
7 12-methylGYM A: R=CH_3

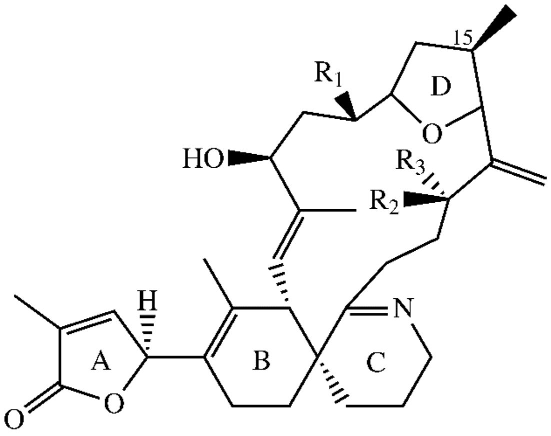

5 GYM B: R_1=H, R_2=H, R_3=OH
8 12-MethylGYM B: R_1=CH_3, R_2=H, R_3=OH
6 GYM C: R_1=H, R_2=OH, R_3=H

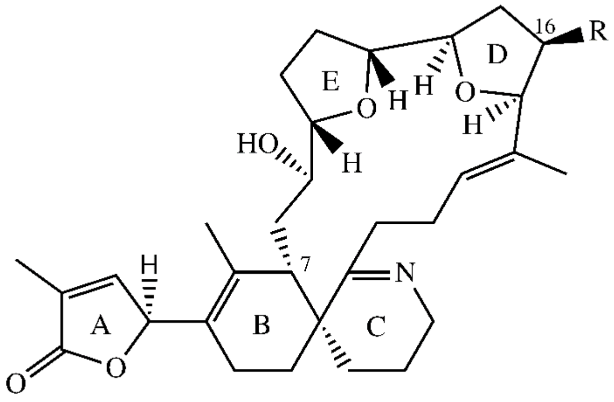

3 GYM D: R=CH_3
1 16-desmethylGYM D: R=H

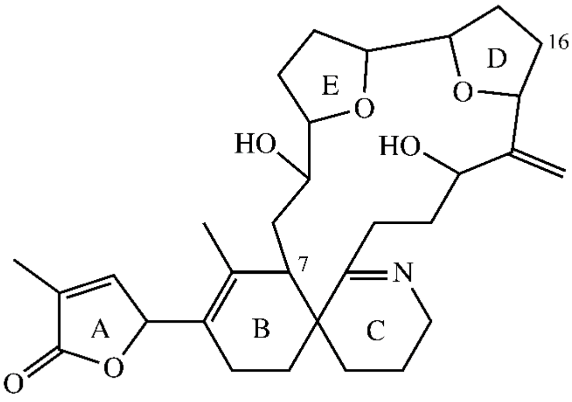

2 GYM E

Figure S1: Structures of known and novel gymnodimines.

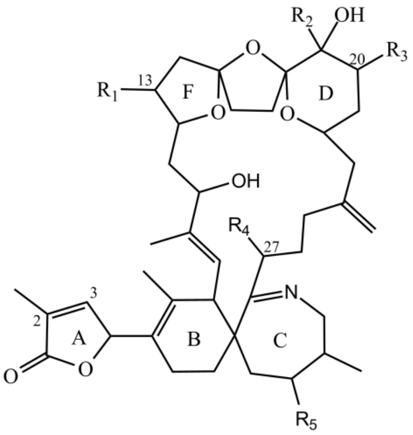

SPX A	$\Delta^{2,3}$ $R_{1,2} = CH_3$, $R_{3,4,5} = H$
SPX B	$R_{1,2} = CH_3$, $R_{3,4,5} = H$
SPX C	$\Delta^{2,3}$ $R_{1,2,5} = CH_3$, $R_{3,4} = H$
SPX D	$R_{1,2} = CH_3$, $R_{3,4,5} = H$
13-desMe SPX C (**9**)	$\Delta^{2,3}$ $R_{2,5} = CH_3$, $R_{1,3,4} = H$
27-OH-13-desMe SPX C	$\Delta^{2,3}$ $R_{2,5} = CH_3$, $R_{1,3} = H$, $R_4 = OH$
13,19-didesMe SPX C	$\Delta^{2,3}$ $R_5 = CH_3$, $R_{1,2,3,4} = H$
27-OH-13,19-didesMe SPX C	$\Delta^{2,3}$ $R_5 = CH_3$, $R_{1,2,3} = H$, $R_4 = OH$
27-Oxo-13,19-didesMe SPX C	$\Delta^{2,3}$ $R_5 = CH_3$, $R_{1,2,3} = H$, $R_4 = O=$
20-OH-13,19-didesMe SPX C (**10**)	$\Delta^{2,3}$ $R_5 = CH_3$, $R_{1,2,4} = H$, $R_3 = OH$
20-OH-13,19-didesMe SPX D (**11**)	$R_5 = CH_3$, $R_{1,2,4} = H$, $R_3 = OH$

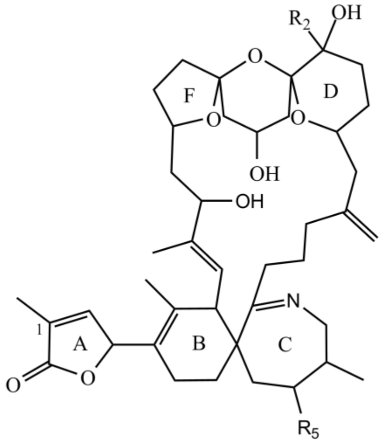

SPX G	$\Delta^{2,3}$ $R_5 = CH_3$, $R_2 = H$
20-Me SPX G	$\Delta^{2,3}$ $R_{2,5} = CH_3$

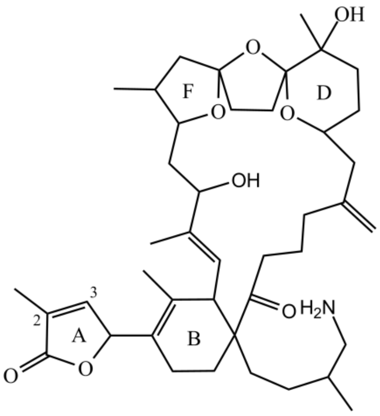

SPX E	$\Delta^{2,3}$
SPX F	

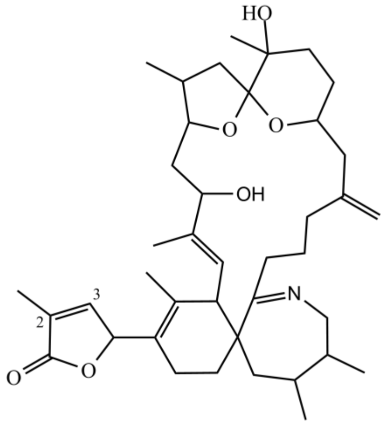

SPX H	$\Delta^{2,3}$
SPX I	

Figure S2: Structures of known and novel spirolides.

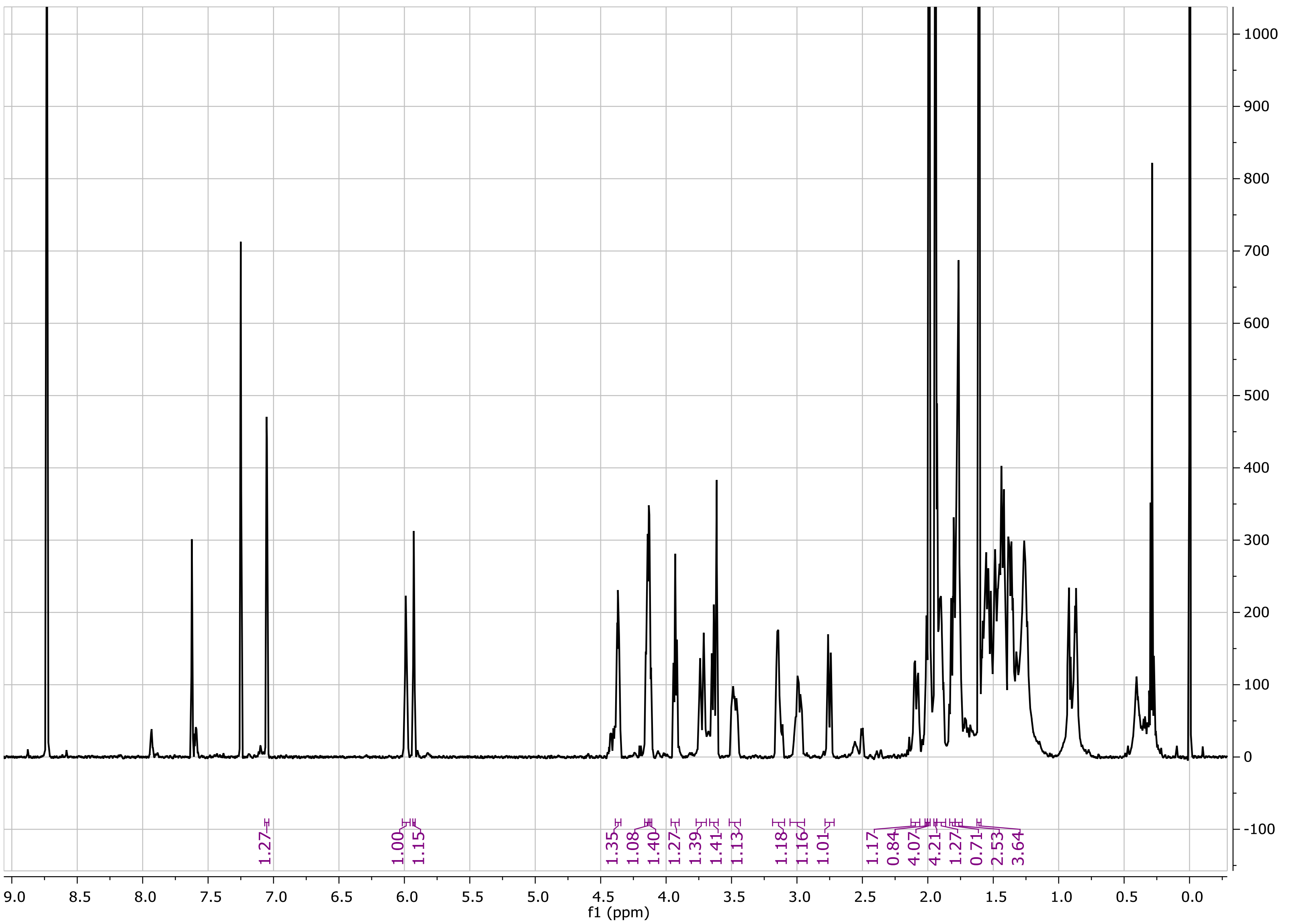

Figure S3: 1D Proton spectra of 16-Desmethylgymnodimine D (600 MHz, pyridine-d_5).

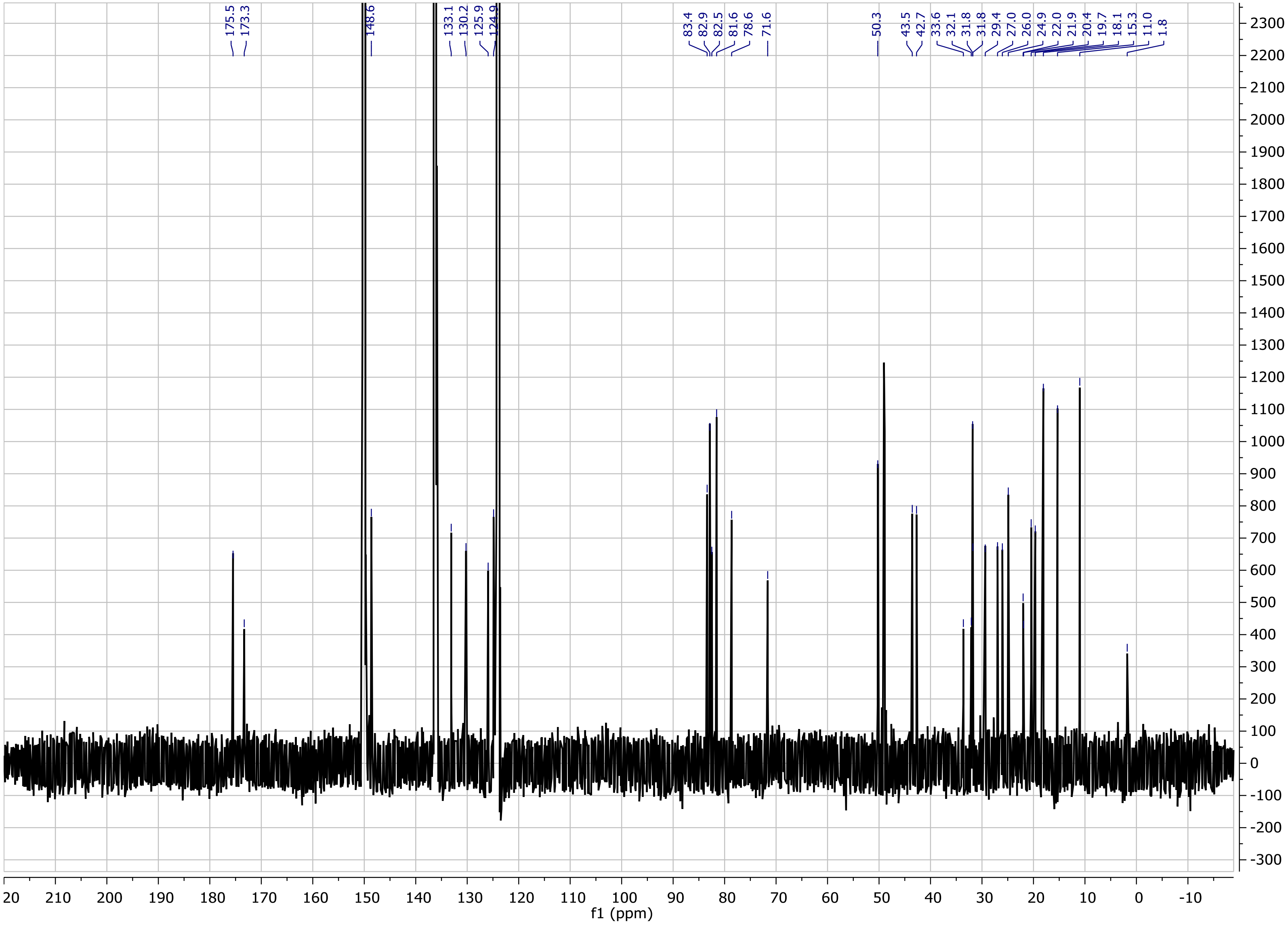

Figure S4: 1D ^{13}C-spectra of 16-Desmethylgymnodimine D (150 MHz, pyridine-d_5).

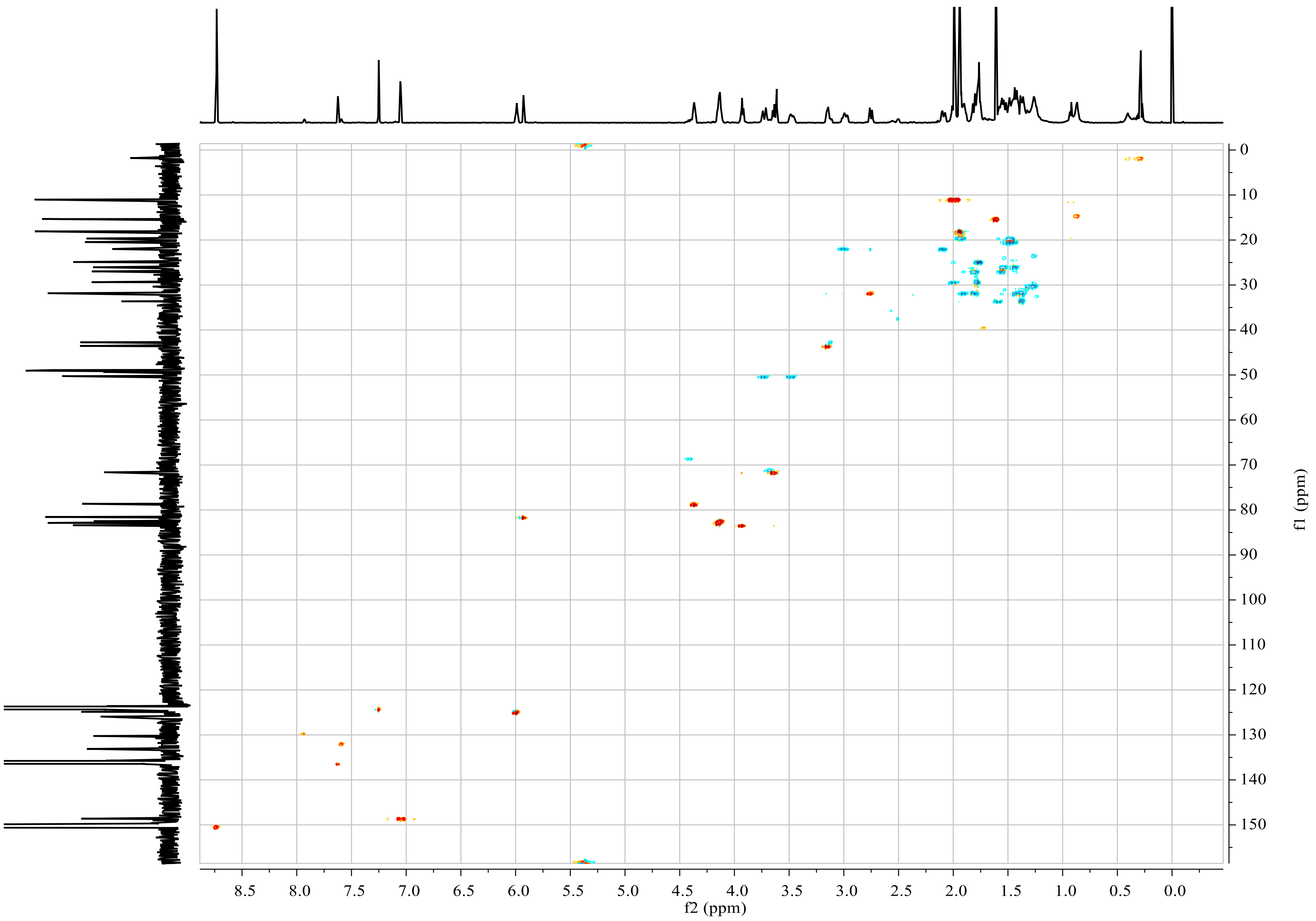

Figure S5: Multiplicity-edited 2D HSQC spectra of 16-Desmethylgymnodimine D (600 MHz Proton frequency, pyridine-d_5).

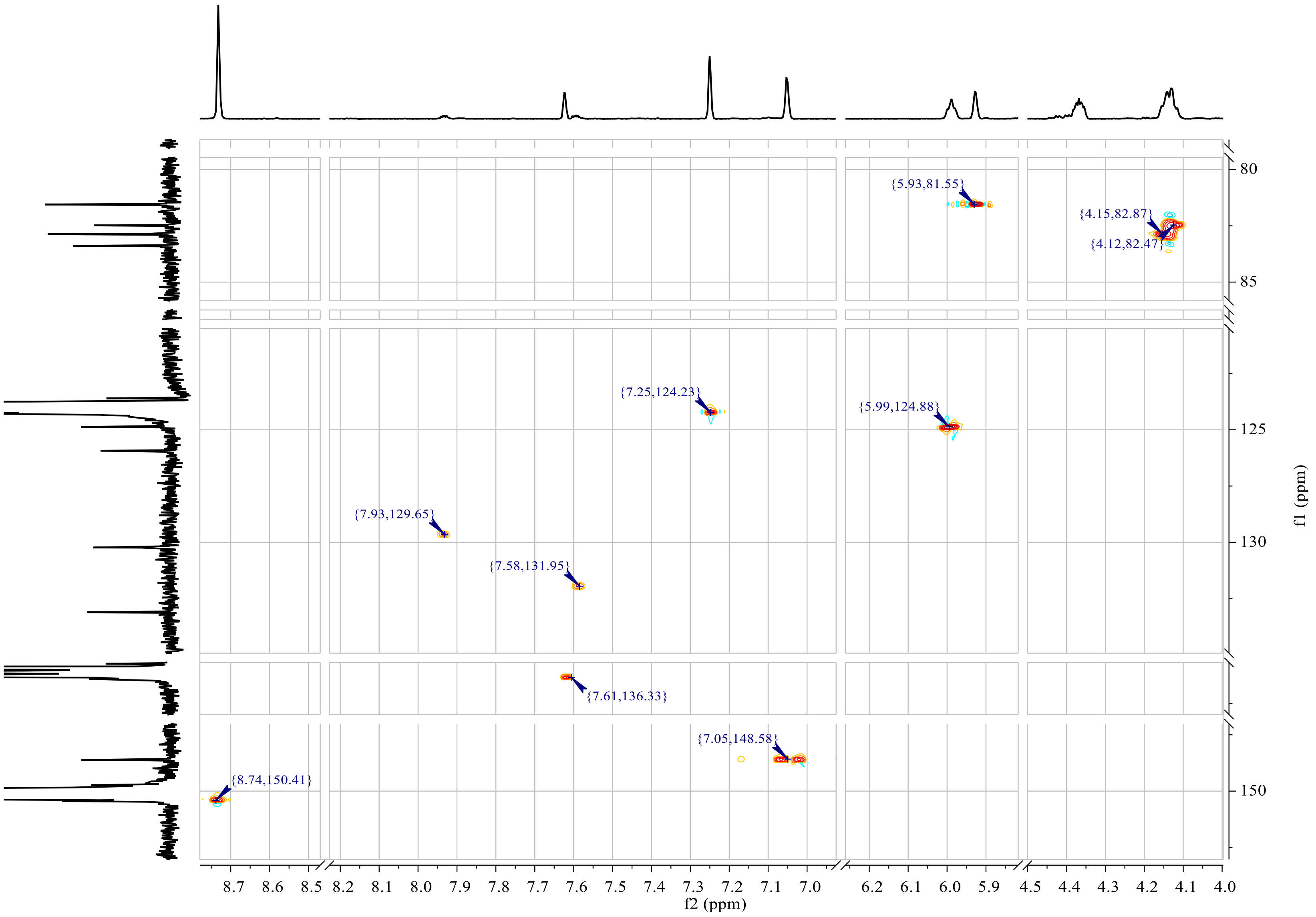

Figure S6: Slice 1 of multiplicity-edited 2D HSQC spectra of 16-Desmethylgymnodimine D (600 MHz Proton frequency, pyridine-d5).

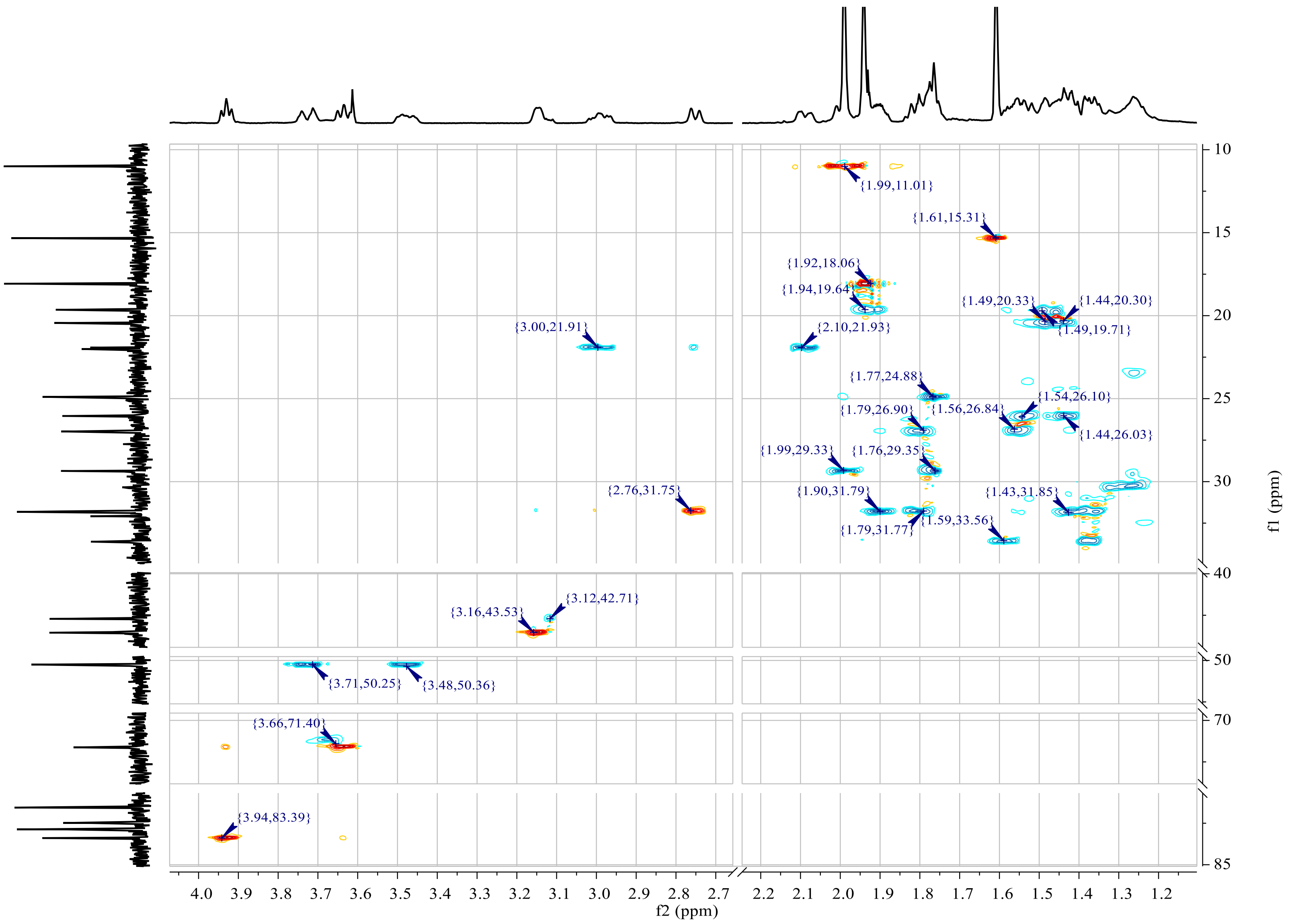

Figure S7: Slice 2 of multiplicity-edited 2D HSQC spectra of 16-Desmethylgymnodimine D (600 MHz Proton frequency, pyridine-d5).

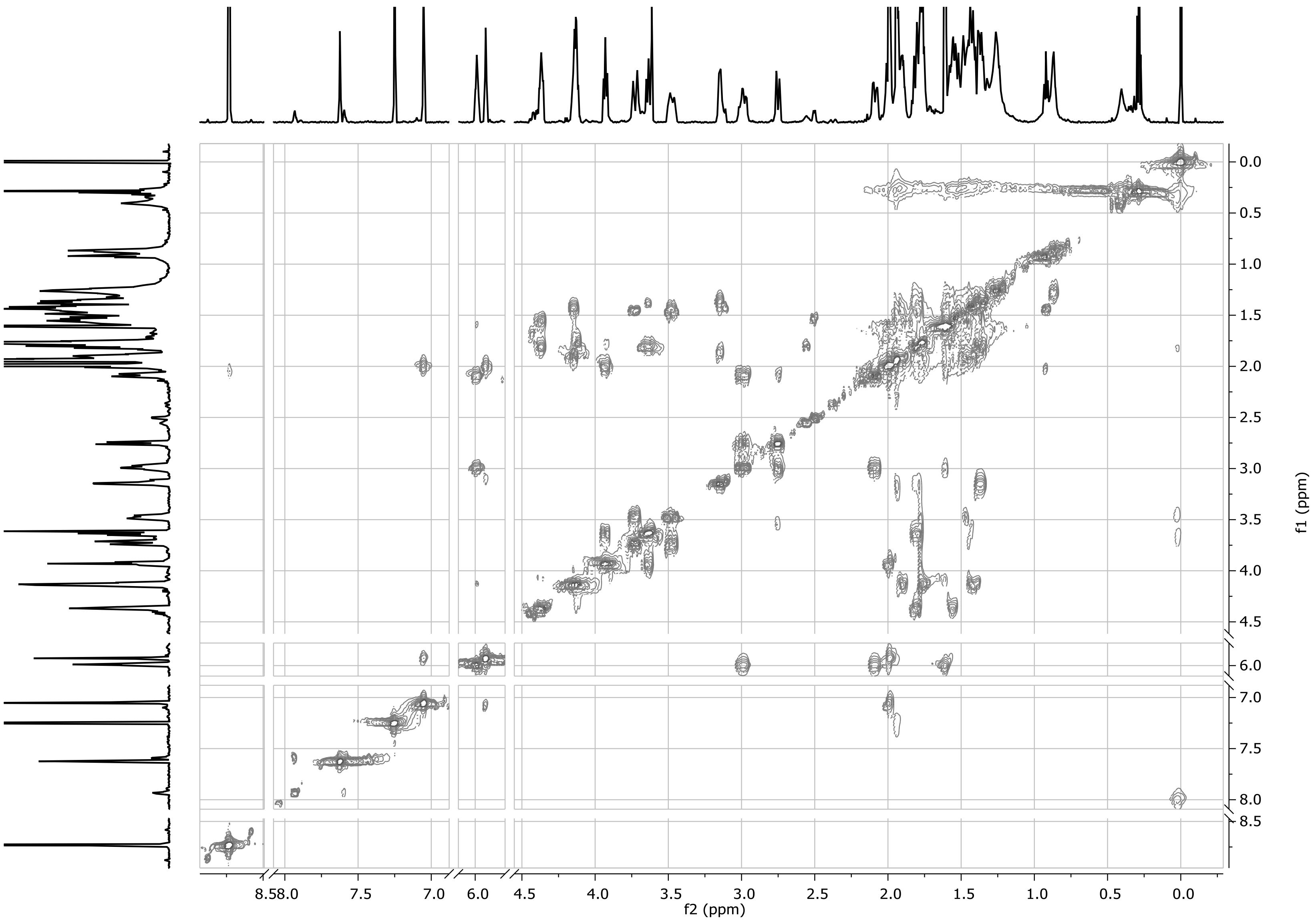

Figure S8: 2D COSY spectra of 16-Desmethylgymnodimine D (600 MHz, pyridine-d_5).

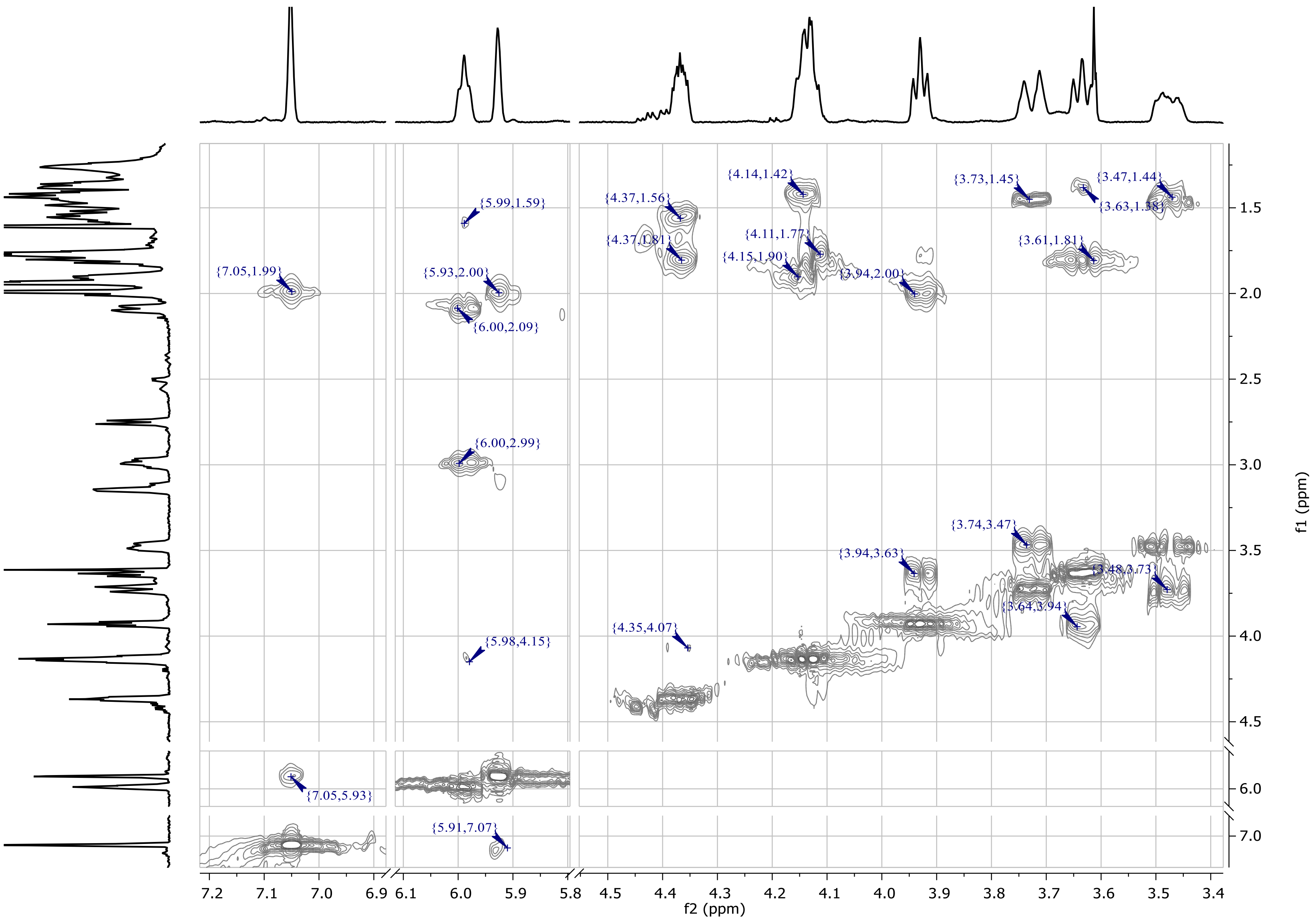

Figure S9: Slice 1 of 2D COSY spectra of 16-Desmethylgymnodimine D (600 MHz, pyridine-d_5).

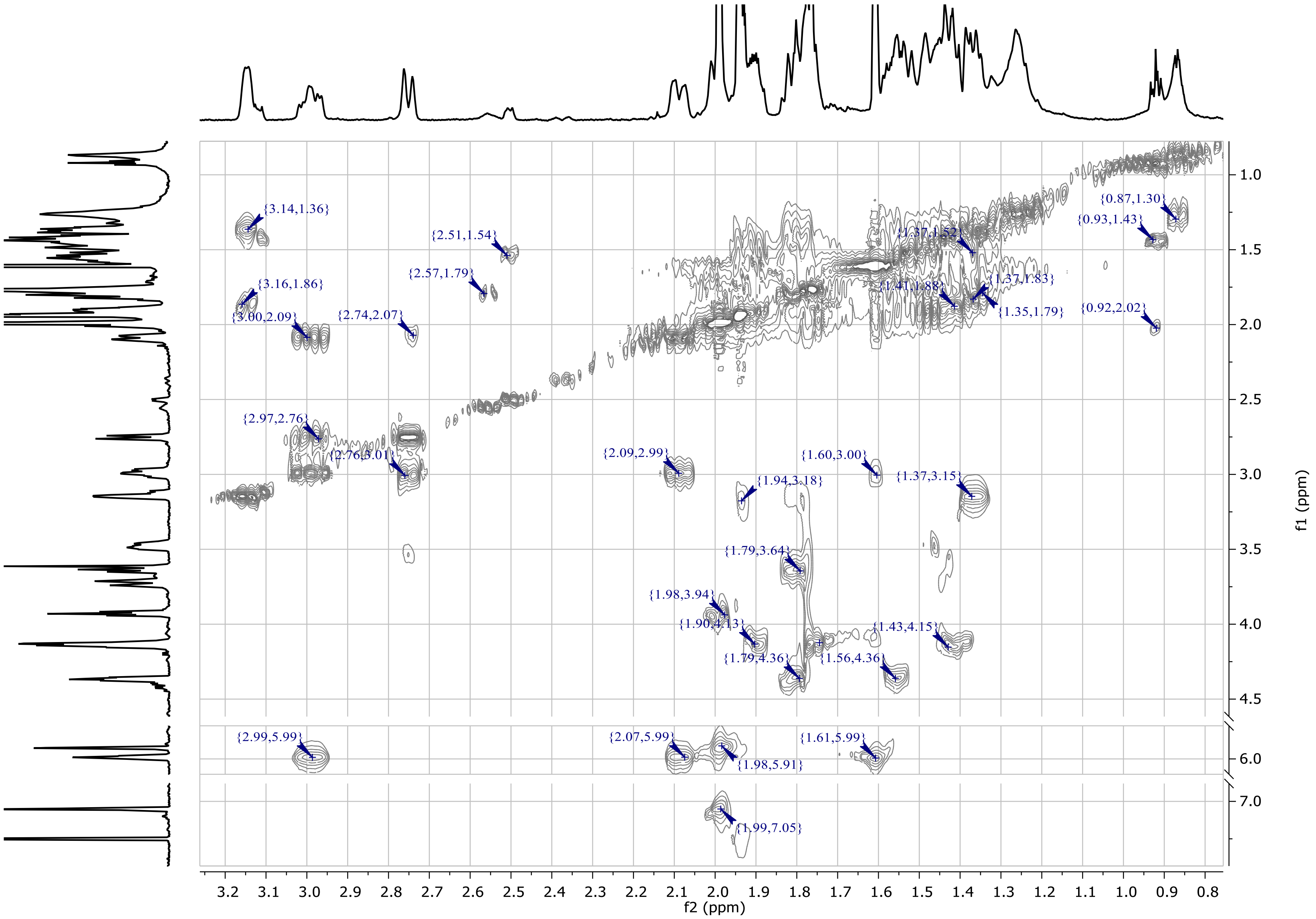

Figure S10: Slice 2 of 2D COSY spectra of 16-Desmethylgymnodimine D (600 MHz, pyridine-d_5).

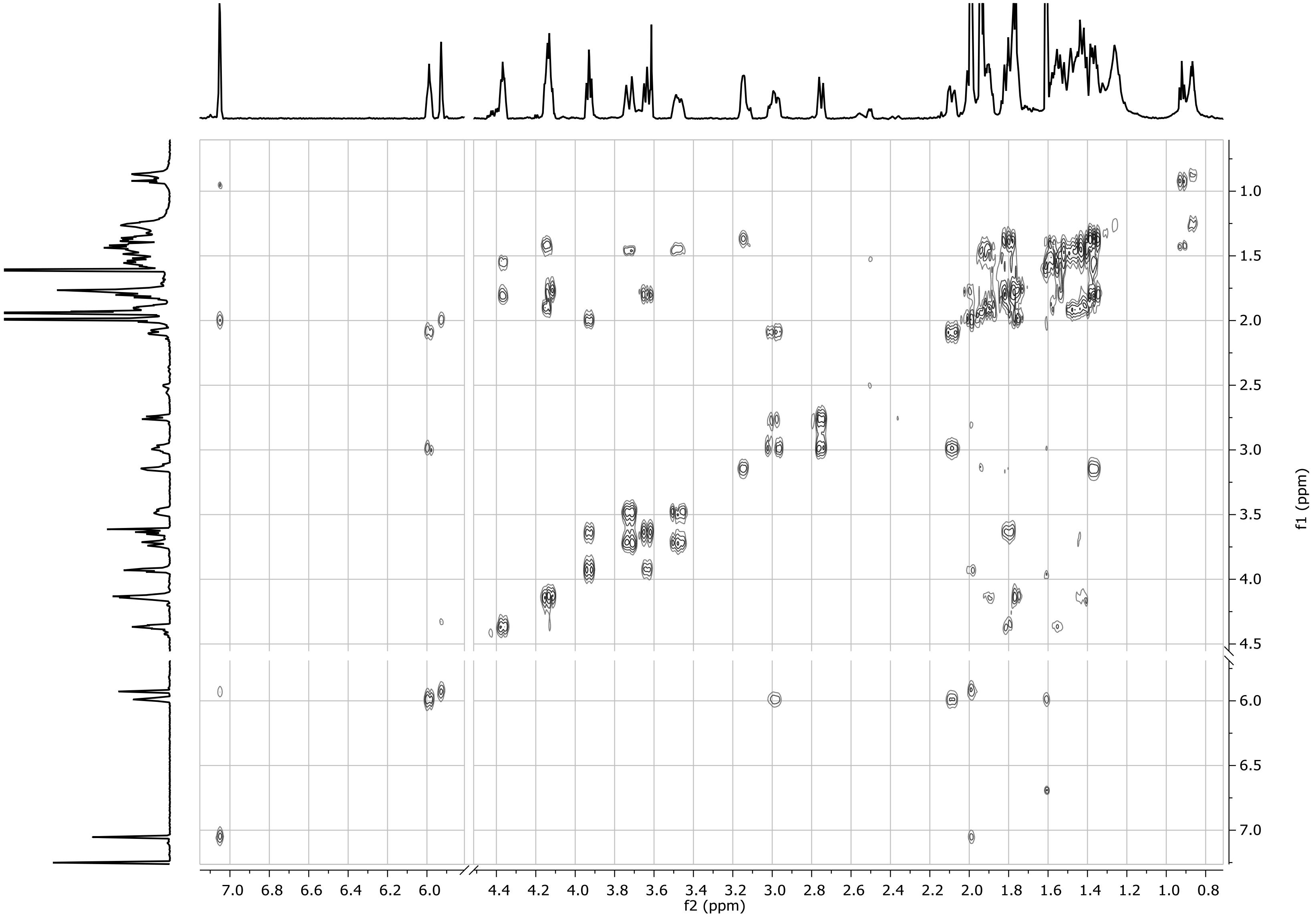

Figure S11: 2D double quantum filtered COSY spectra of 16-Desmethylgymnodimine D (600 MHz, pyridine-d_5).

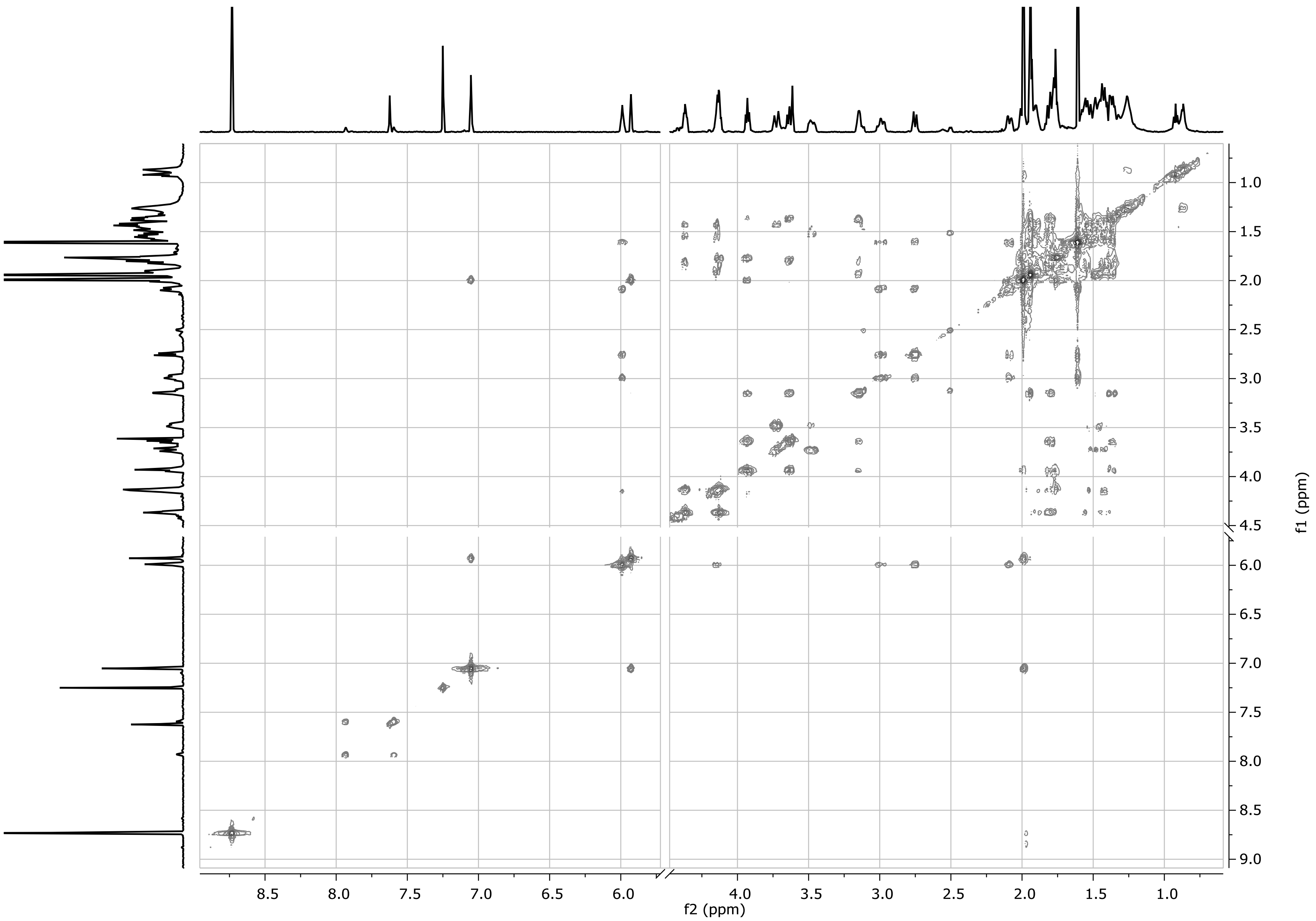

Figure S12: 2D TOCSY spectra of 16-Desmethylgymnodimine D (600 MHz, pyridine-d_5).

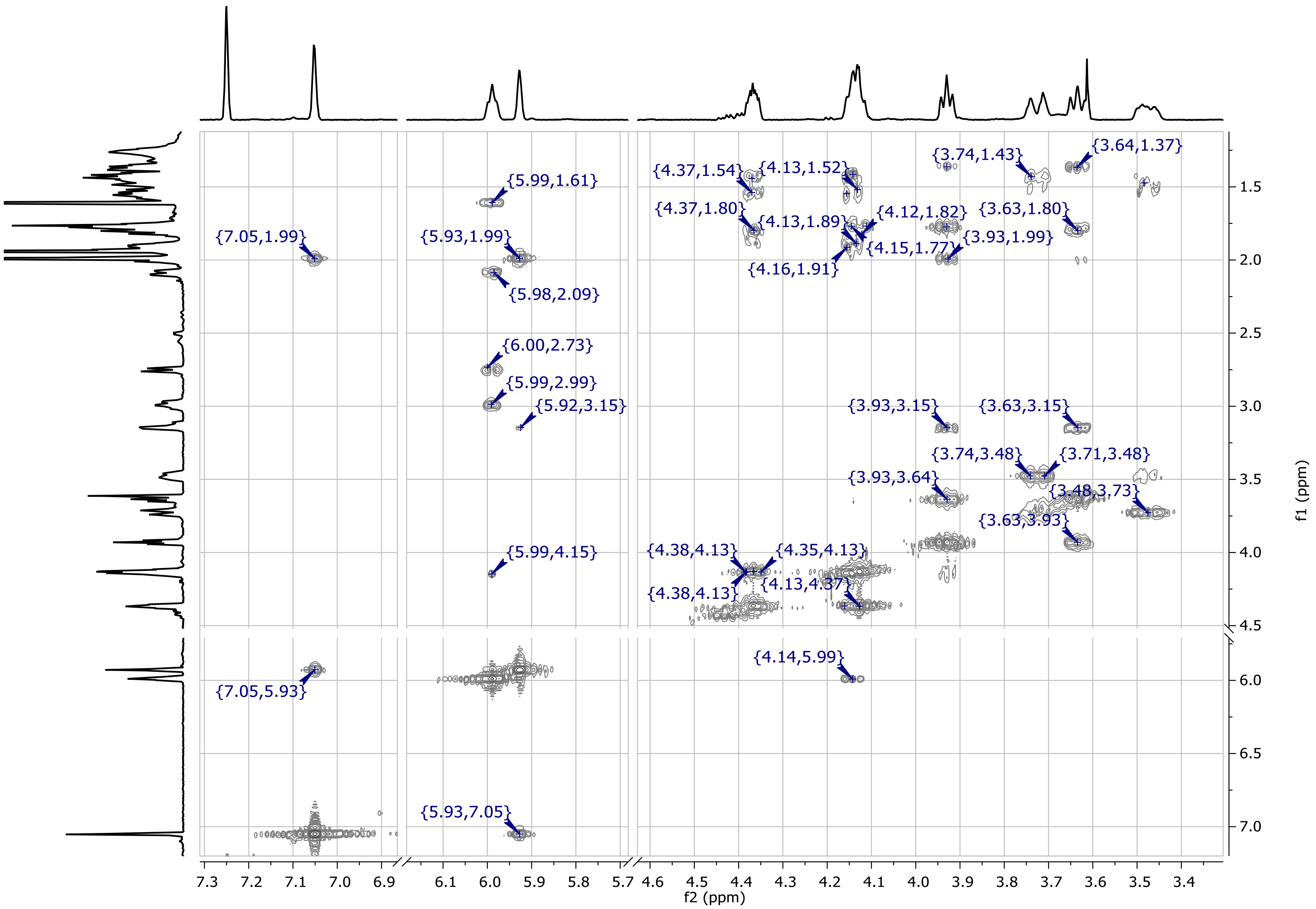

Figure S13: Slice 1 of 2D TOCSY spectra of 16-Desmethylgymnodimine D (600 MHz, pyridine-d5).

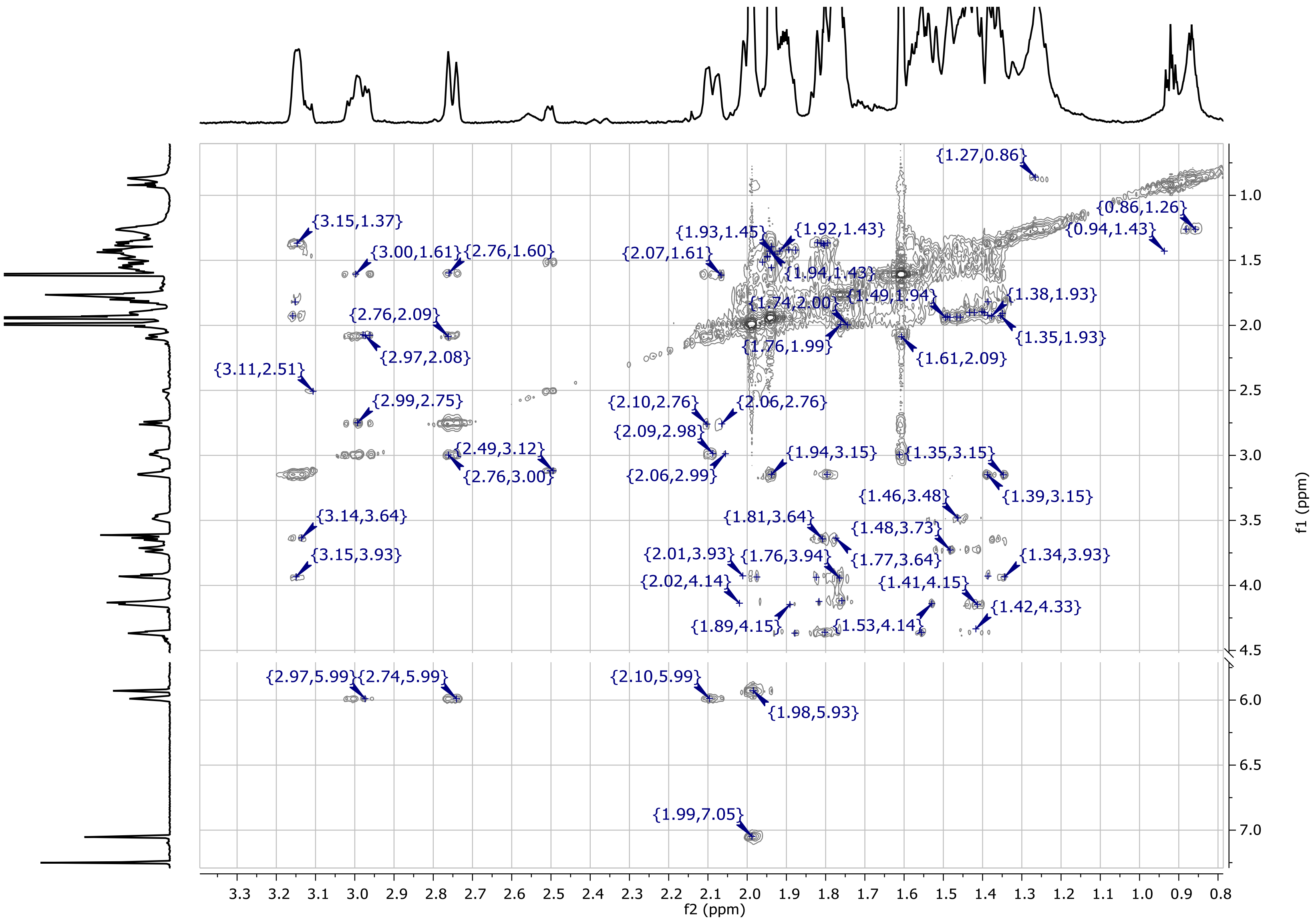

Figure S14: Slice 2 of 2D TOCSY spectra of 16-Desmethylgymnodimine D (600 MHz, pyridine-d$_5$).

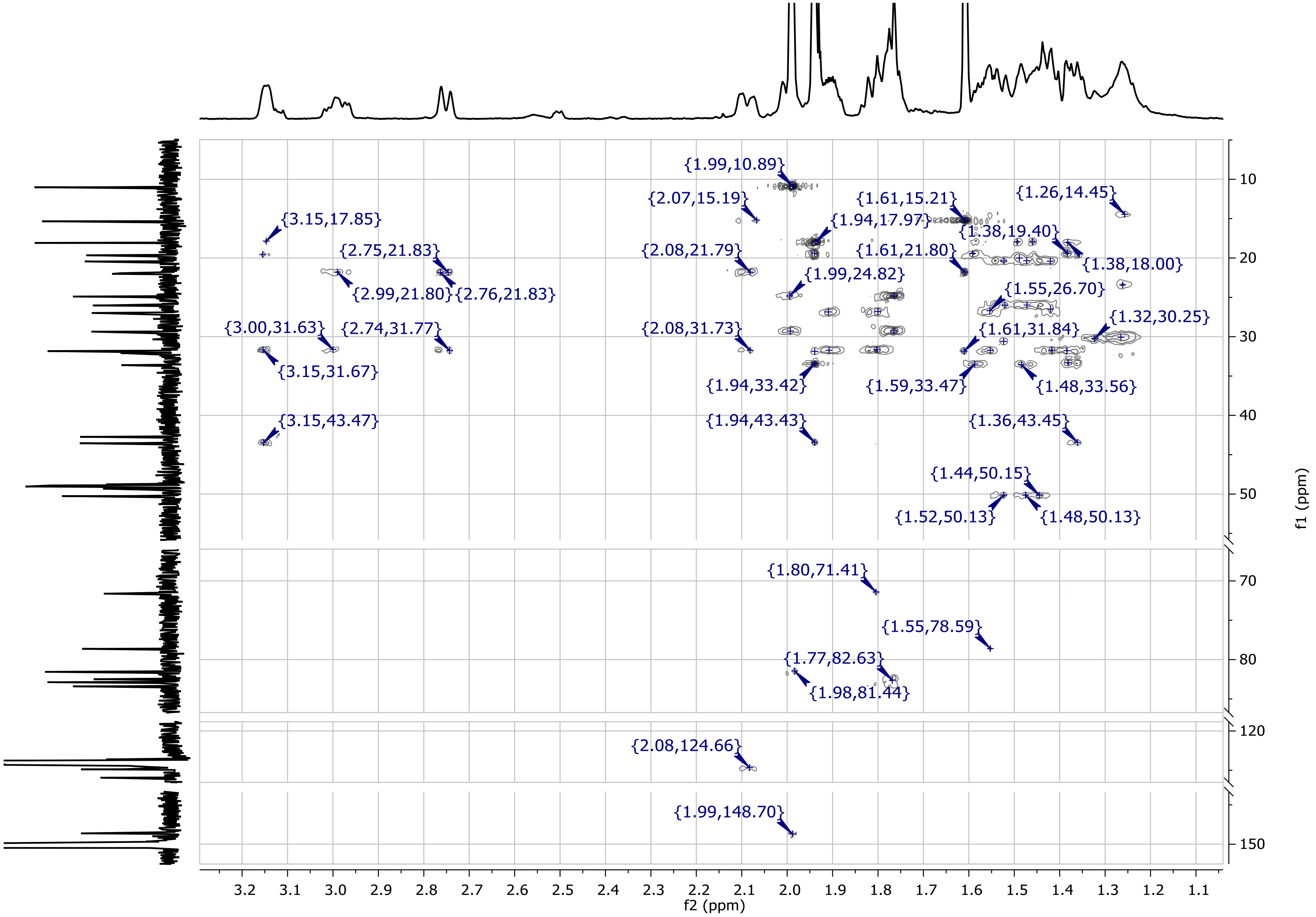

Figure S15: HSQC-TOCSY spectra of 16-Desmethylgymnodimine D (600 MHz Proton frequency, pyridine-d$_5$).

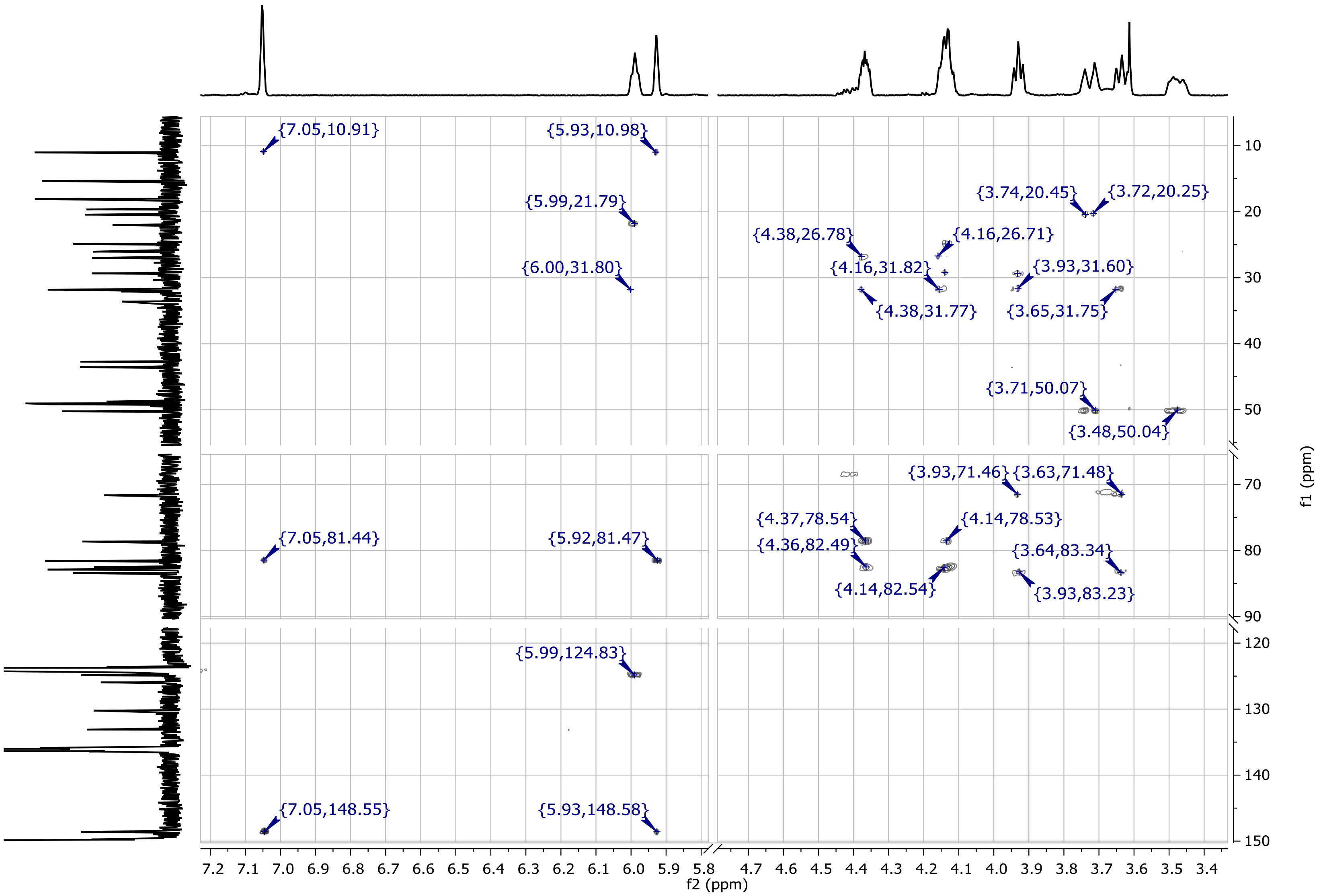

Figure S16: Slice 1 of HSQC-TOCSY spectra of 16-Desmethylgymnodimine D (600 MHz Proton frequency, pyridine-d$_5$).

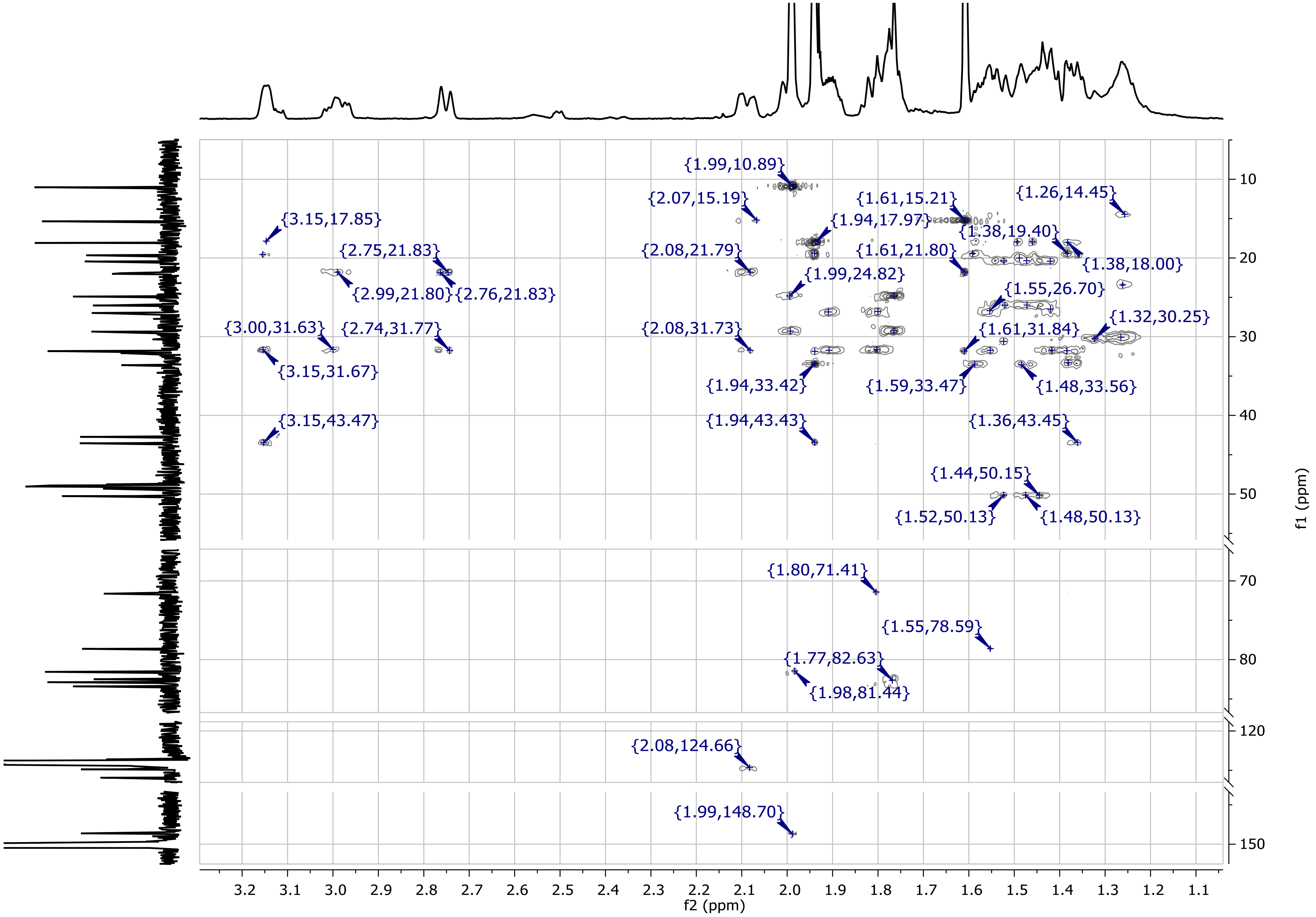

Figure S17: Slice 2 of HSQC-TOCSY spectra of 16-Desmethylgymnodimine D (600 MHz Proton frequency, pyridine-d_5).

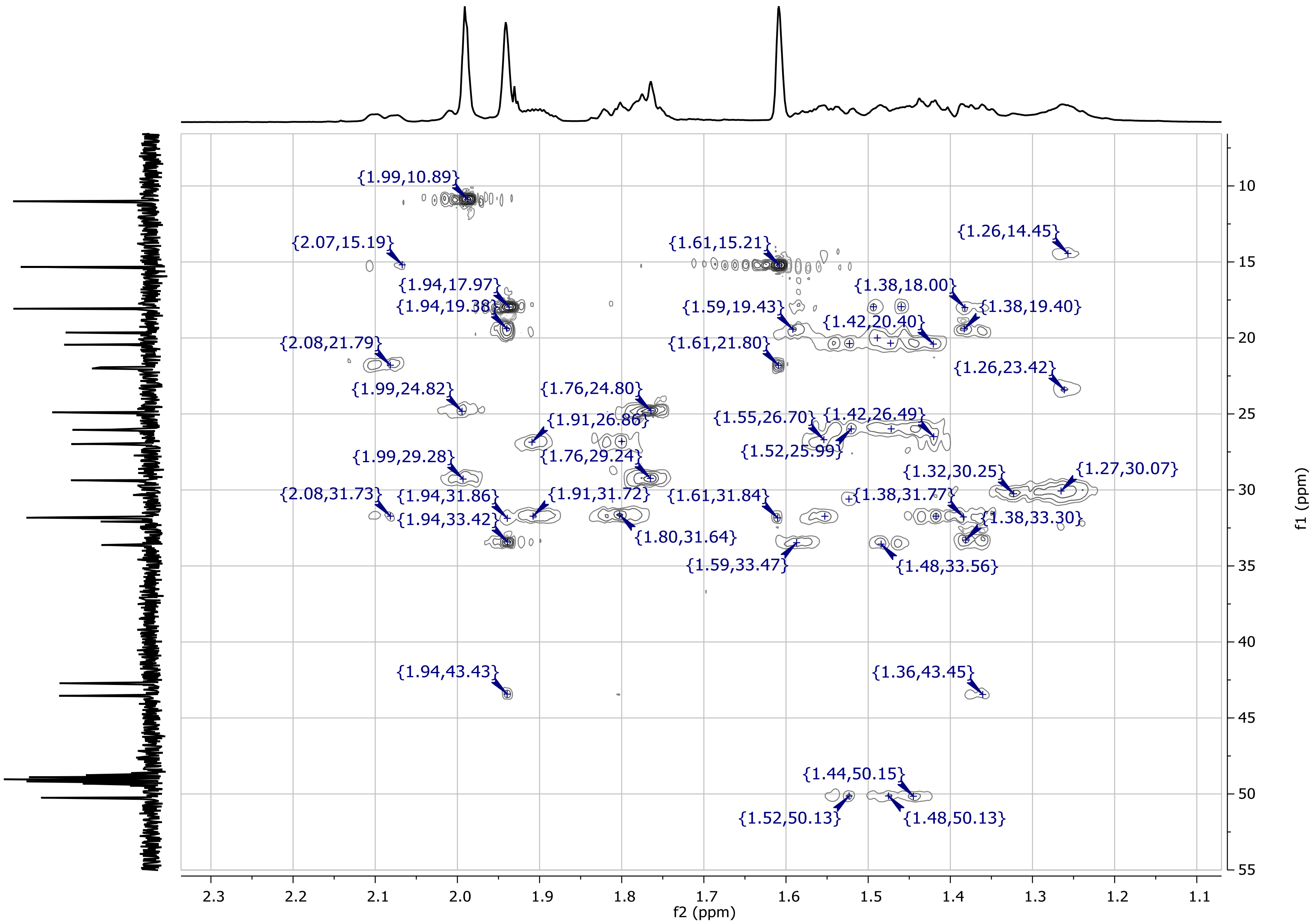

Figure S18: Slice 3 of HSQC-TOCSY spectra of 16-Desmethylgymnodimine D (600 MHz Proton frequency, pyridine-d5).

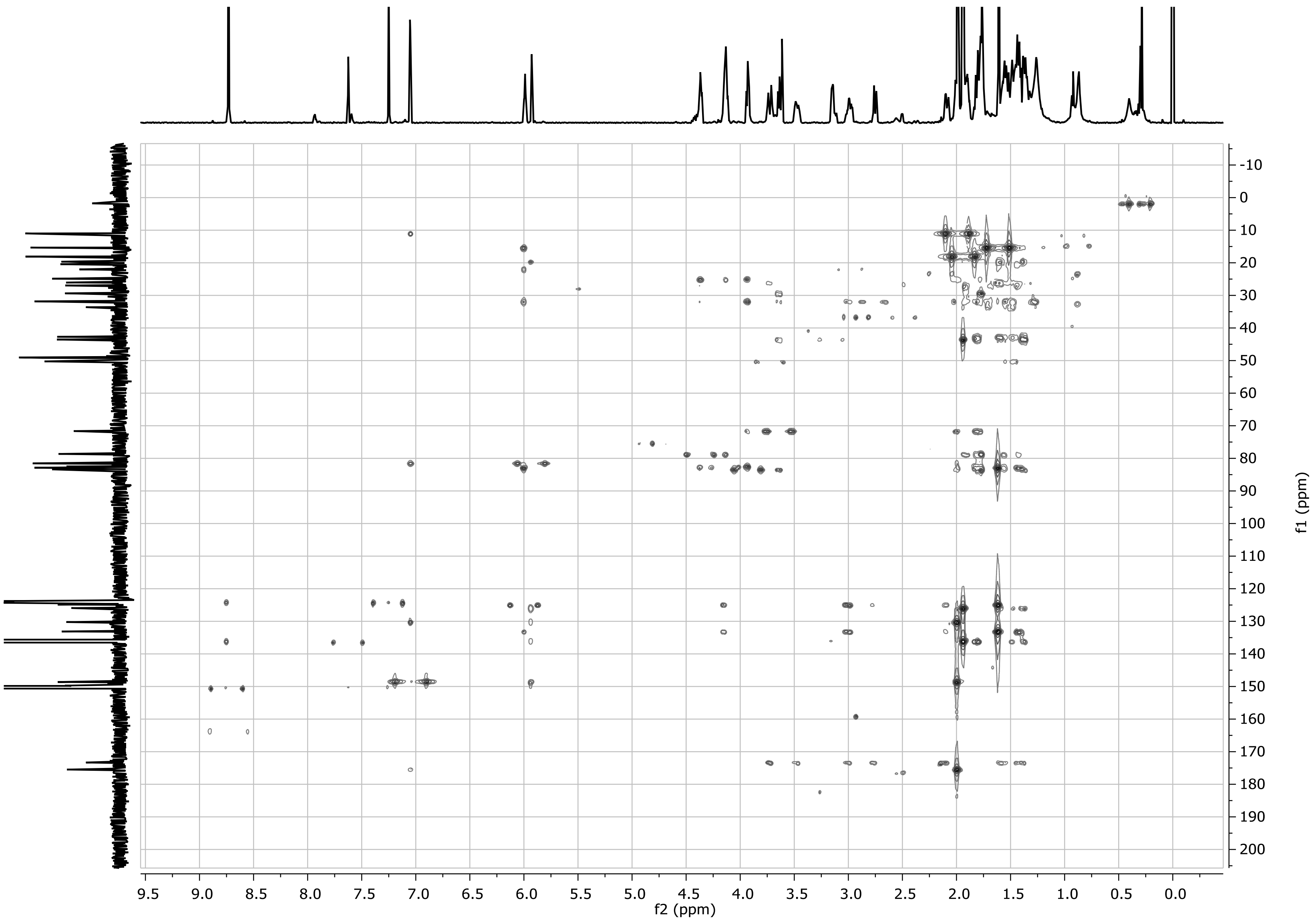

Figure S19: 2D IMPACT-HMBC spectra of 16-Desmethylgymnodimine D (600 MHz Proton frequency, pyridine-d_5).

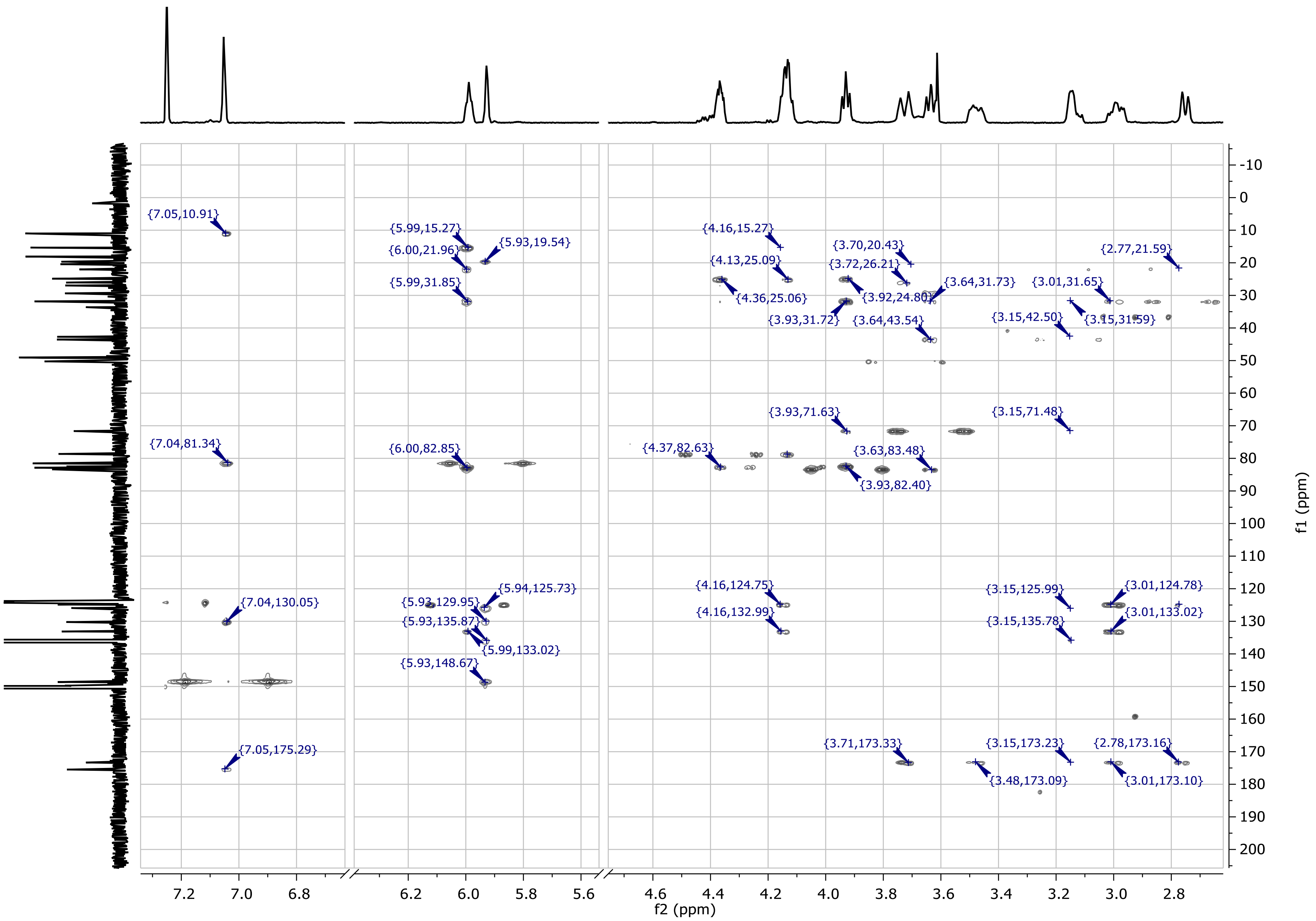

Figure S20: Slice 1 of 2D IMPACT-HMBC spectra of 16-Desmethylgymnodimine D (600 MHz Proton frequency, pyridine-d$_5$).

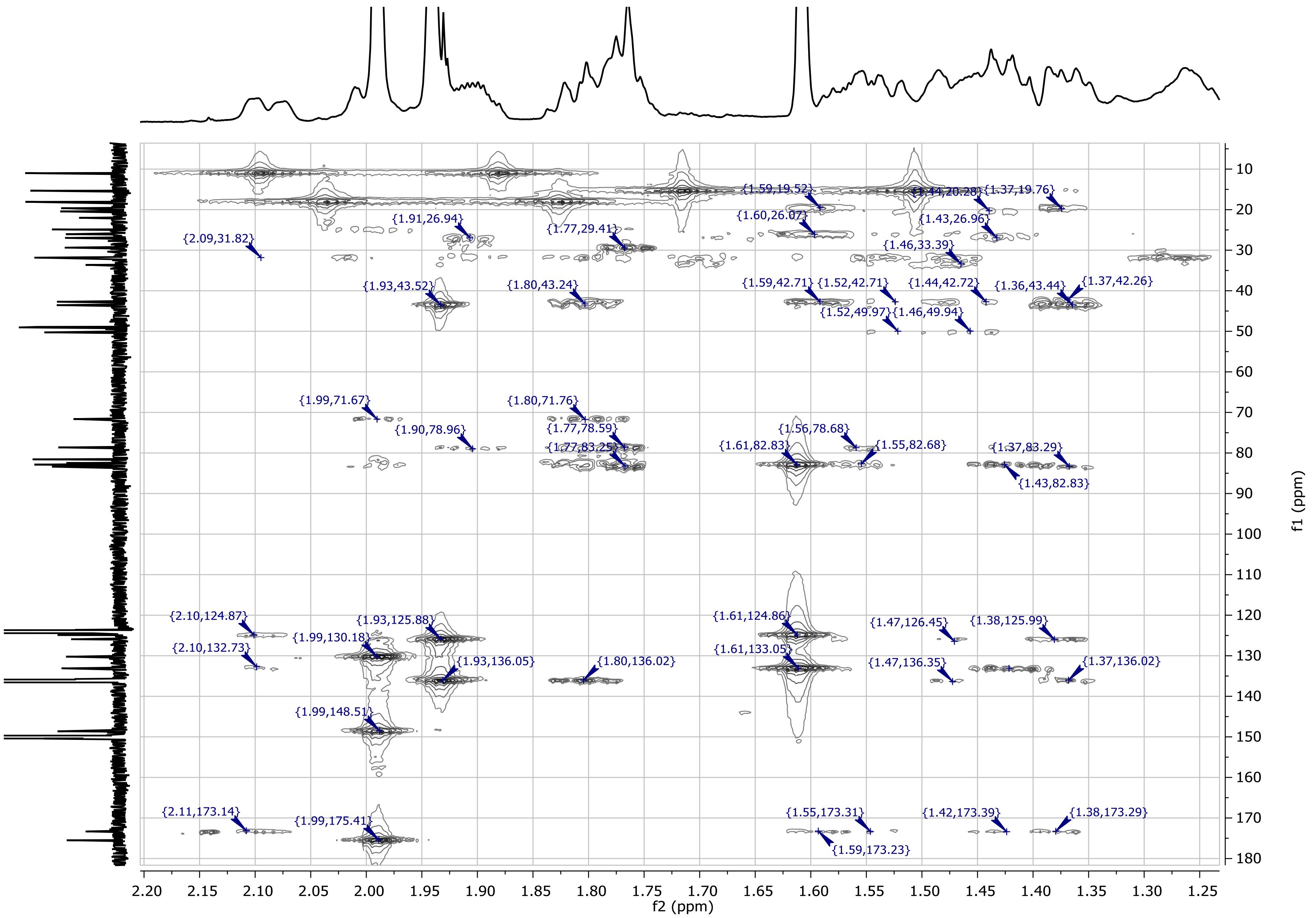

Figure S21: Slice 2 of 2D IMPACT-HMBC spectra of 16-Desmethylgymnodimine D (600 MHz Proton frequency, pyridine-d$_5$).

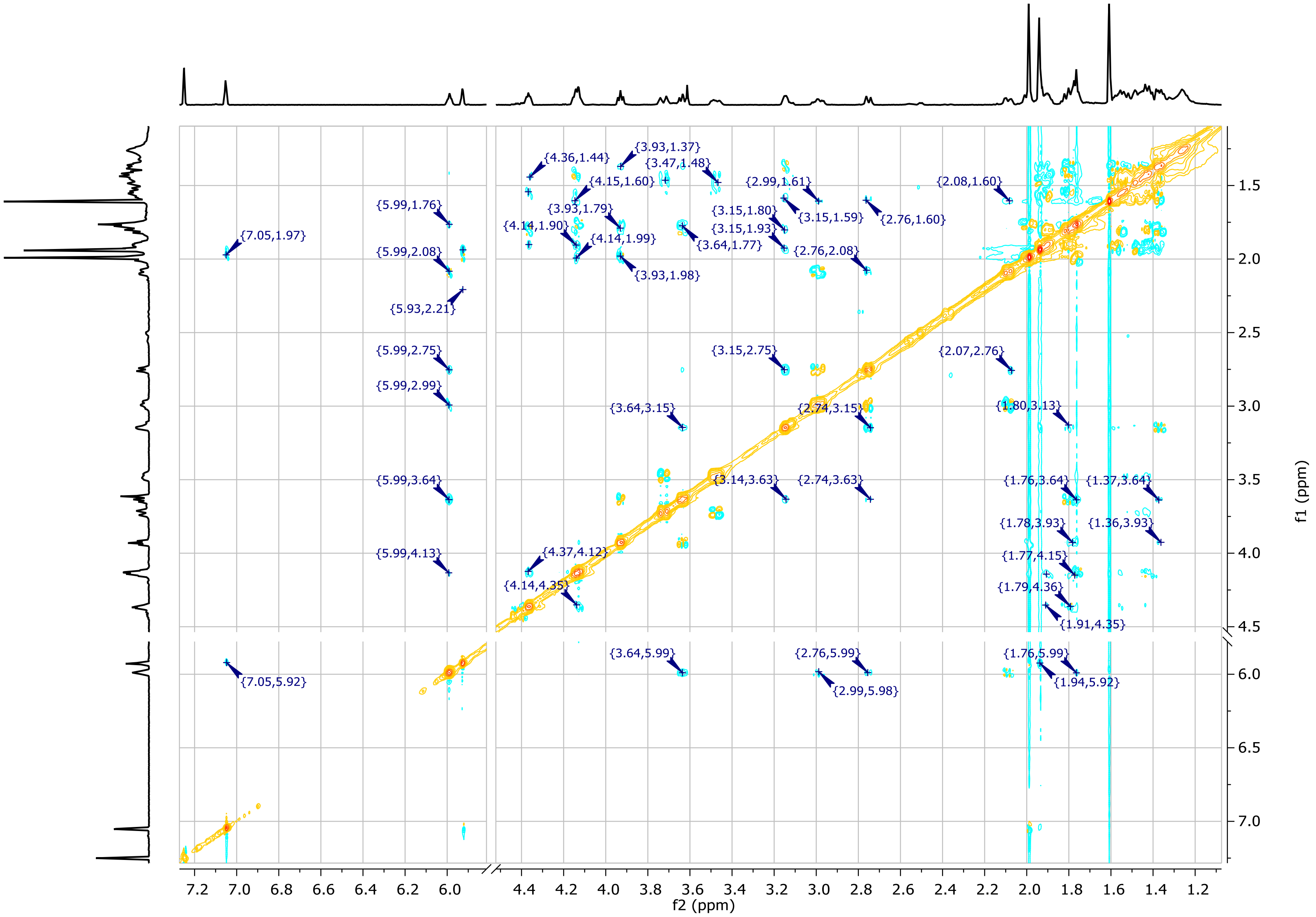

Figure S22: ROESY spectra of 16-Desmethylgymnodimine D (600 MHz Proton frequency, pyridine-d$_5$).

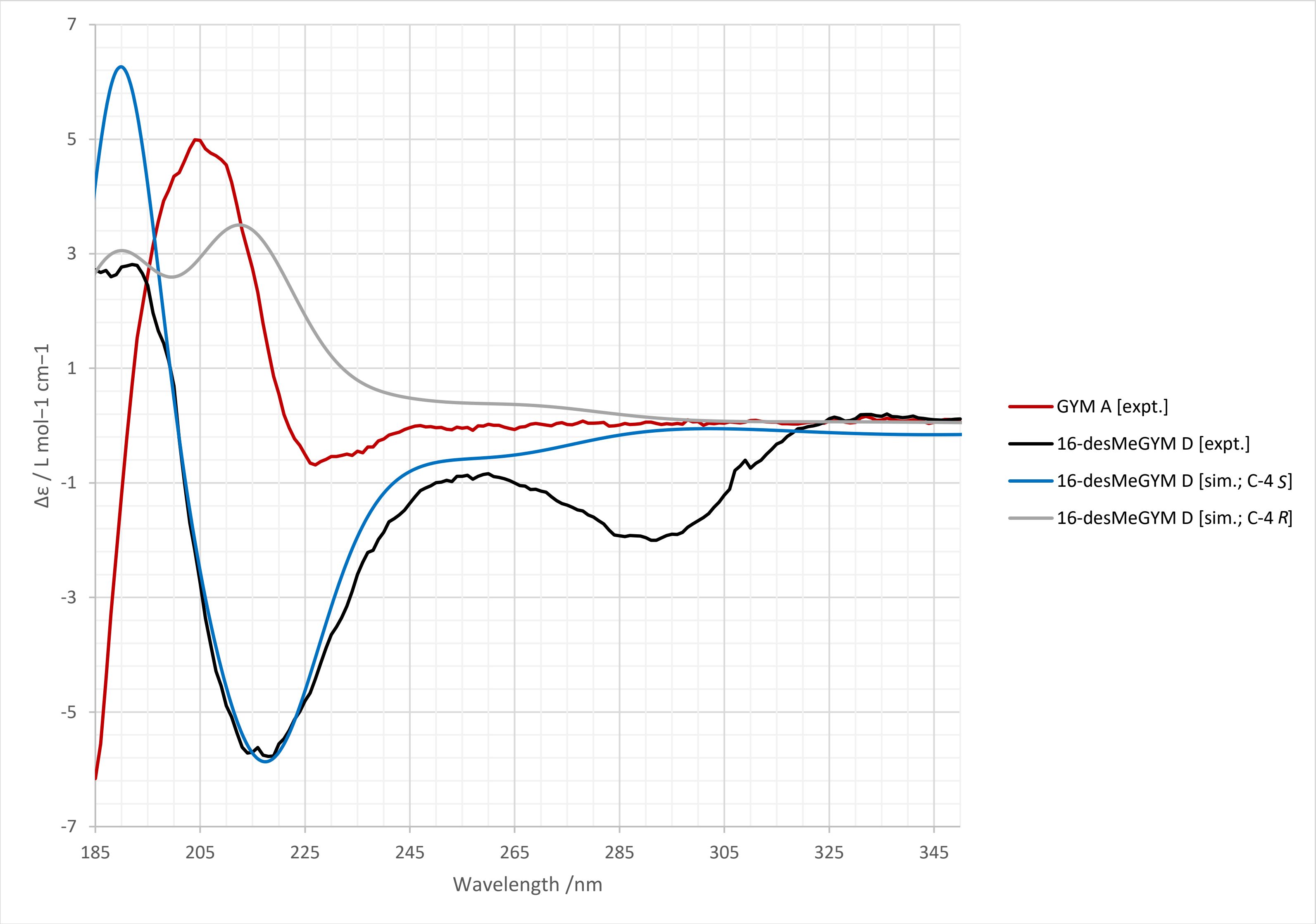

Figure S23: Measured (black) and simulated CD-spectra of 16-desmethyl GYM D (1)with *R* (grey) and *S* (blue) configuration at C-4 in comparison to the measured CD spectrum for GYM A (red).

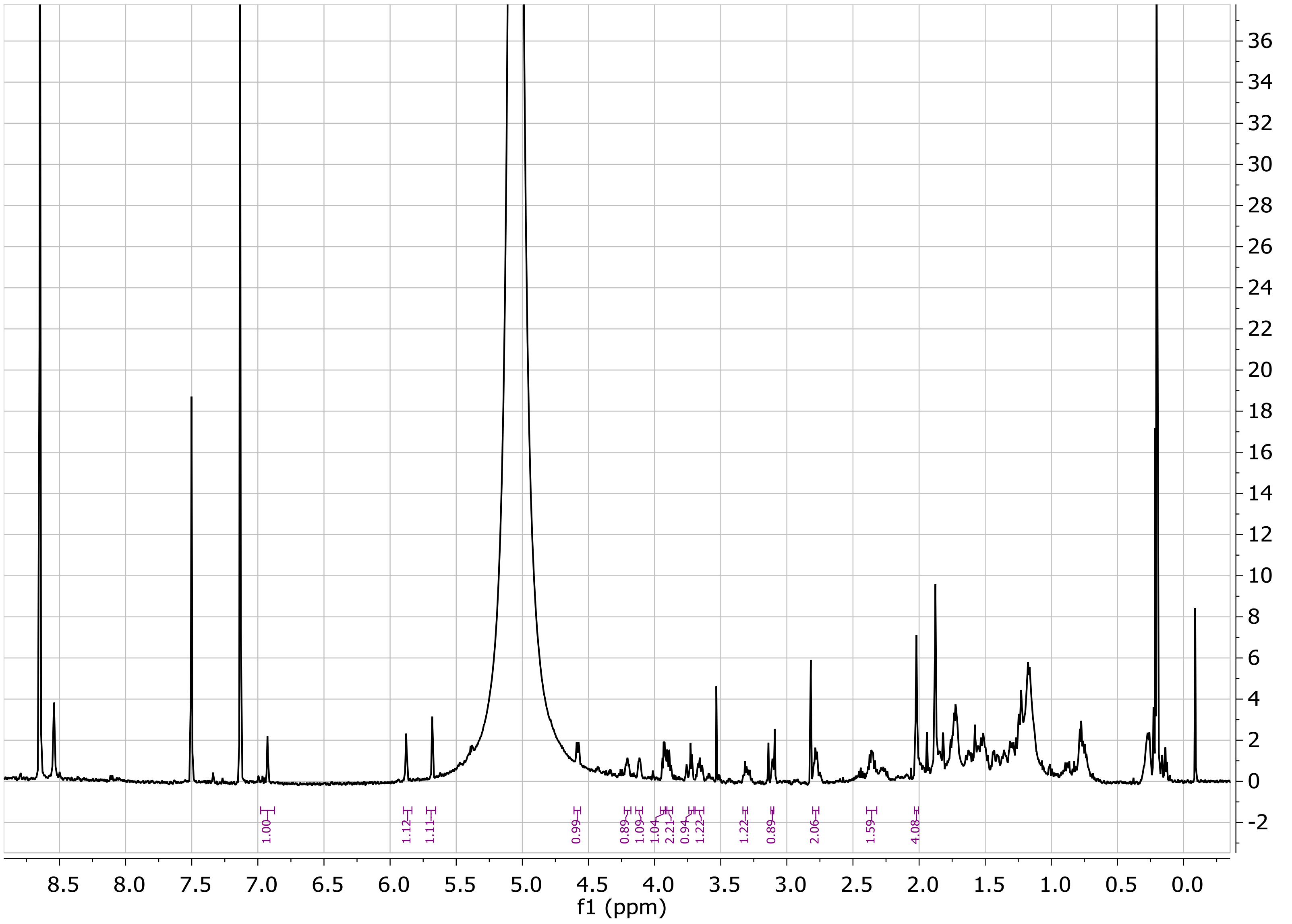

Figure S24: 1D Proton spectra of Gymnodimine E (600 MHz, pyridine-d_5).

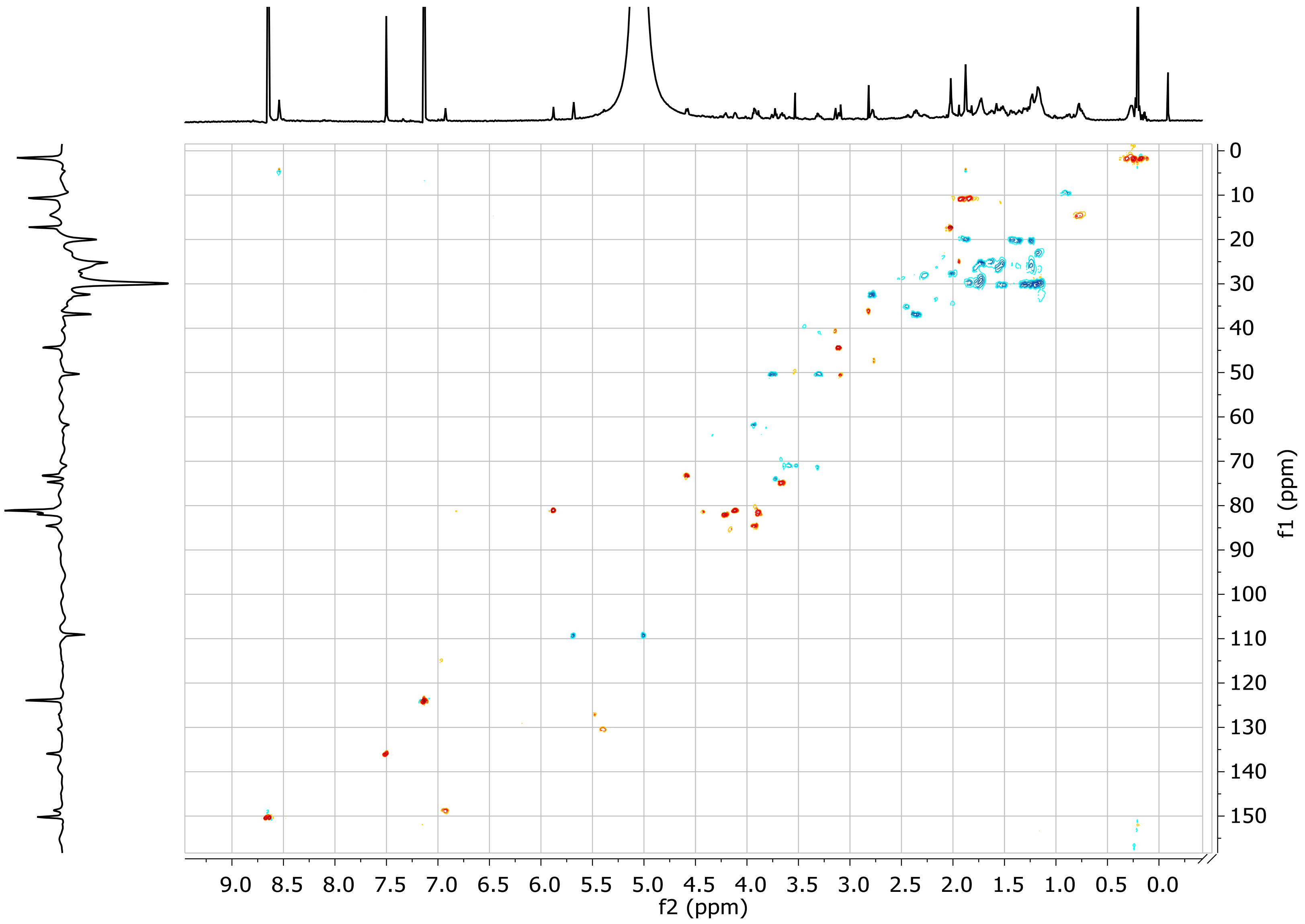

Figure S25: Multiplicity-edited 2D HSQC spectra of Gymnodimine E (600 MHz Proton frequency, pyridine-d_5).

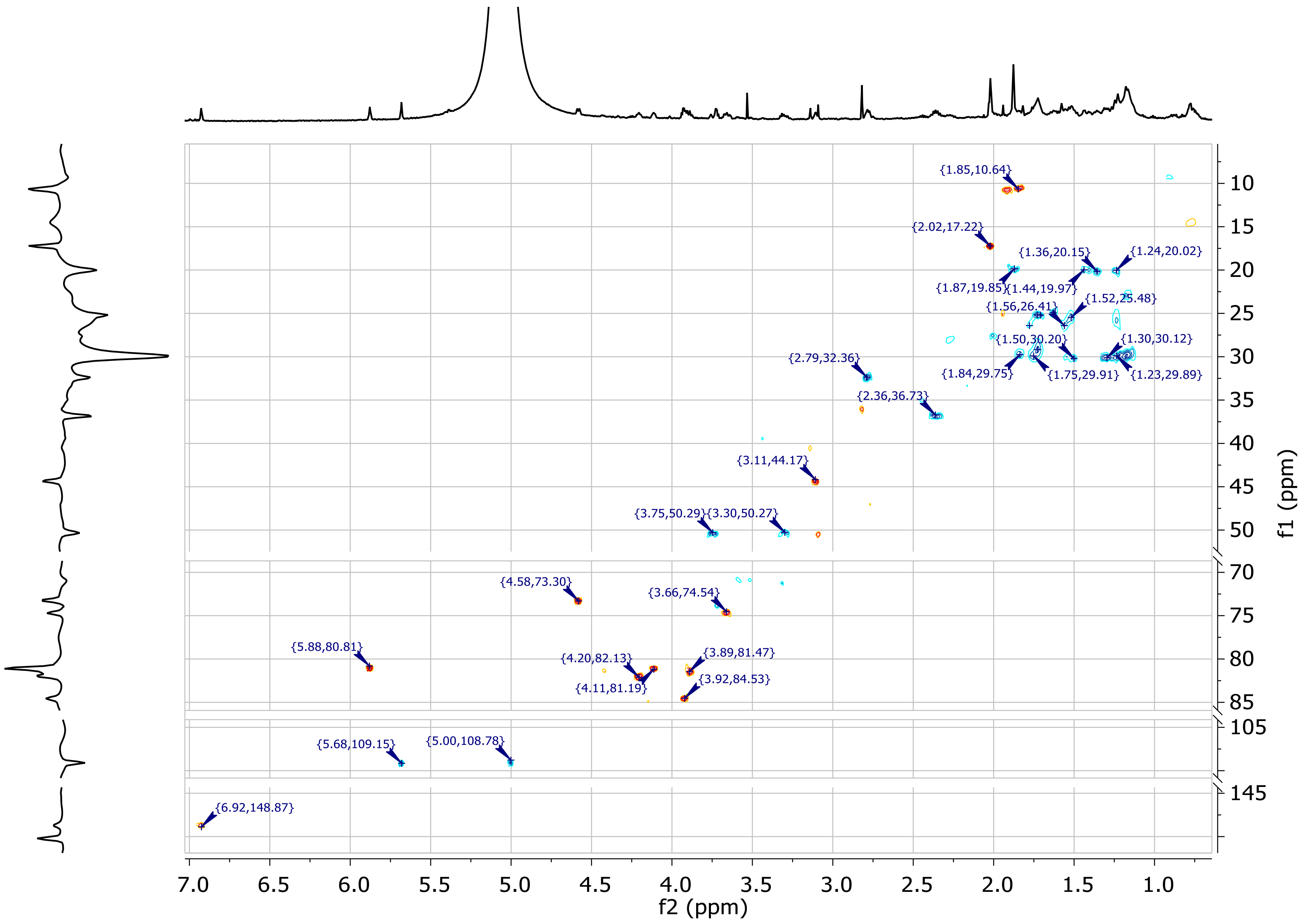

Figure S26: Slice 1 of Multiplicity-edited 2D HSQC spectra of Gymnodimine E (600 MHz Proton frequency, pyridine-d$_5$).

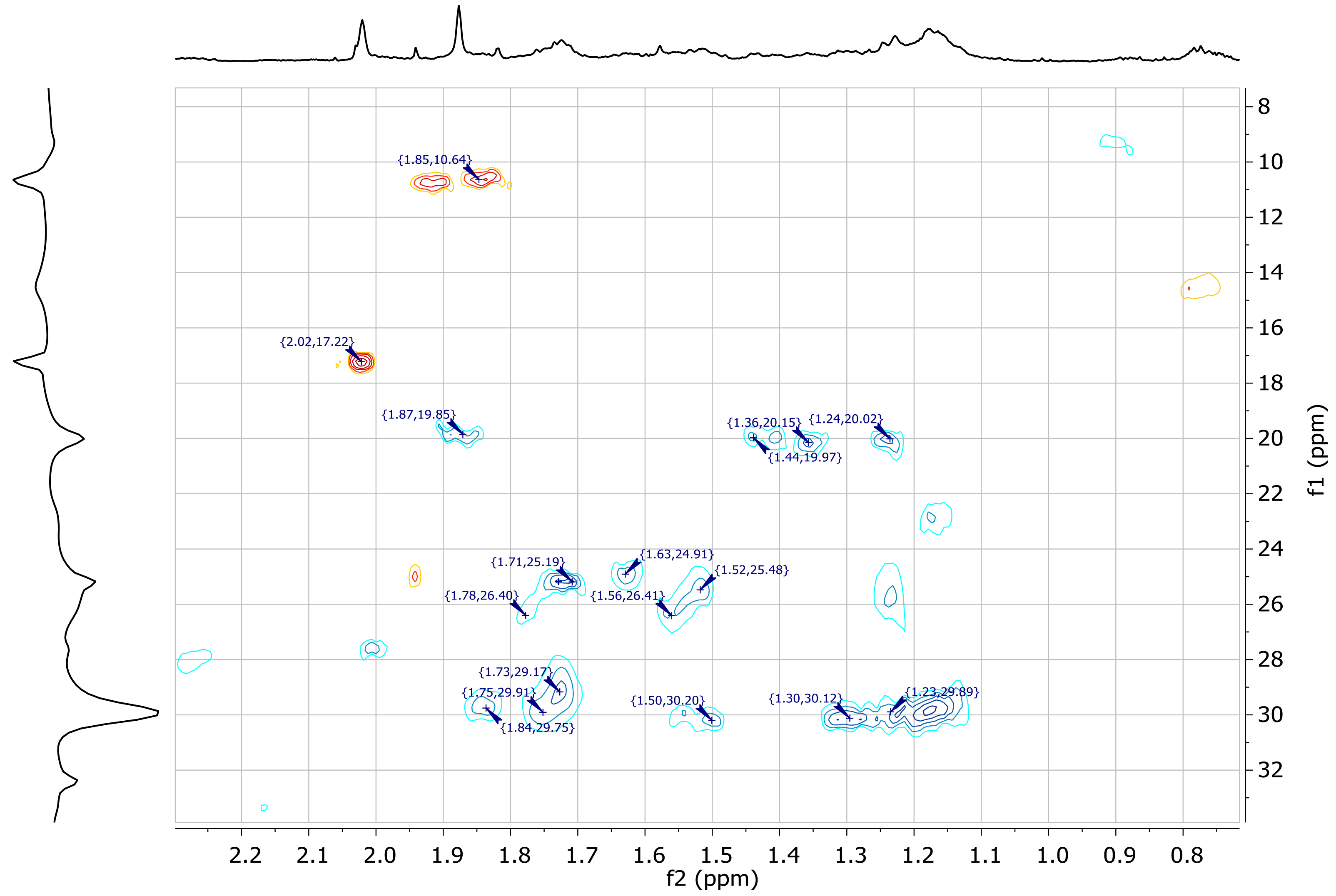

Figure S27: Slice 2 of Multiplicity-edited 2D HSQC spectra of Gymnodimine E (600 MHz Proton frequency, pyridine-d$_5$).

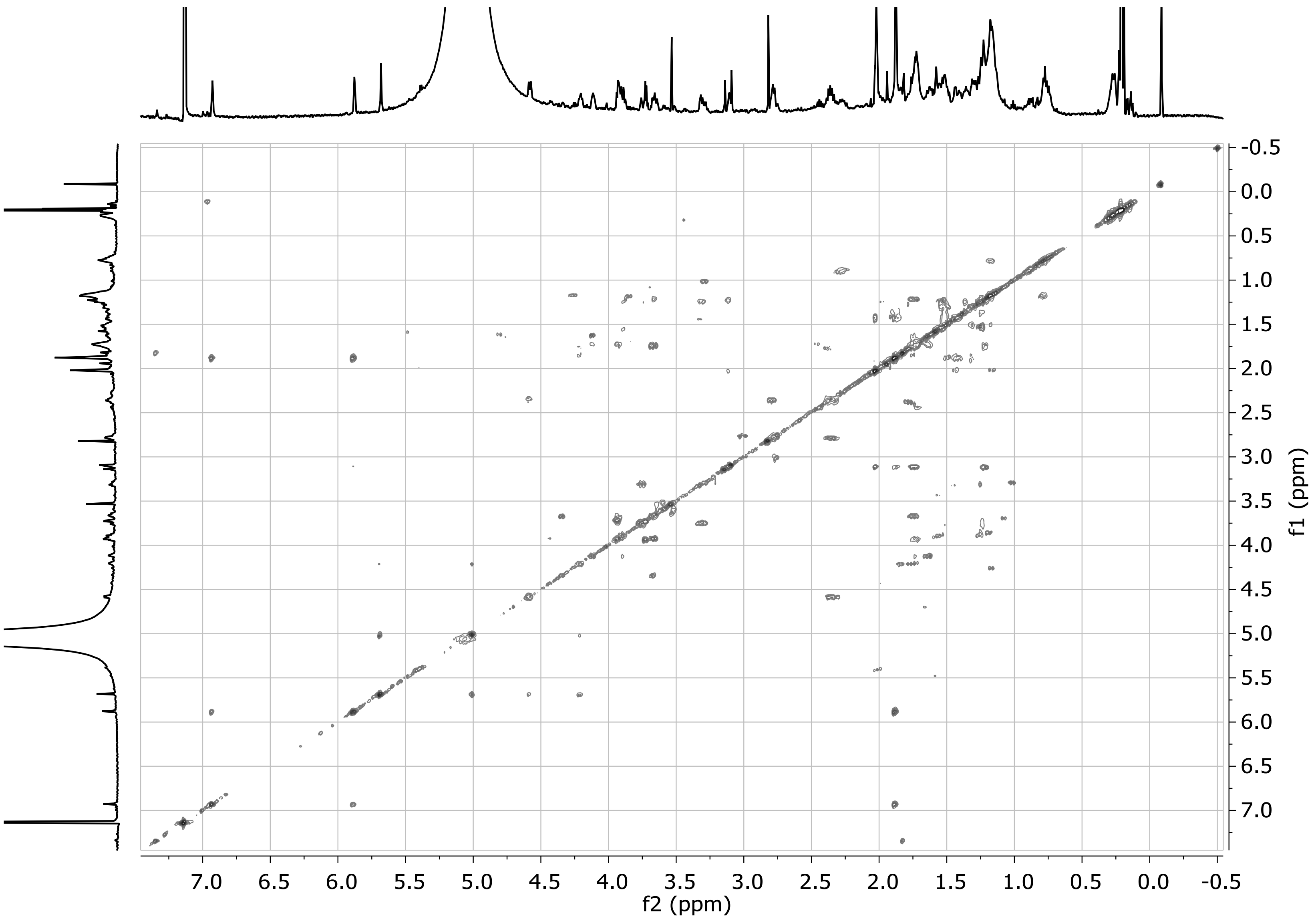

Figure S28: 2D COSY spectra of Gymnodimine E (600 MHz, pyridine-d_5).

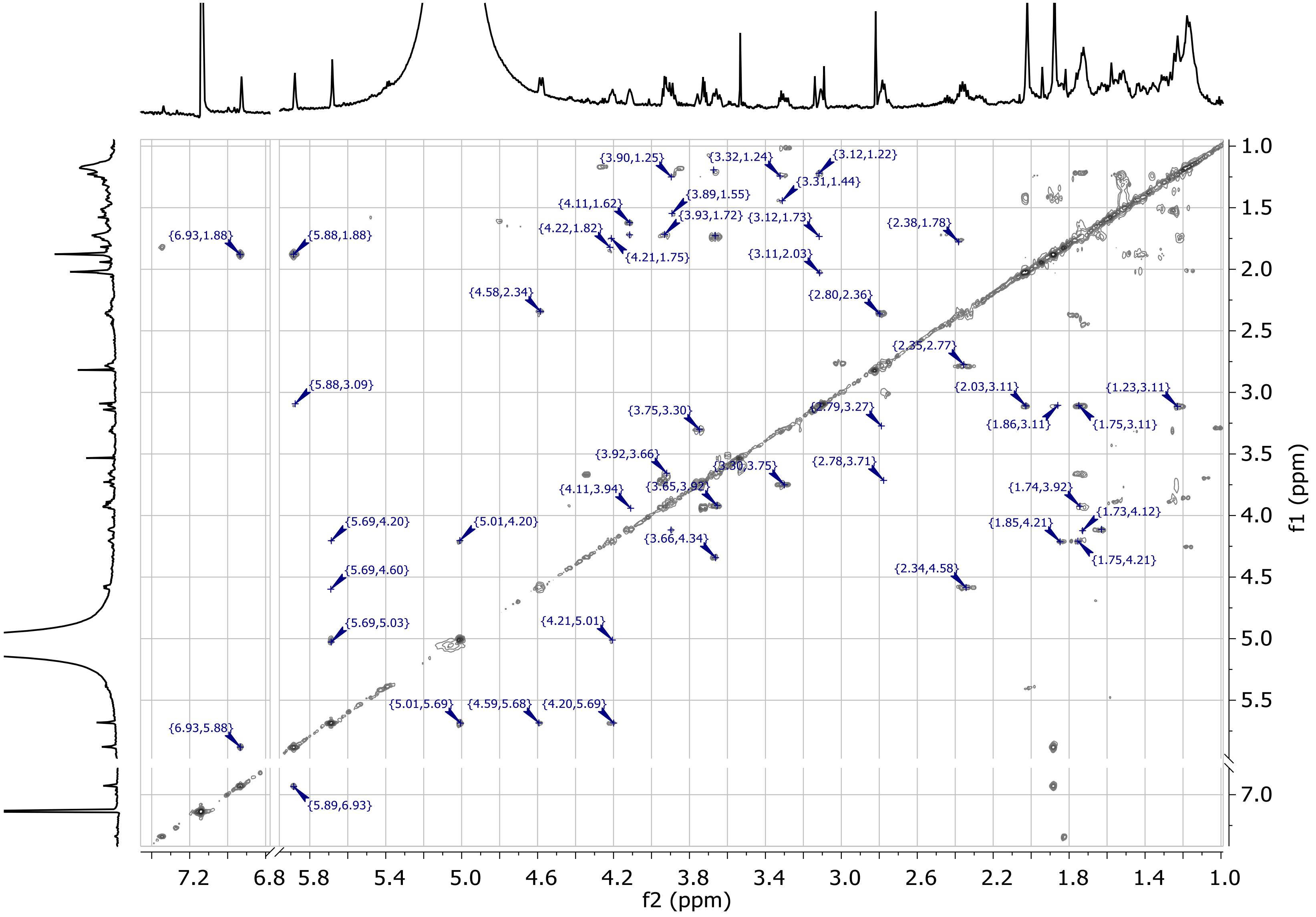

Figure S29: Slice of COSY spectra of Gymnodimine E (600 MHz Proton frequency, pyridine-d$_5$).

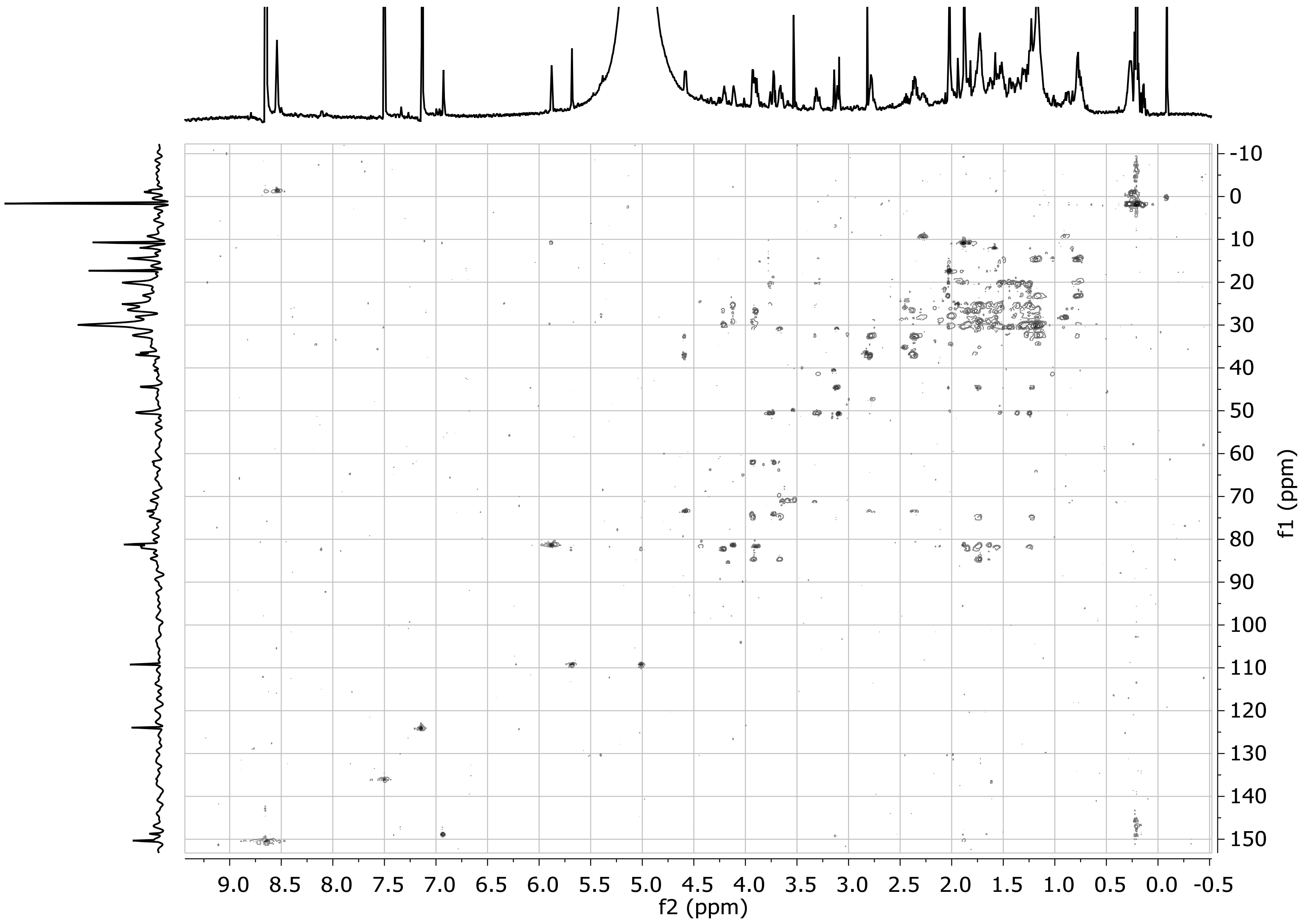

Figure S30: 2D HSQC-TOCSY spectra of Gymnodimine E (600 MHz Proton frequency, pyridine-d5).

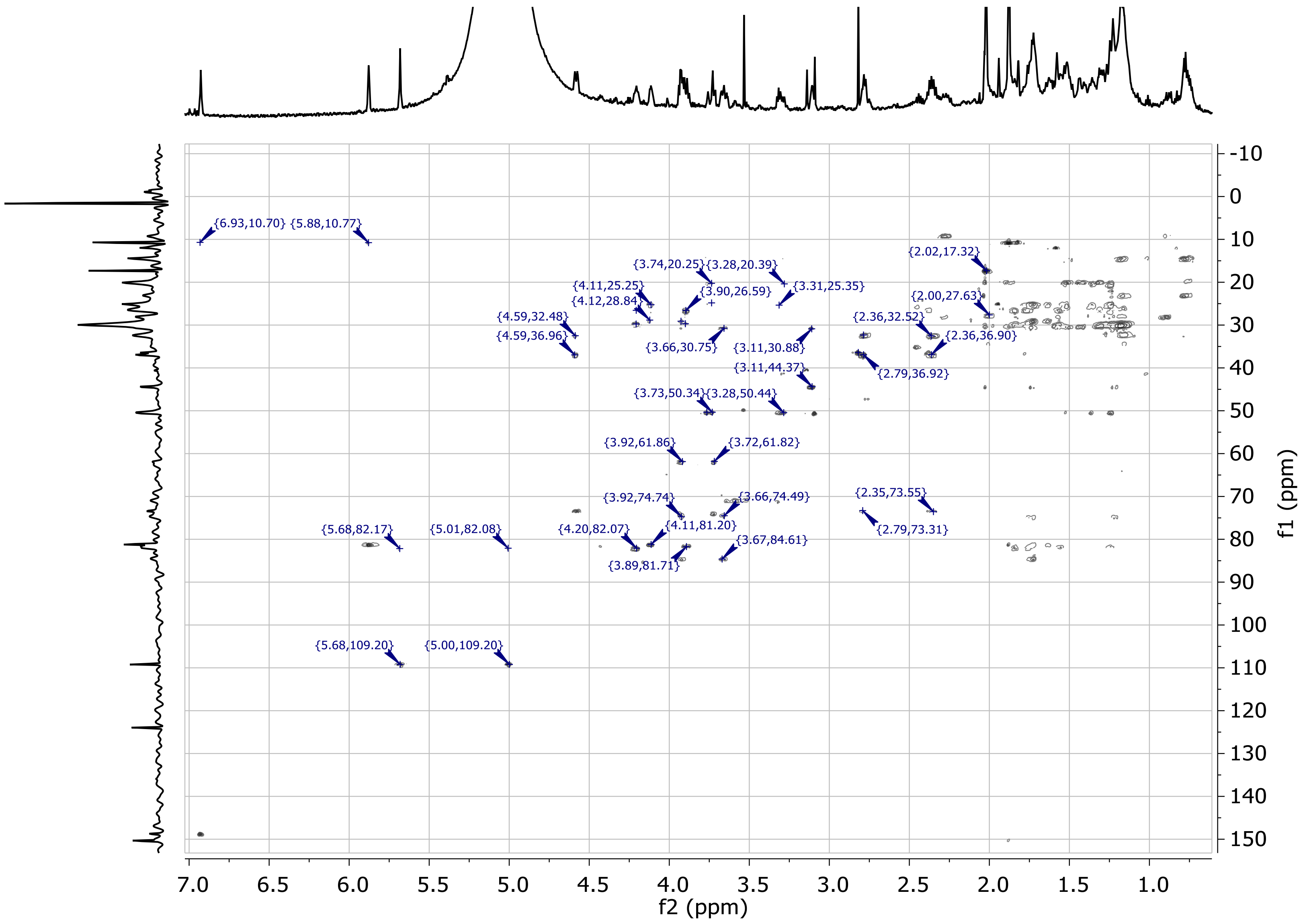

Figure S31: Slice of 2D HSQC-TOCSY spectra of Gymnodimine E (600 MHz Proton frequency, pyridine-d$_5$).

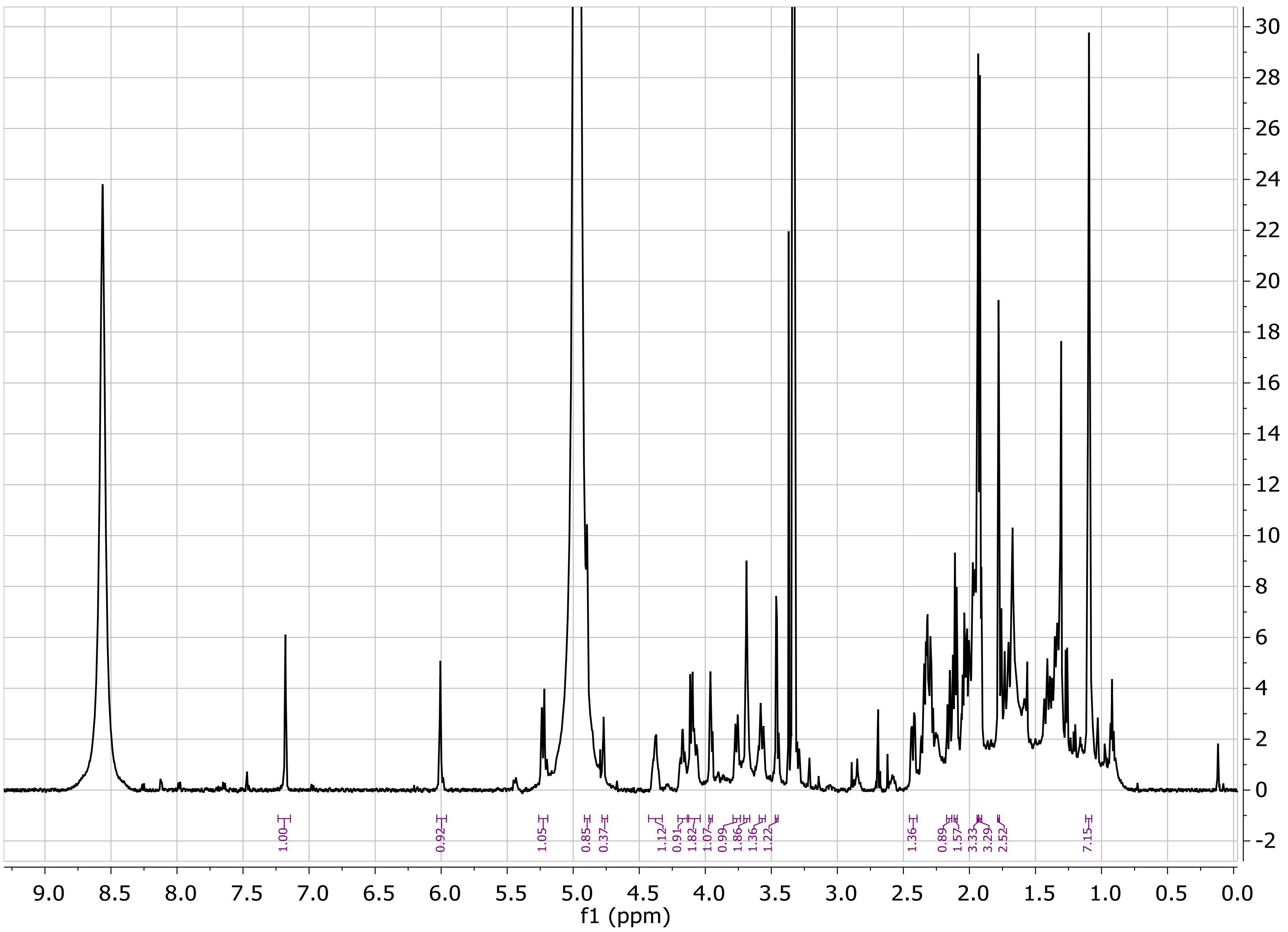

Figure S32: 1D Proton spectra of 20-Hydroxy-13,19-didesMethyl-SPX C (600 MHz, CD_3OD).

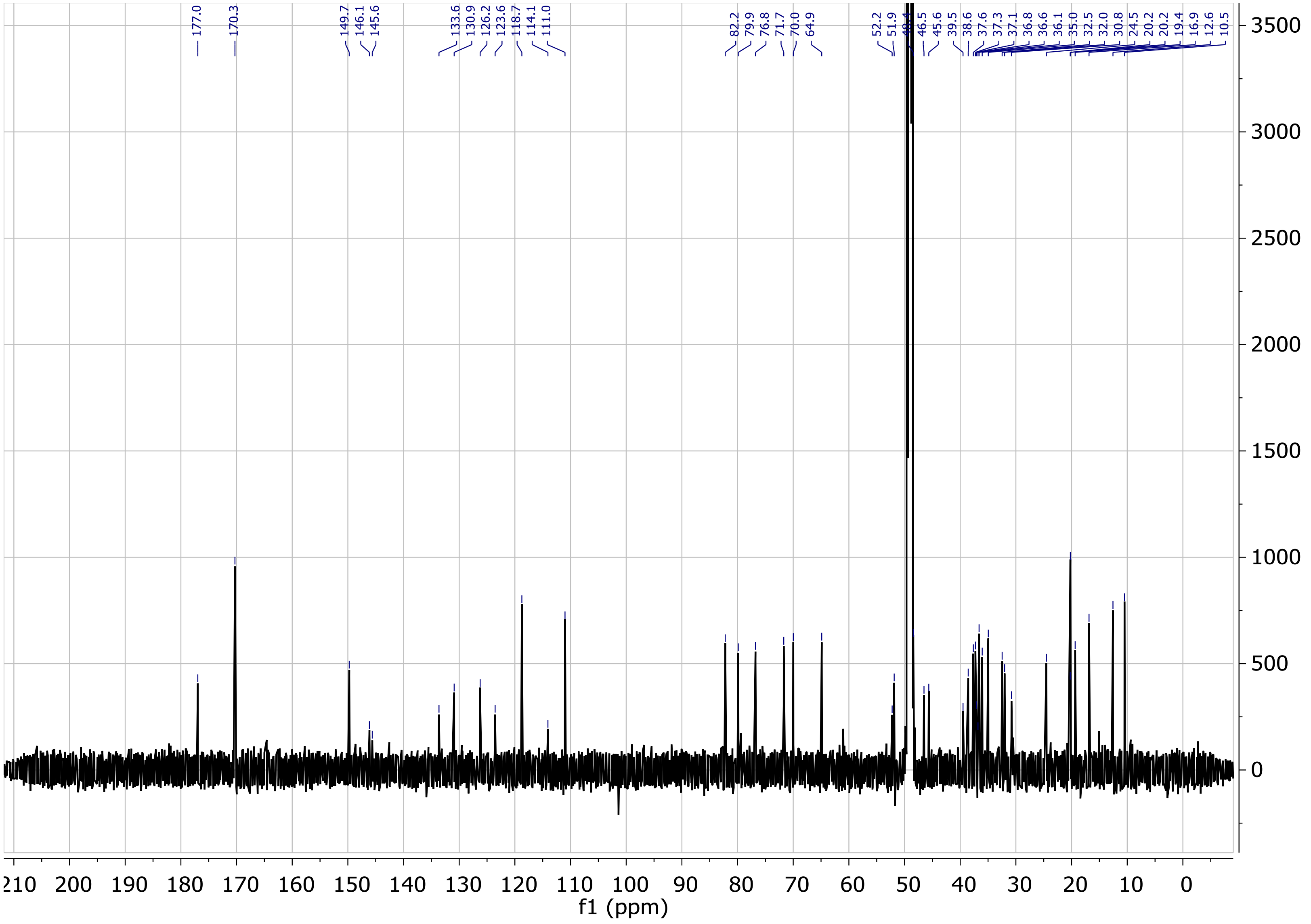

Figure S33: 1D ^{13}C-spectra of 20-Hydroxy-13,19-didesMethyl-SPX C (150 MHz, CD_3OD).

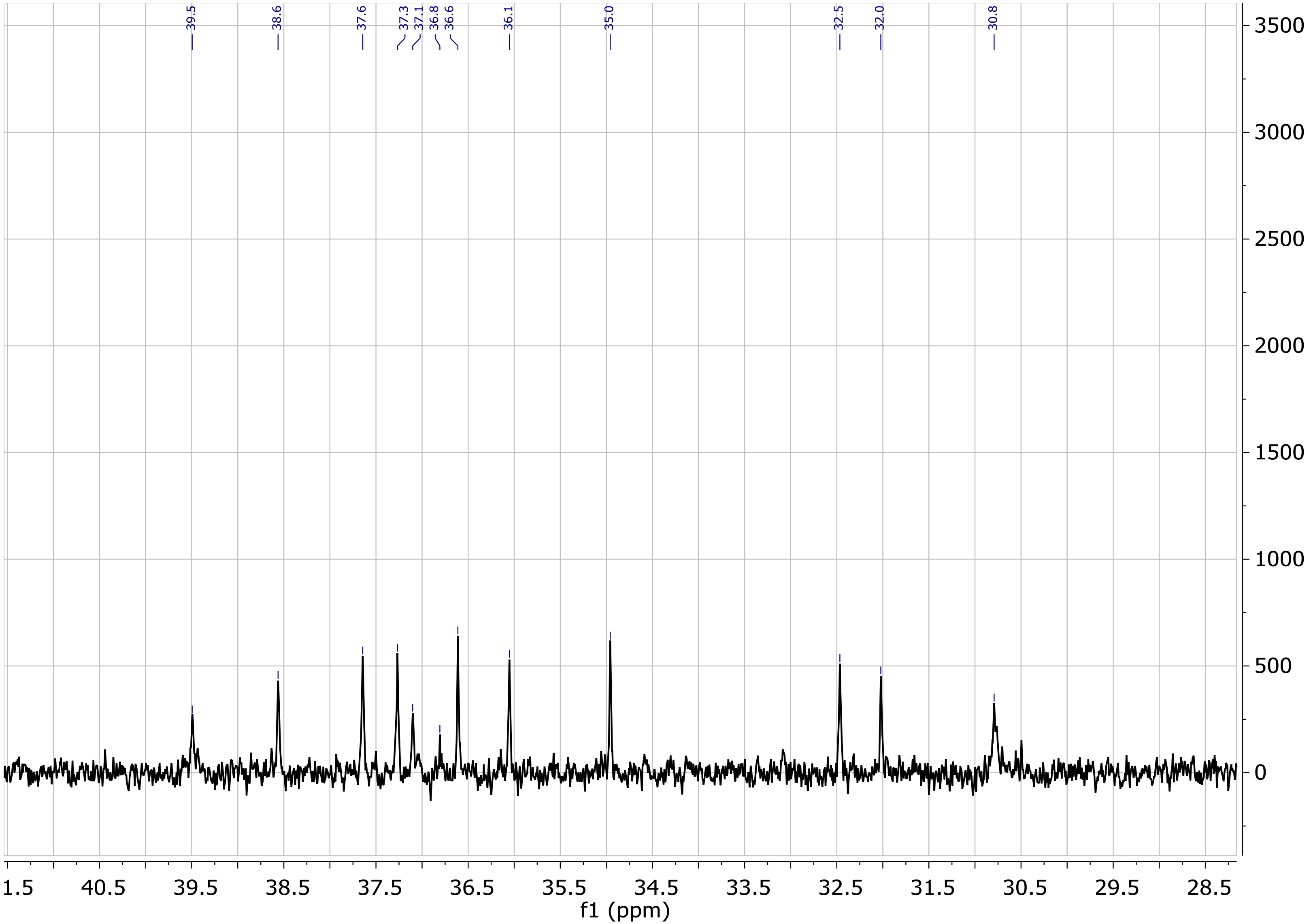

Figure S34: Slice of 1D ^{13}C-spectra of 20-Hydroxy-13,19-didesMethyl-SPX C (150 MHz, CD_3OD).

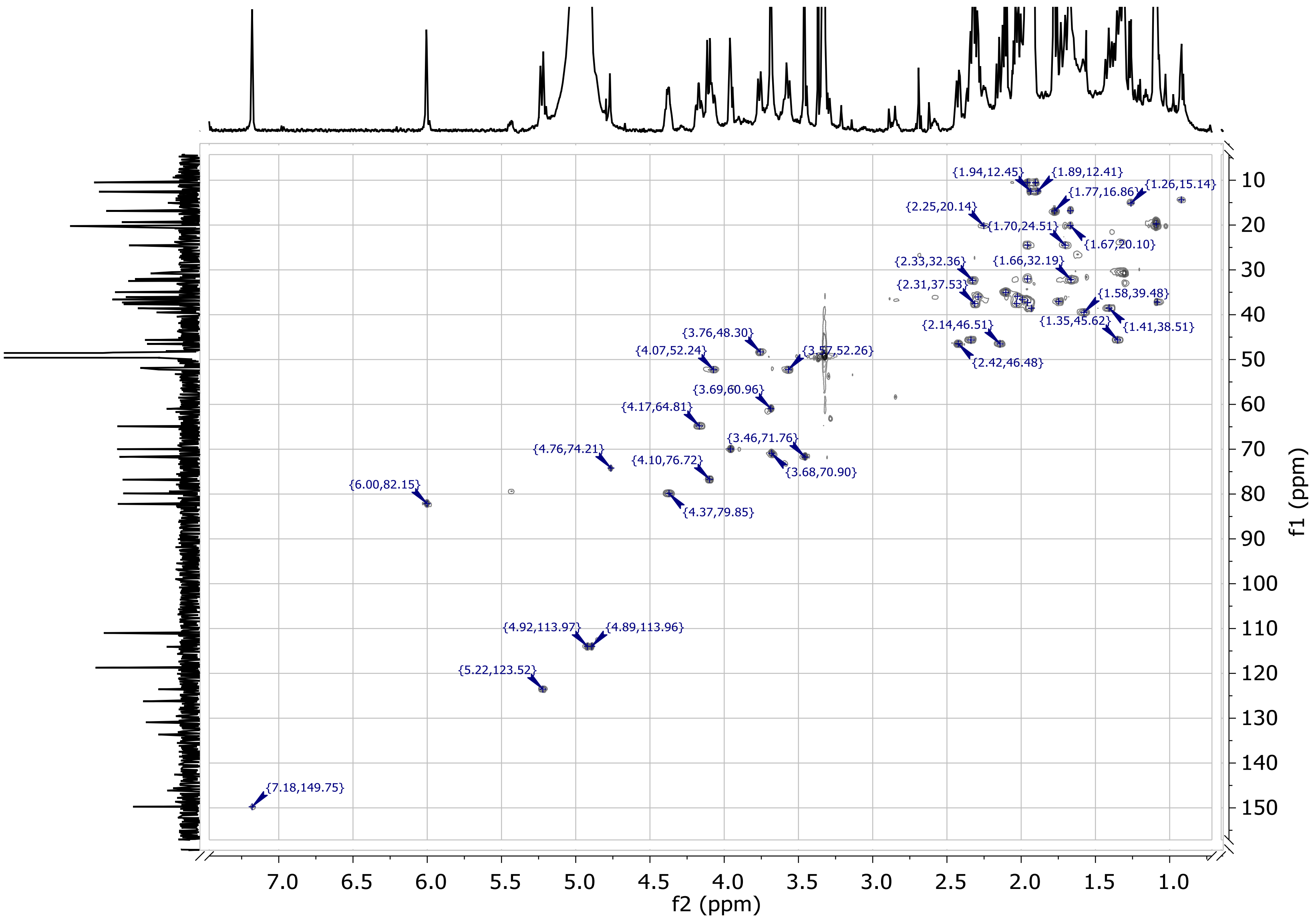

Figure S35: 2D HSQC spectra of 20-Hydroxy-13,19-didesMethyl-SPX C (600 MHz Proton frequency, CD3OD).

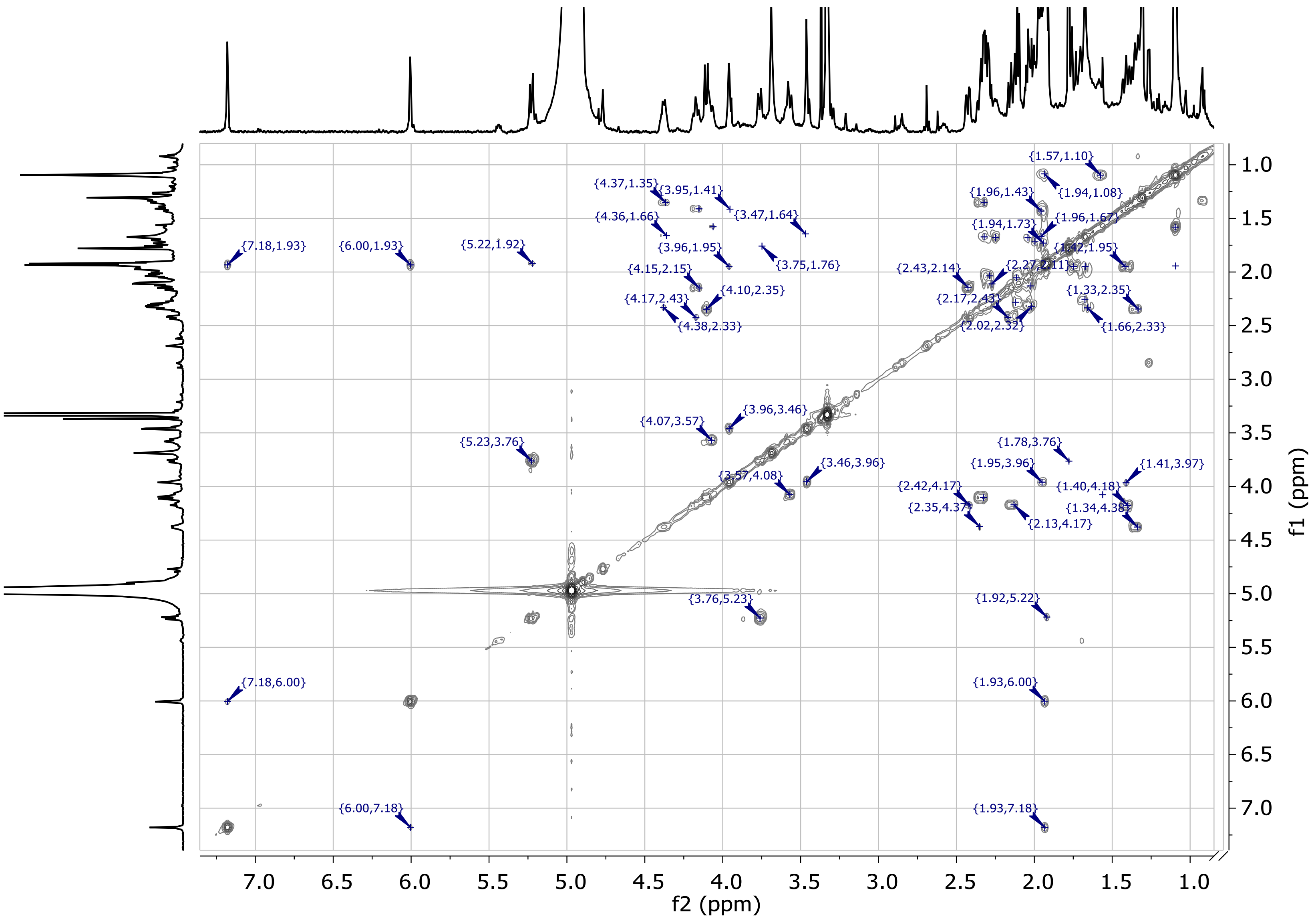

Figure S36: COSY spectra of 20-Hydroxy-13,19-didesMethyl-SPX C (600 MHz Proton frequency, CD_3OD).

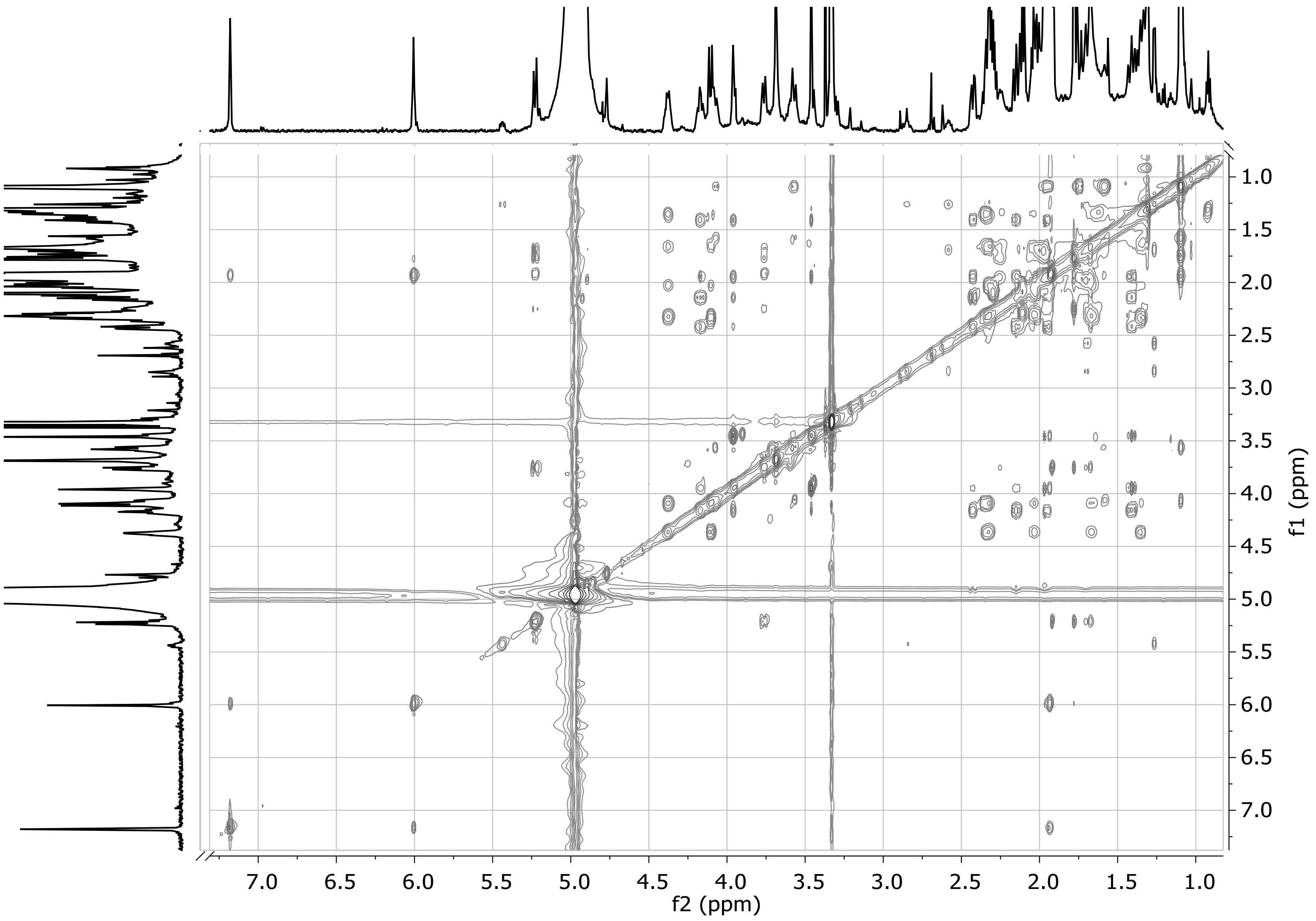

Figure S37: 2D TOCSY spectra of 20-Hydroxy-13,19-didesMethyl-SPX C (600 MHz, CD_3OD).

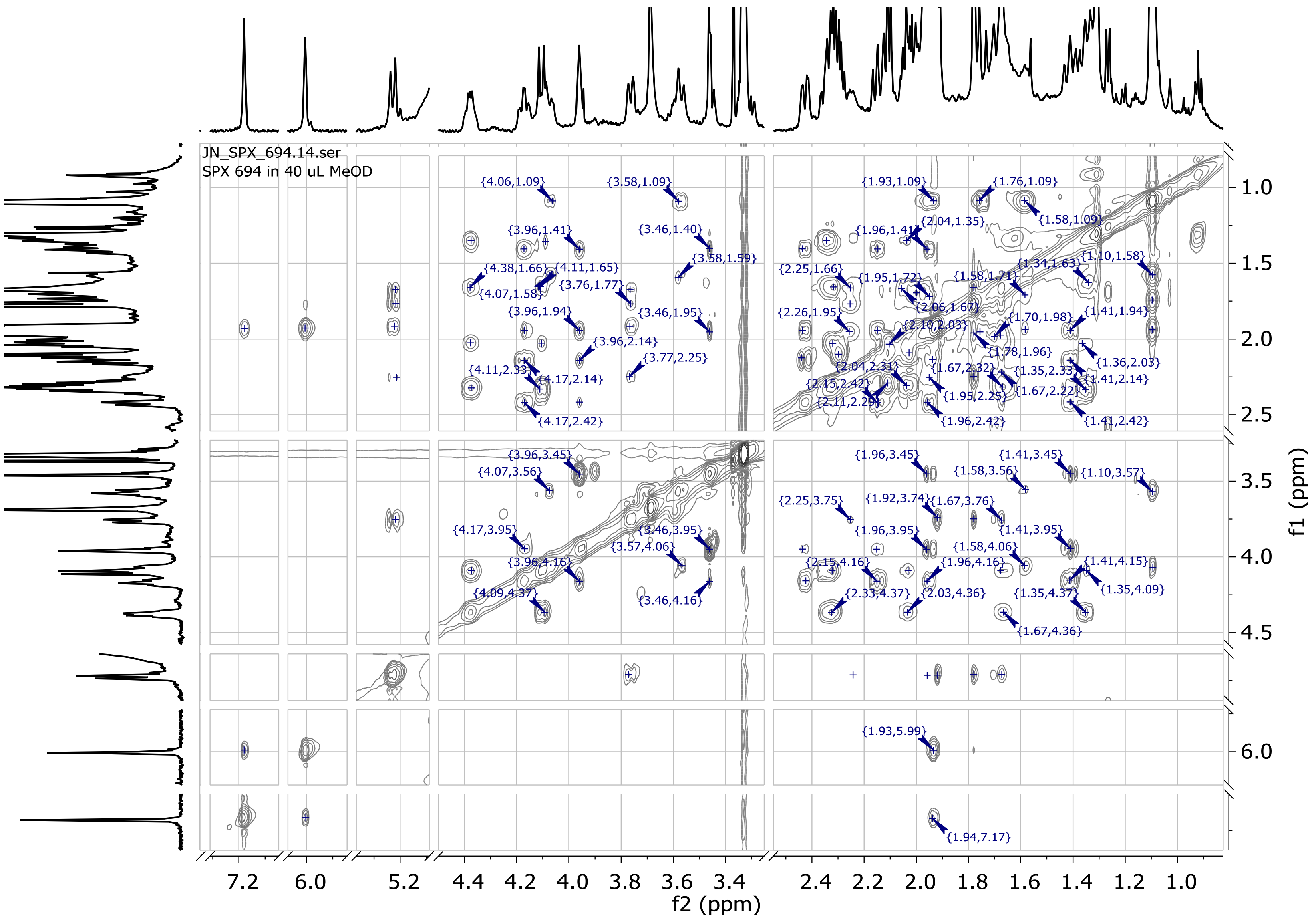

Figure S38: Slice of 2D TOCSY spectra of 20-Hydroxy-13,19-didesMethyl-SPX C (600 MHz, CD_3OD).

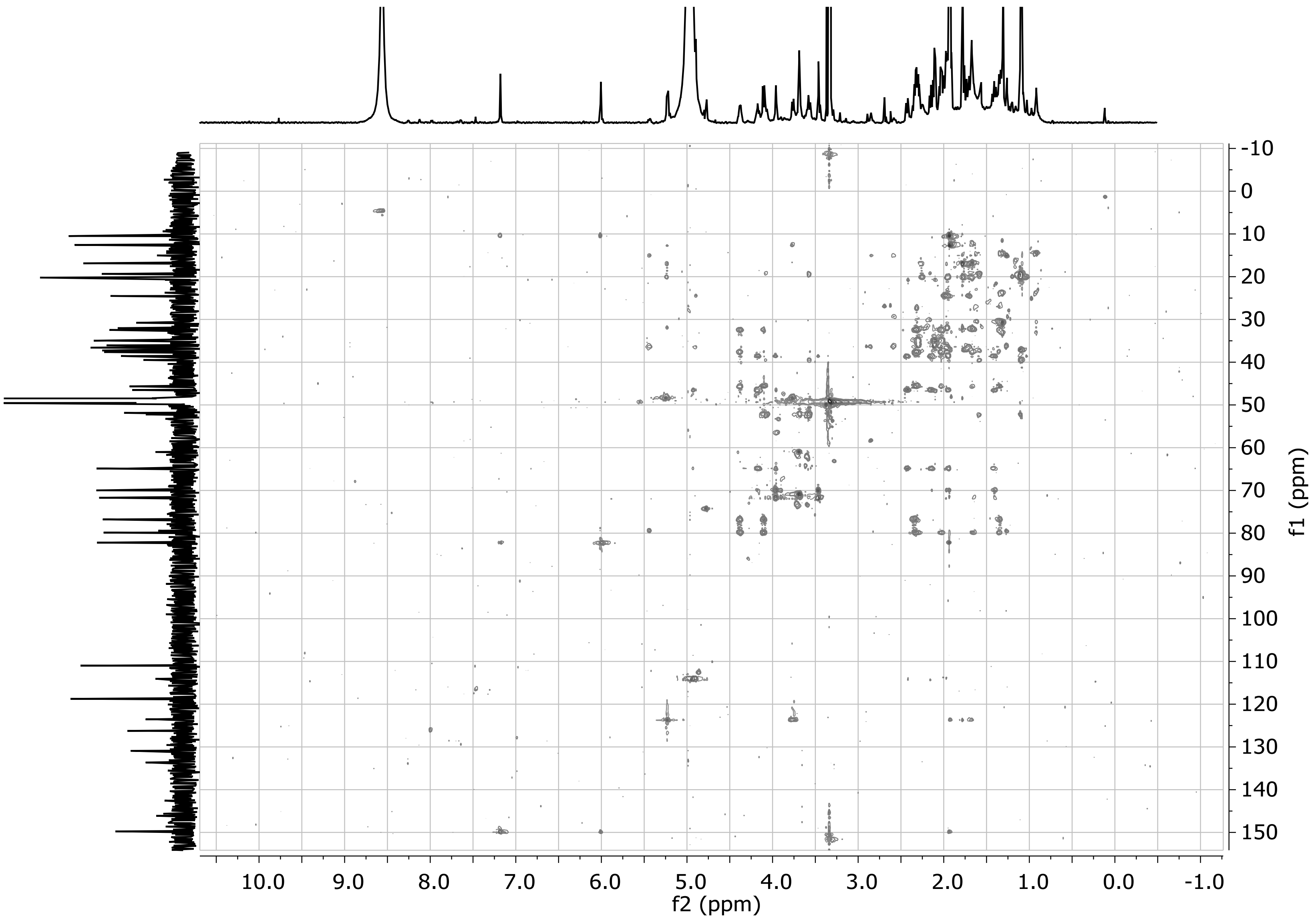

Figure S39: HSQC-TOCSY spectra of 20-Hydroxy-13,19-didesMethyl-SPX C (600 MHz Proton frequency, CD_3OD).

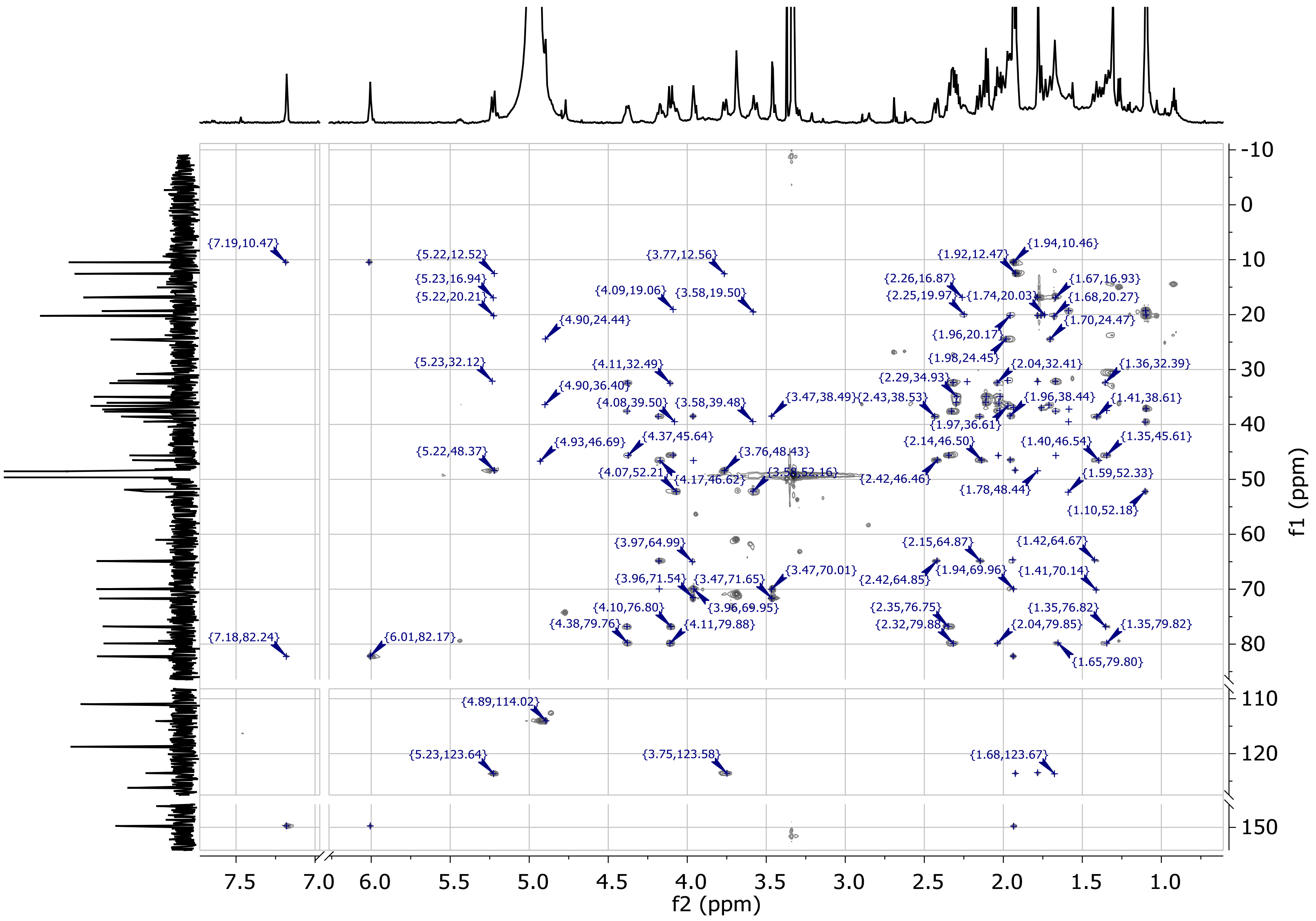

Figure S40: Slice of HSQC-TOCSY spectra of 20-Hydroxy-13,19-didesMethyl-SPX C (600 MHz Proton frequency, CD_3OD).

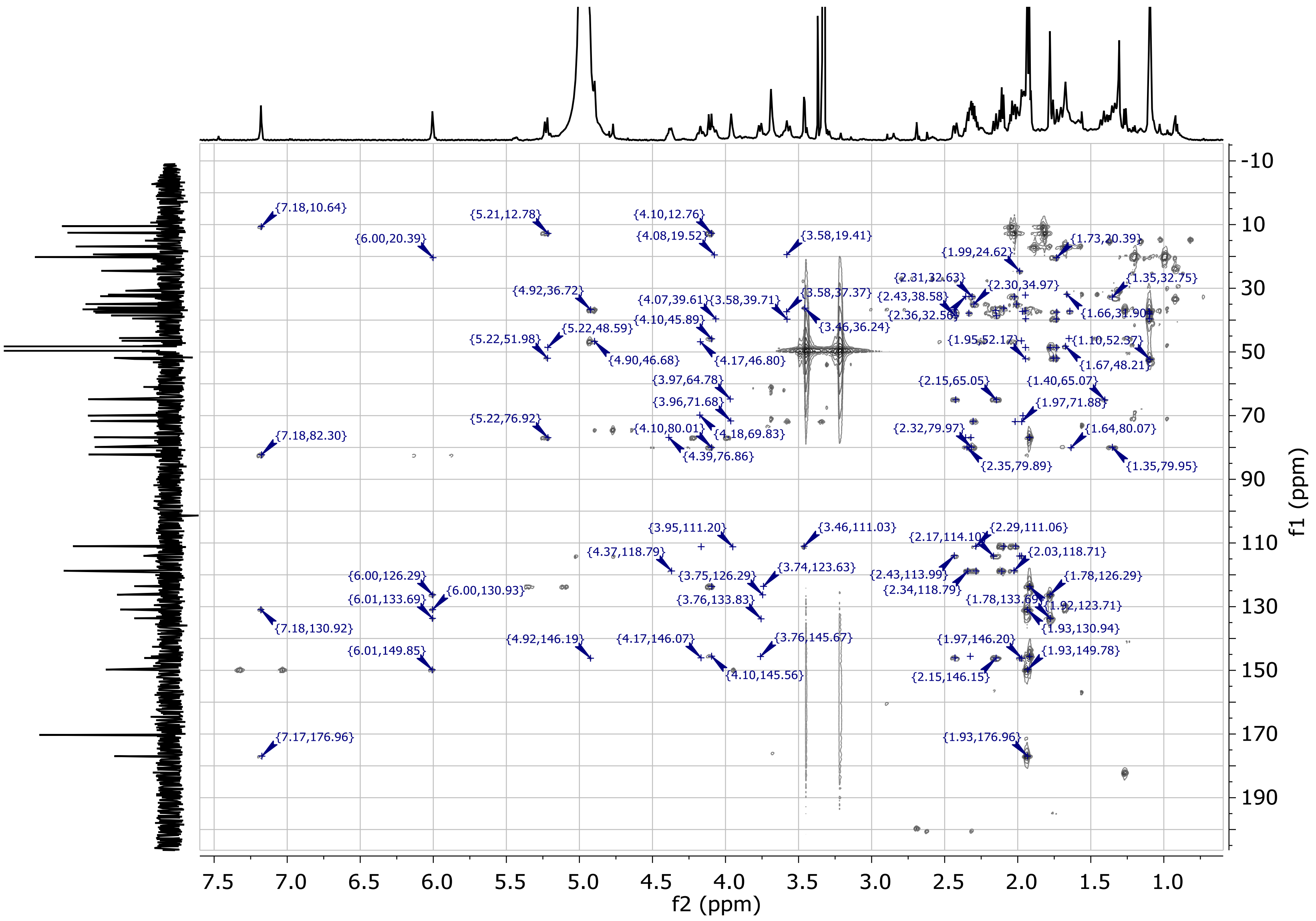

Figure S41: HMBC spectra of 20-Hydroxy-13,19-didesMethyl-SPX C (600 MHz Proton frequency, CD_3OD).

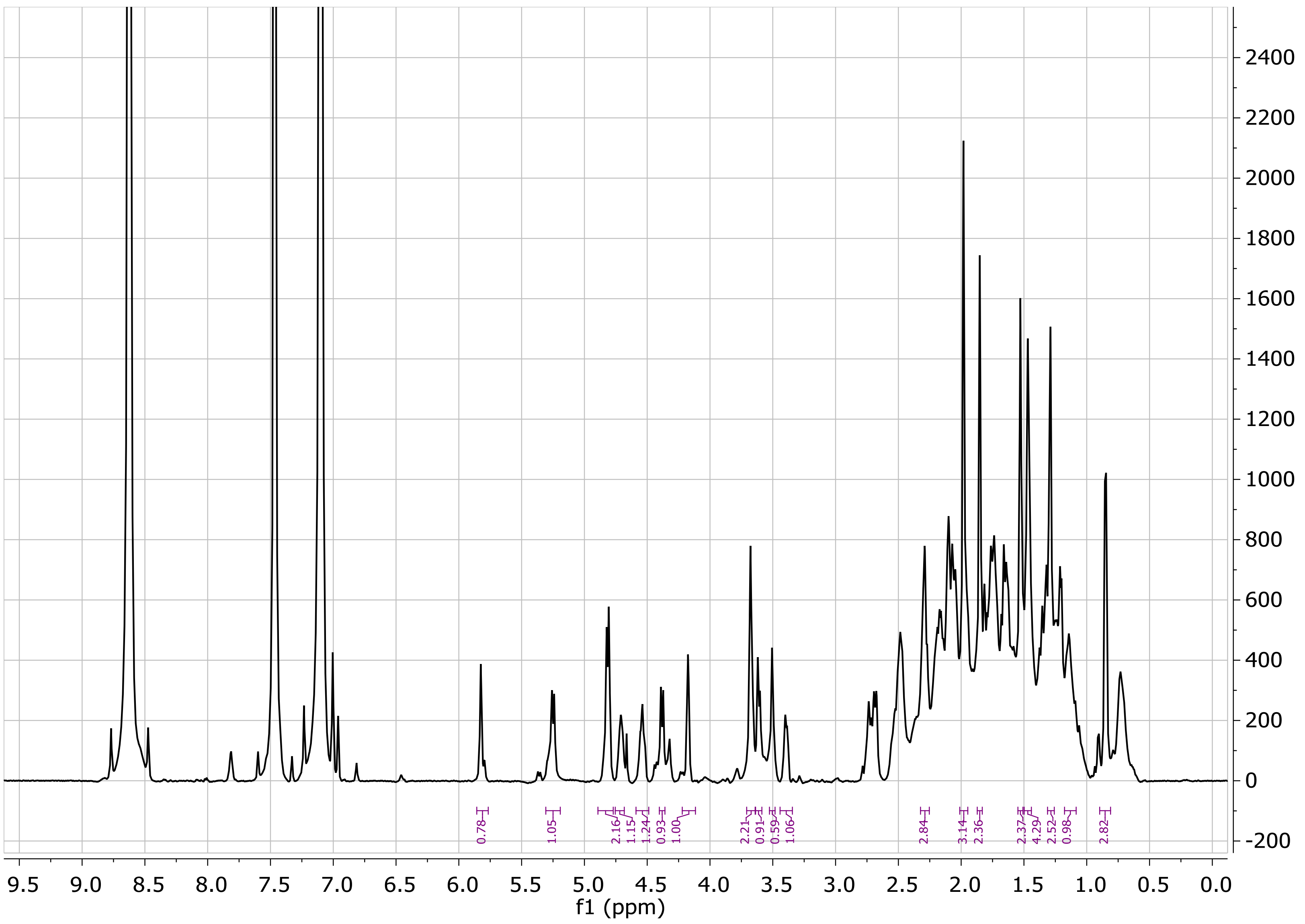

Figure S42: 1D Proton spectra of 20-Hydroxy-13,19-didesMethyl-SPX C (600 MHz, pyridine-d$_5$).

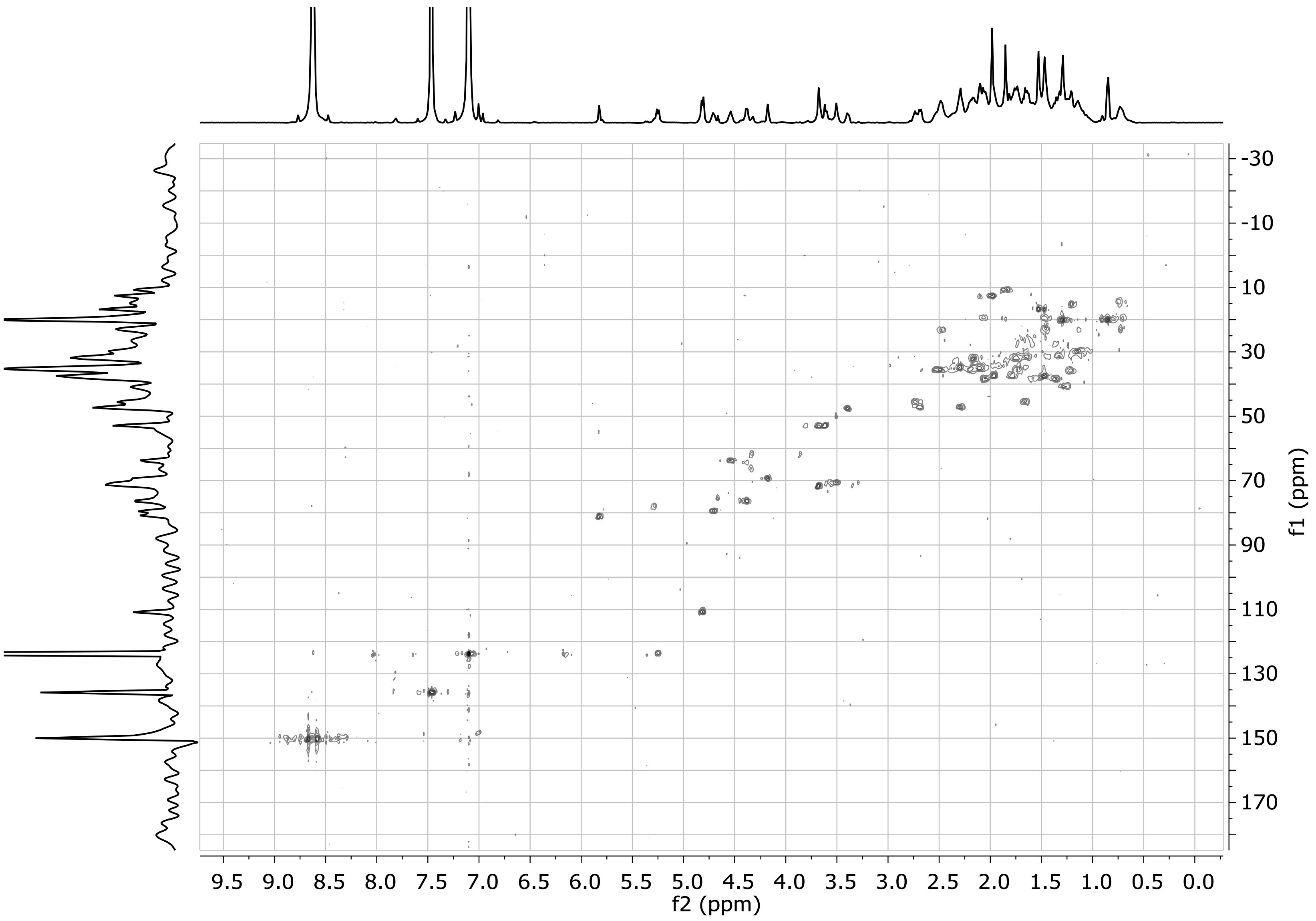

Figure S43: 2D HSQC spectra of 20-Hydroxy-13,19-didesMethyl-SPX C (600 MHz Proton frequency, pyridine-d_5).

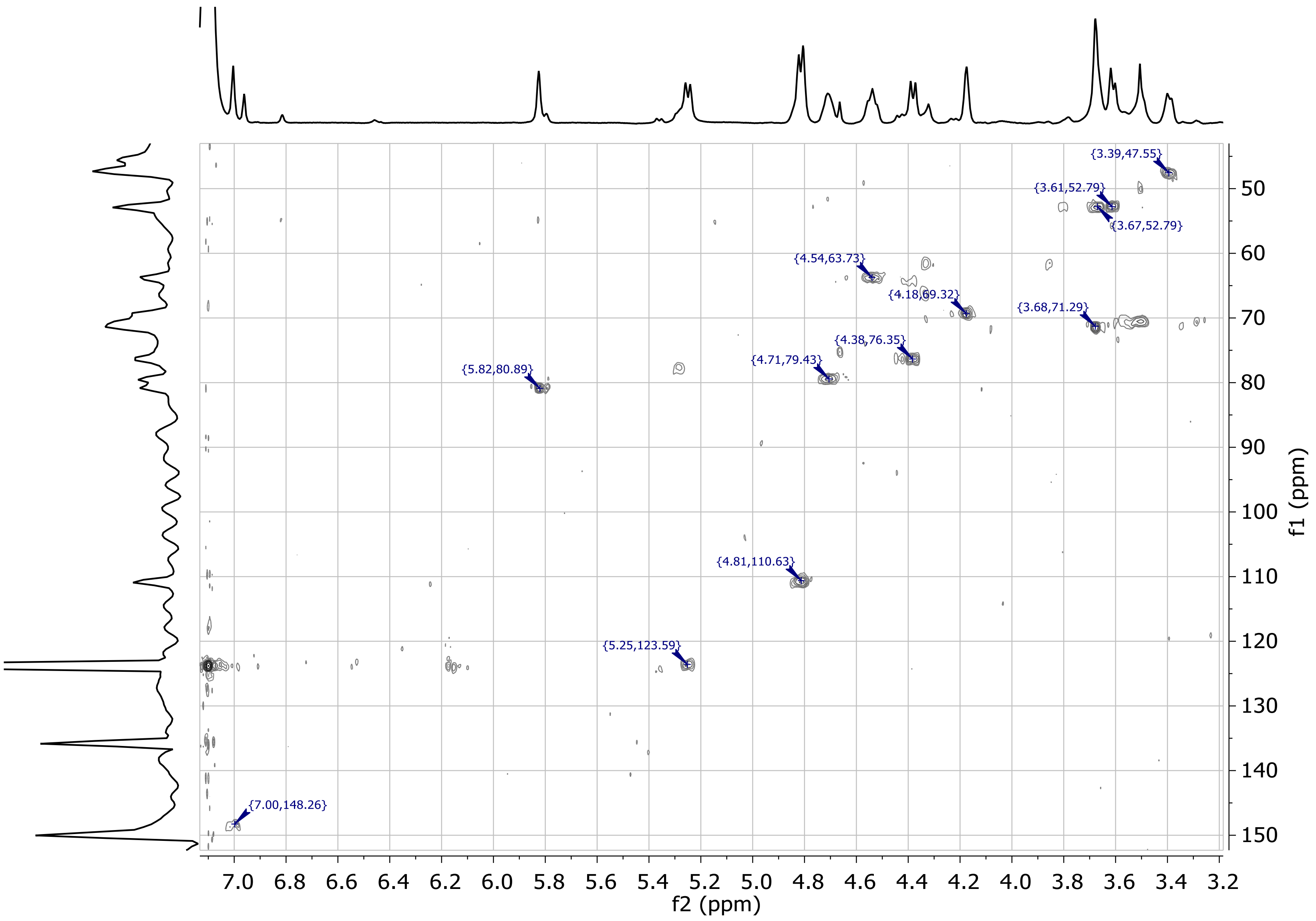

Figure S44: Slice 1 of 2D HSQC spectra of 20-Hydroxy-13,19-didesMethyl-SPX C (600 MHz Proton frequency, pyridine-d5).

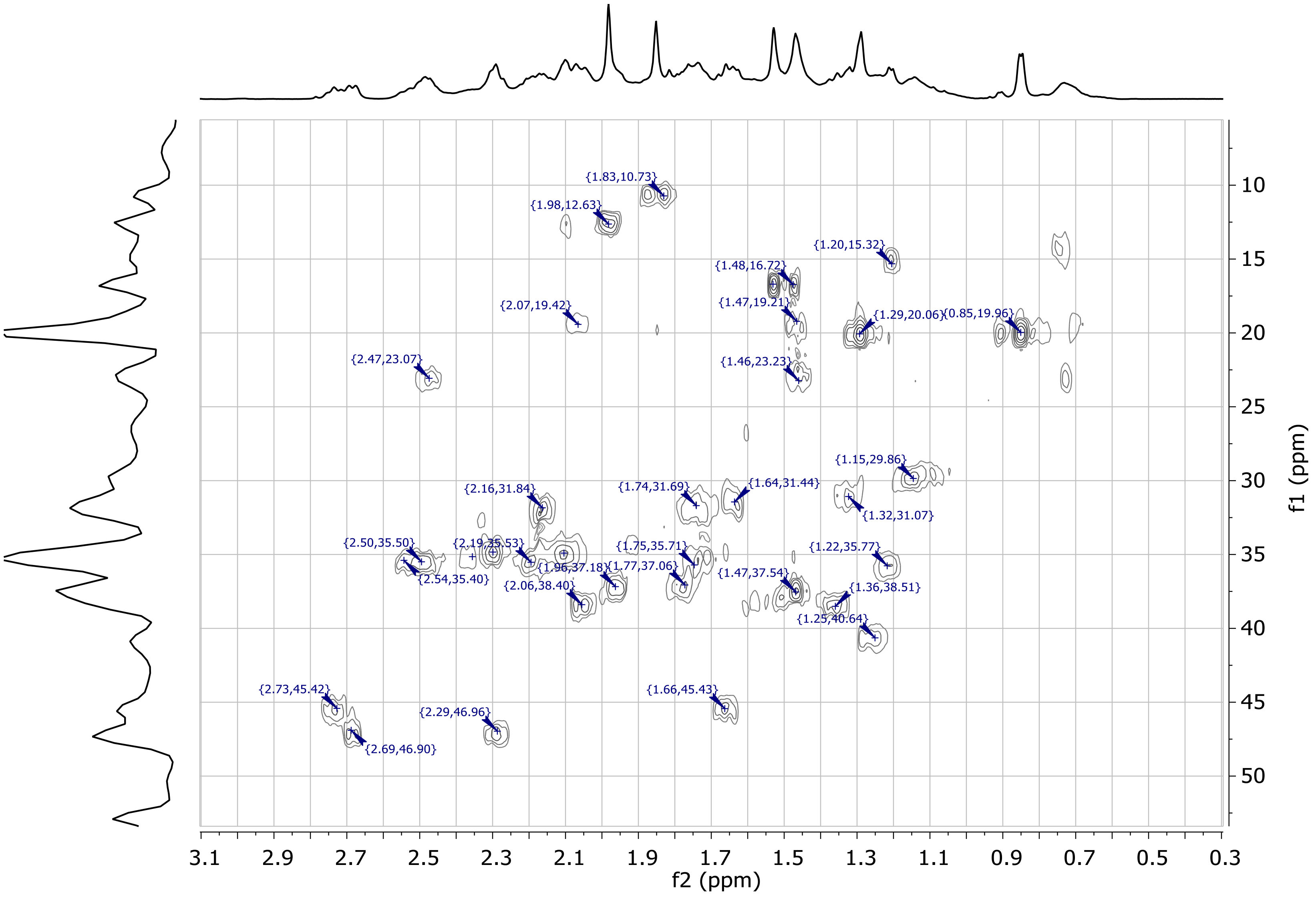

Figure S45: Slice 2 of 2D HSQC spectra of 20-Hydroxy-13,19-didesMethyl-SPX C (600 MHz Proton frequency, pyridine-d_5).

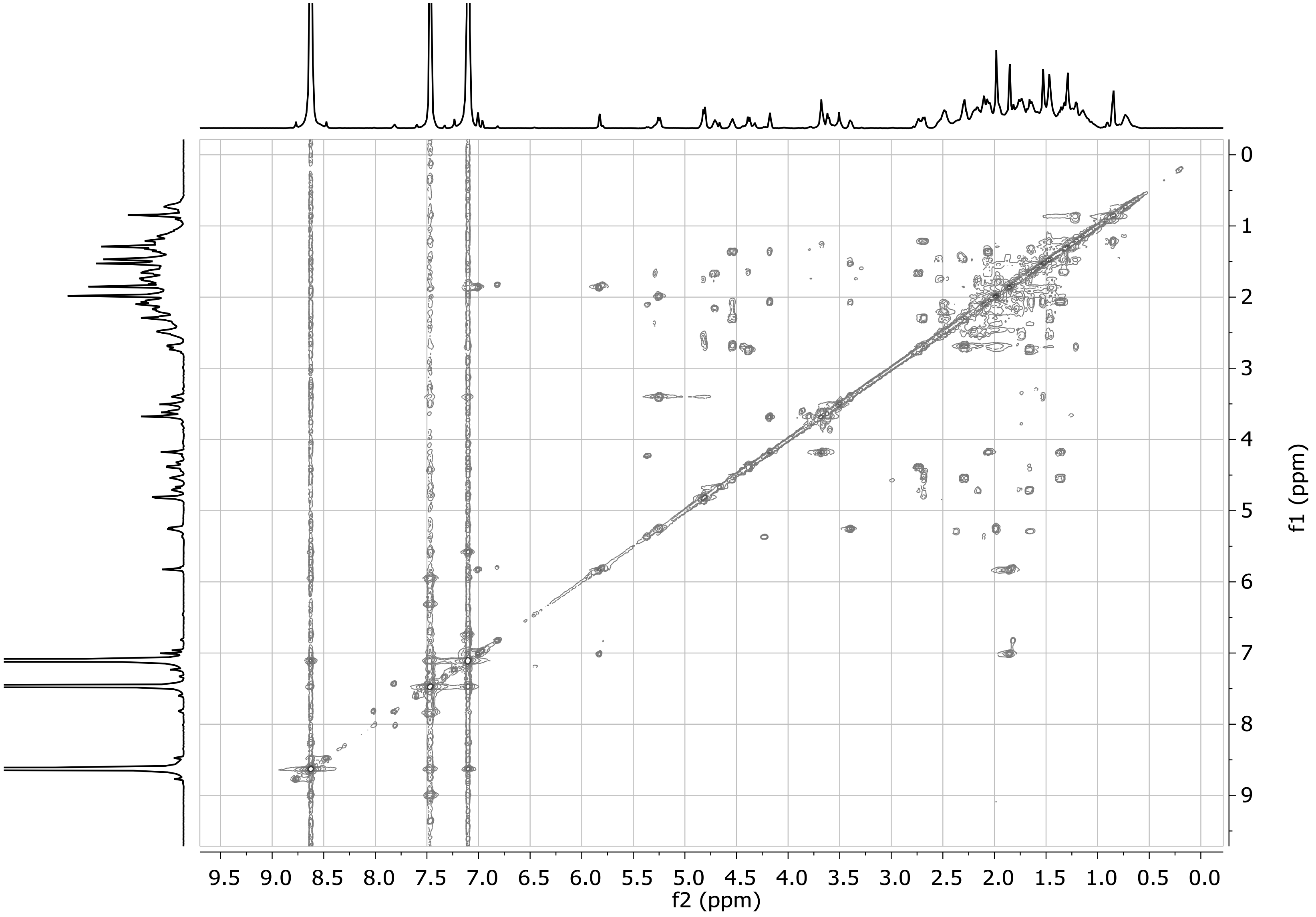

Figure S46: 2D COSY spectra of 20-Hydroxy-13,19-didesMethyl-SPX C (600 MHz, pyridine-d_5).

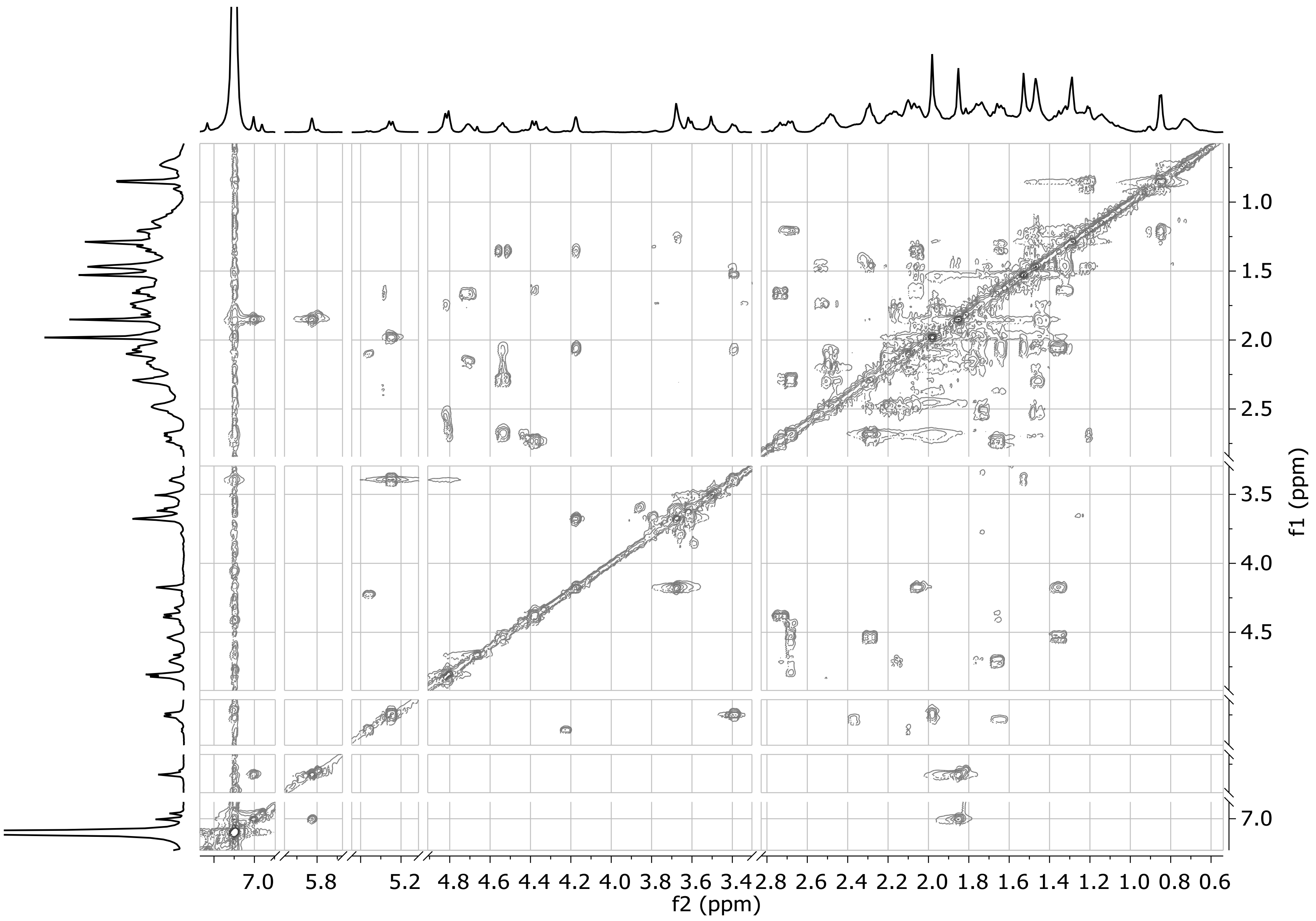

Figure S47: Slice of 2D COSY spectra of 20-Hydroxy-13,19-didesMethyl-SPX C (600 MHz, pyridine-d_5).

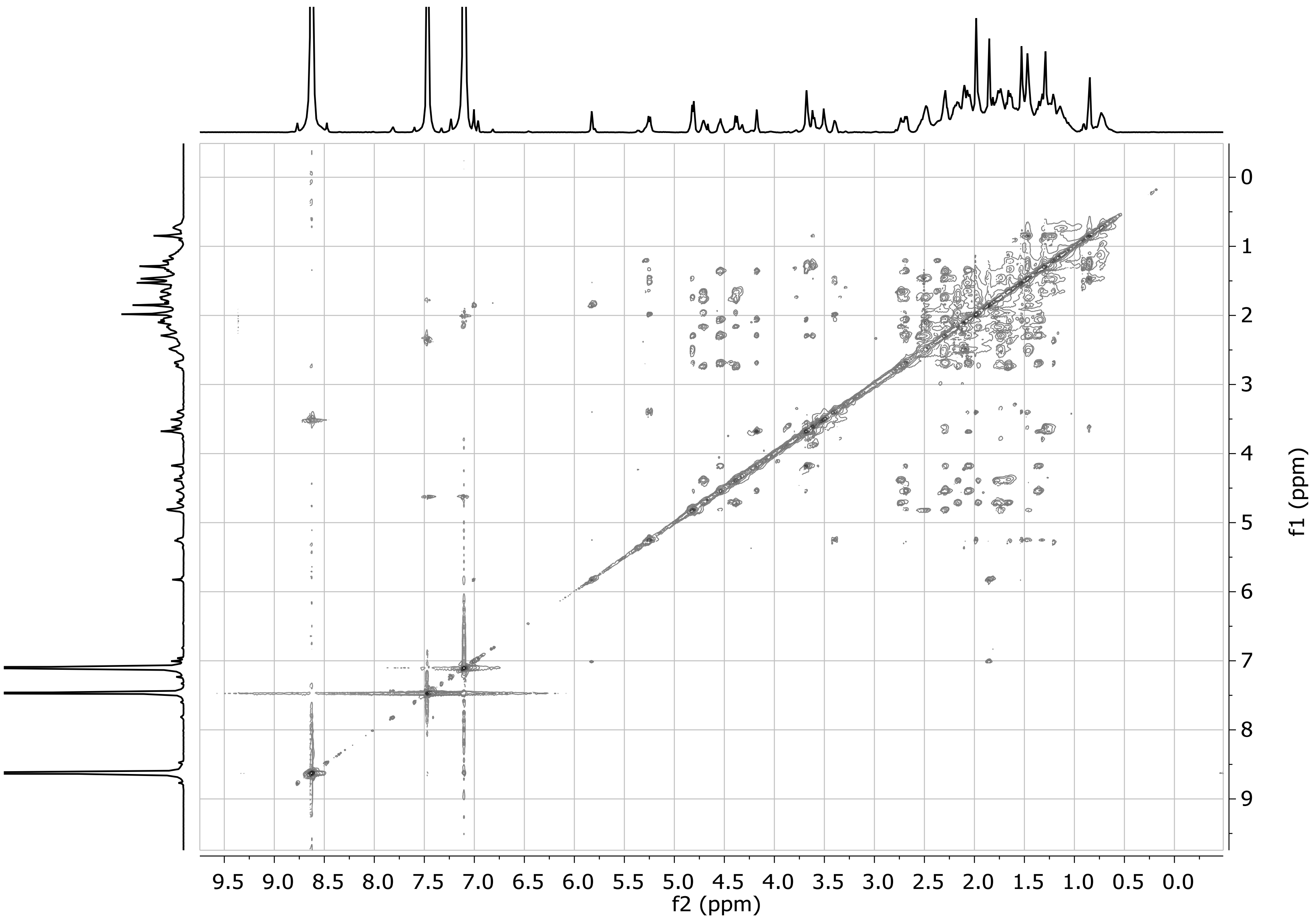

Figure S48: 2D TOCSY spectra of 20-Hydroxy-13,19-didesMethyl-SPX C (600 MHz, pyridine-d_5).

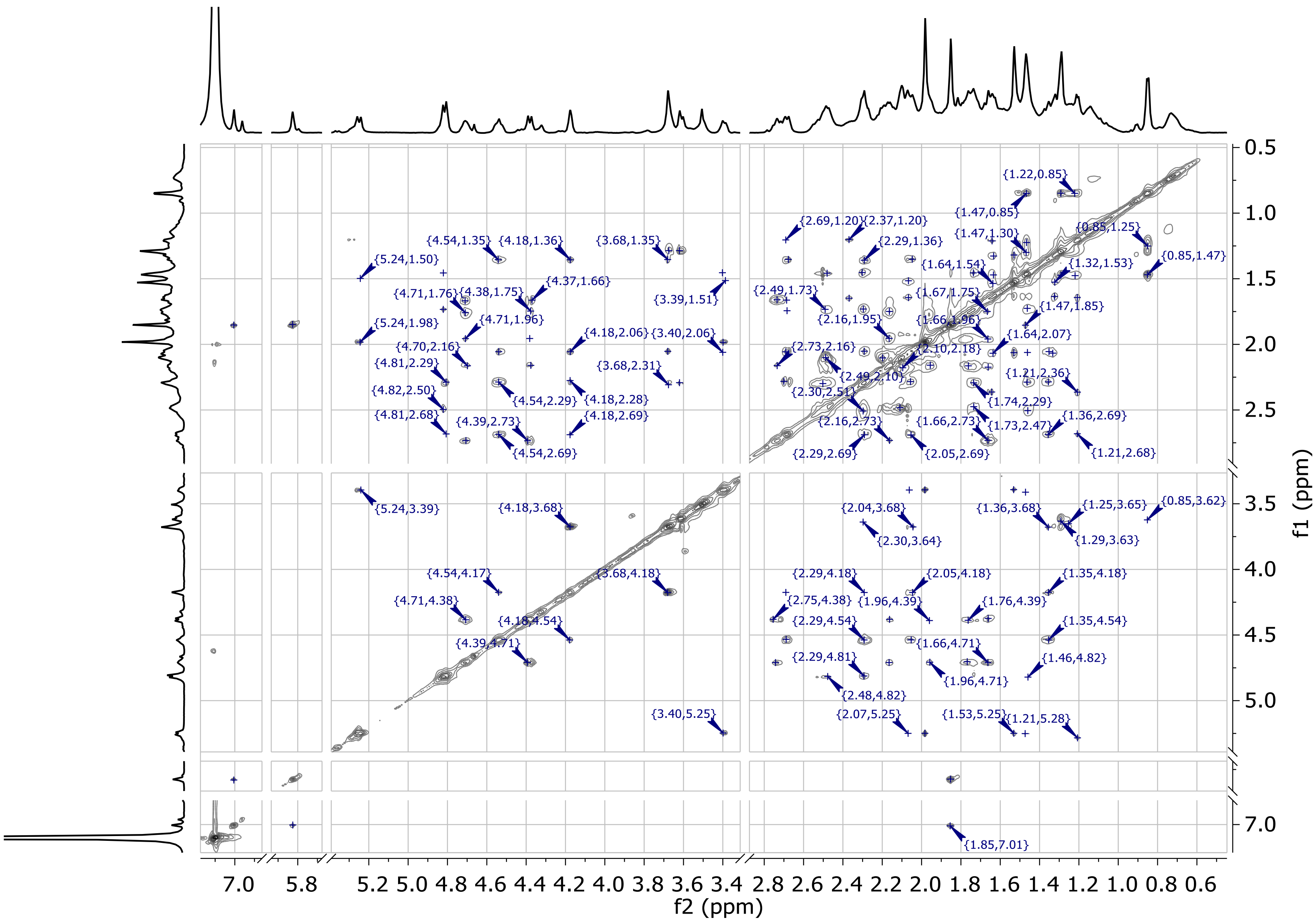

Figure S49: Slice of 2D TOCSY spectra of 20-Hydroxy-13,19-didesMethyl-SPX C (600 MHz, pyridine-d$_5$).

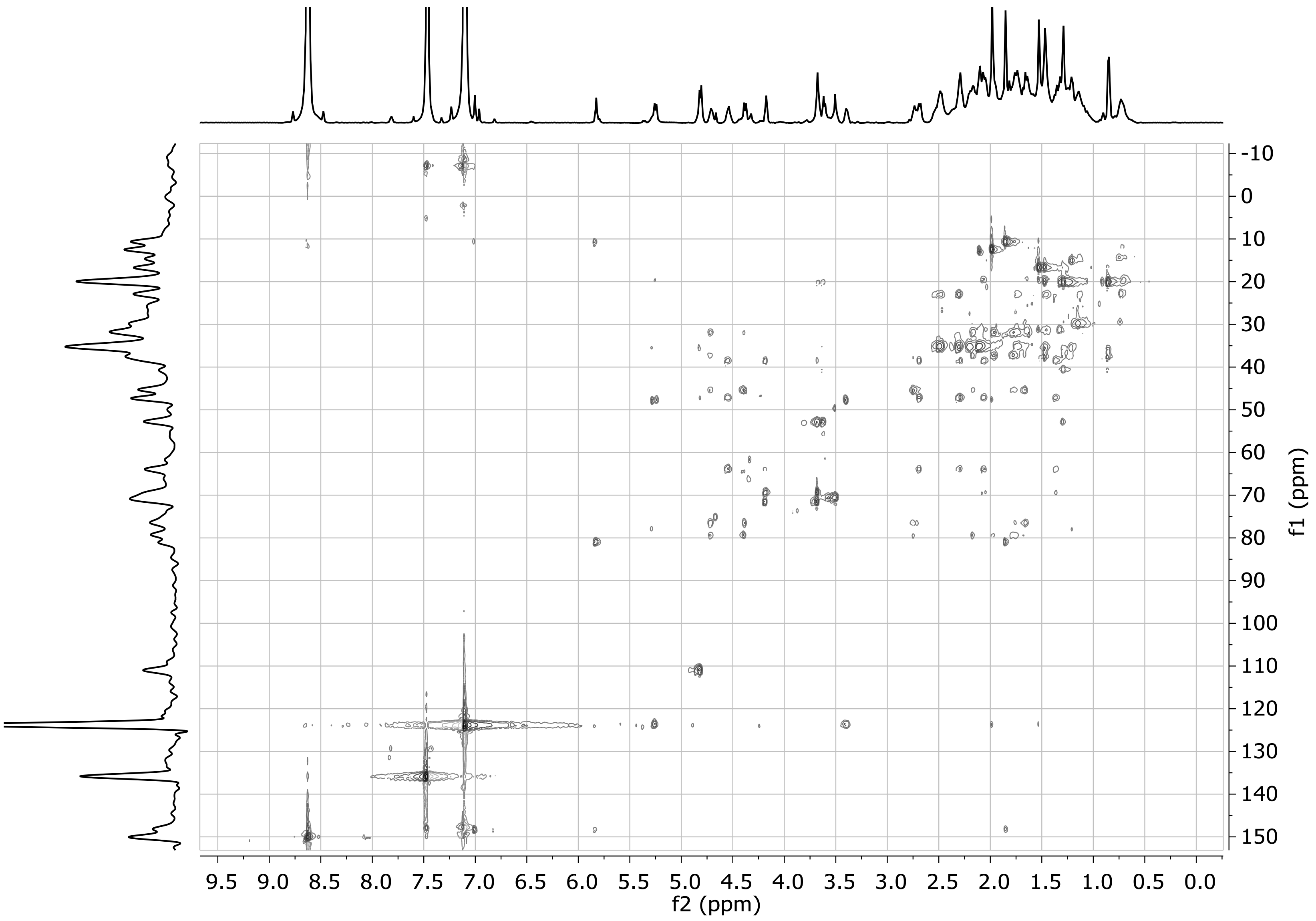

Figure S50: 2D HSQC-TOCSY spectra of 20-Hydroxy-13,19-didesMethyl-SPX C (600 MHz Proton frequency, pyridine-d$_5$).

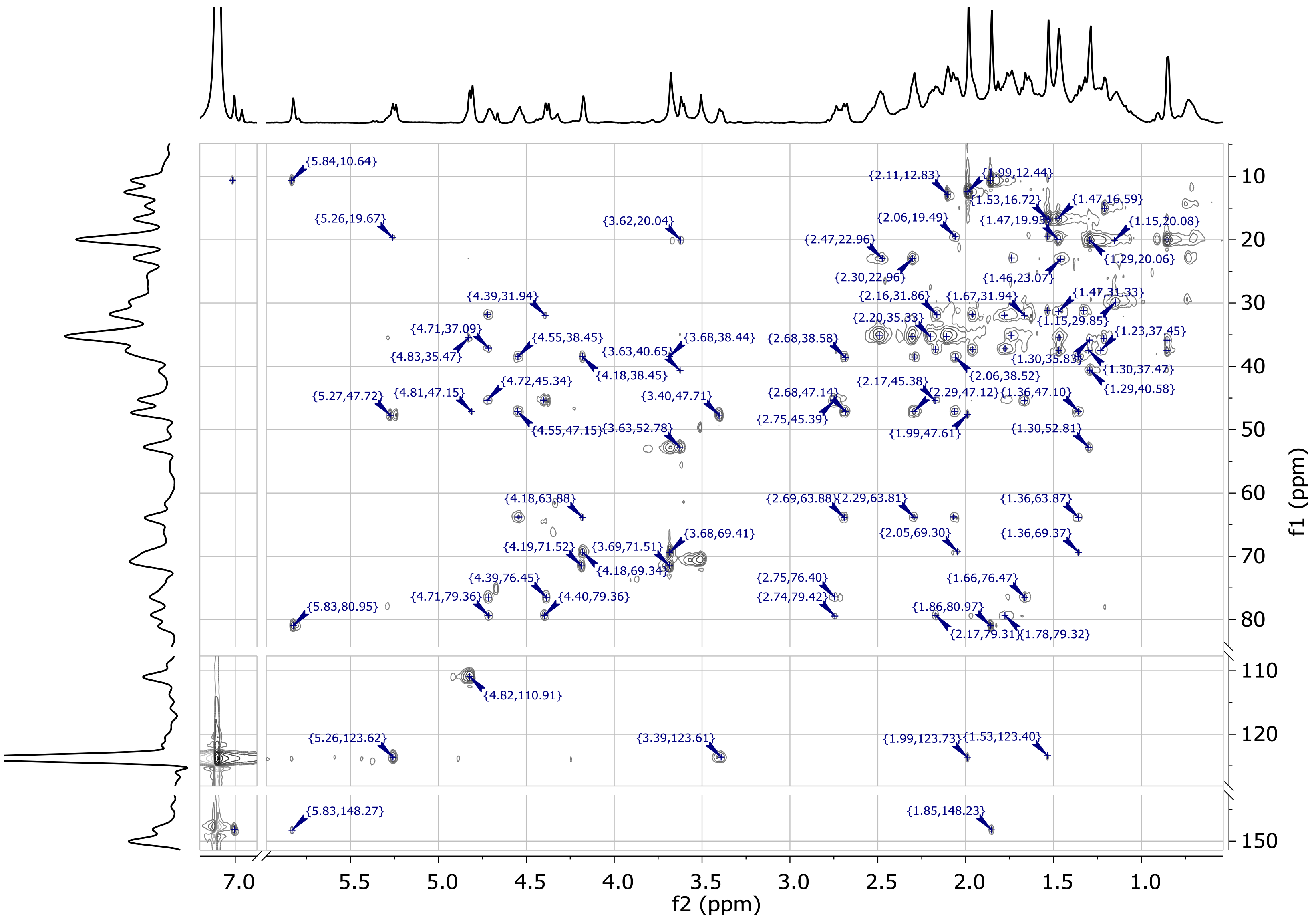

Figure S51: Slice of 2D HSQC-TOCSY spectra of 20-Hydroxy-13,19-didesMethyl-SPX C (600 MHz Proton frequency, pyridine-d5).

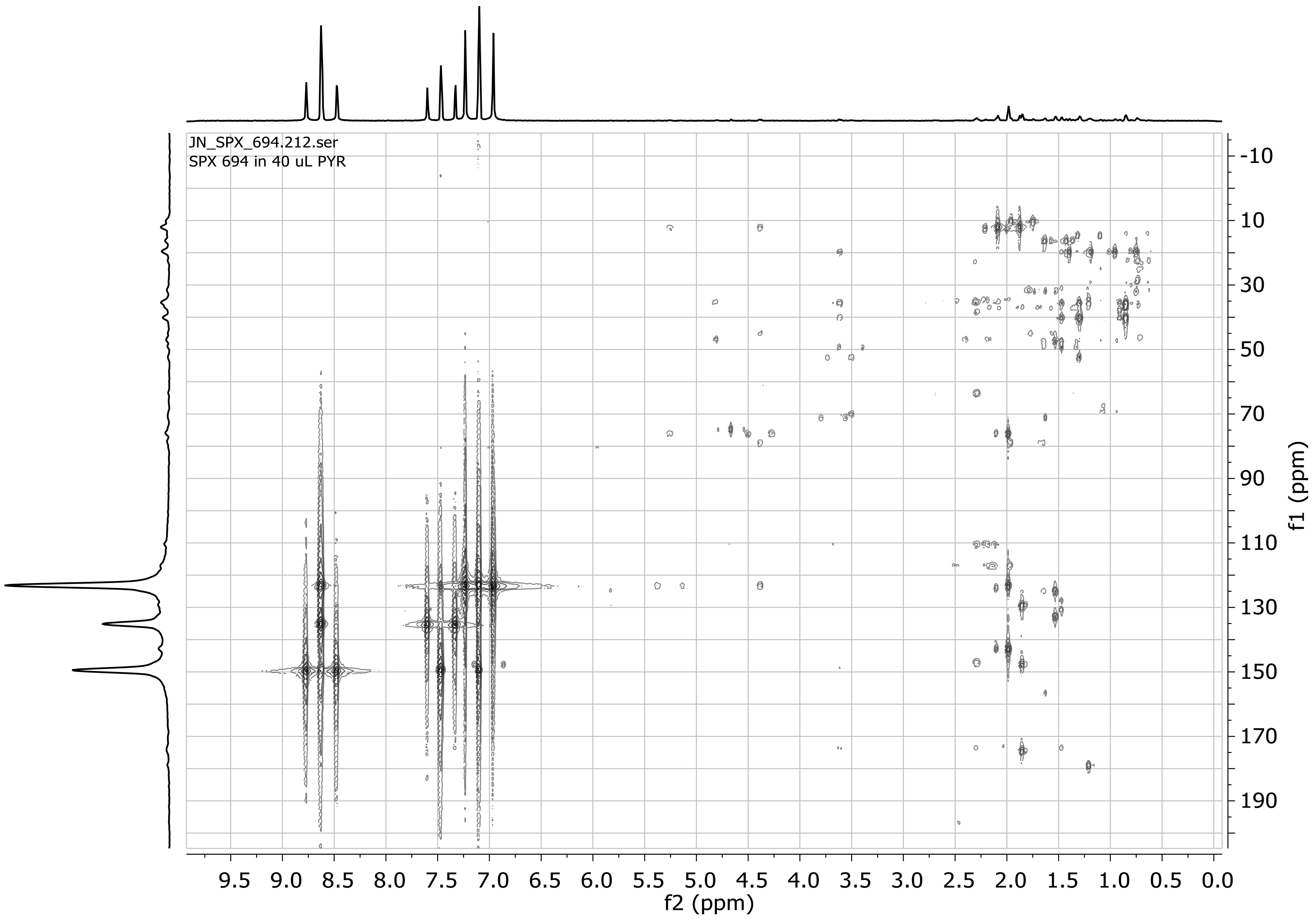

Figure S52: 2D HMBC spectra of 20-Hydroxy-13,19-didesMethyl-SPX C (600 MHz Proton frequency, pyridine-d$_5$).

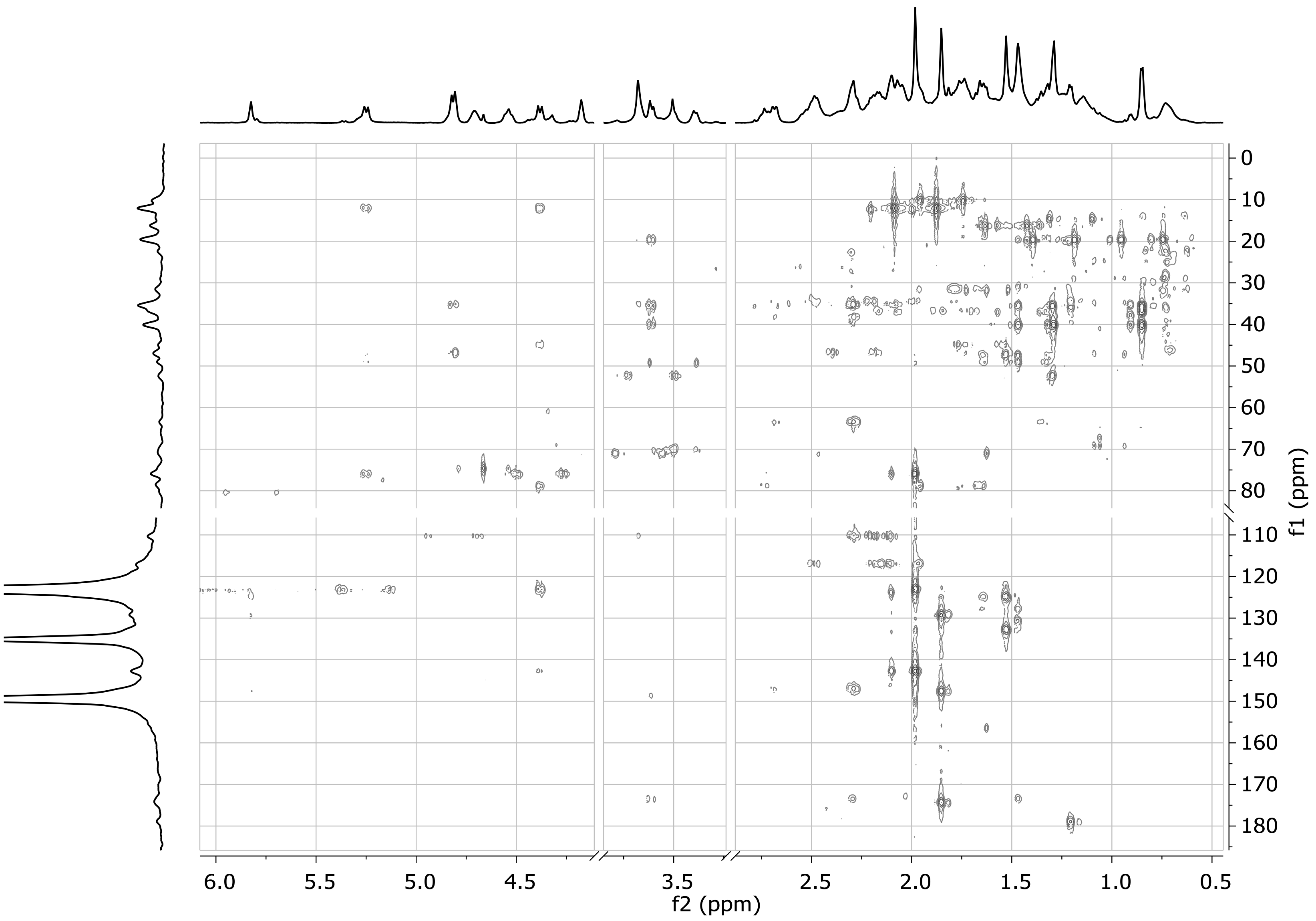

Figure S53: Slice of 2D HMBC spectra of 20-Hydroxy-13,19-didesMethyl-SPX C (600 MHz Proton frequency, pyridine-d$_5$).

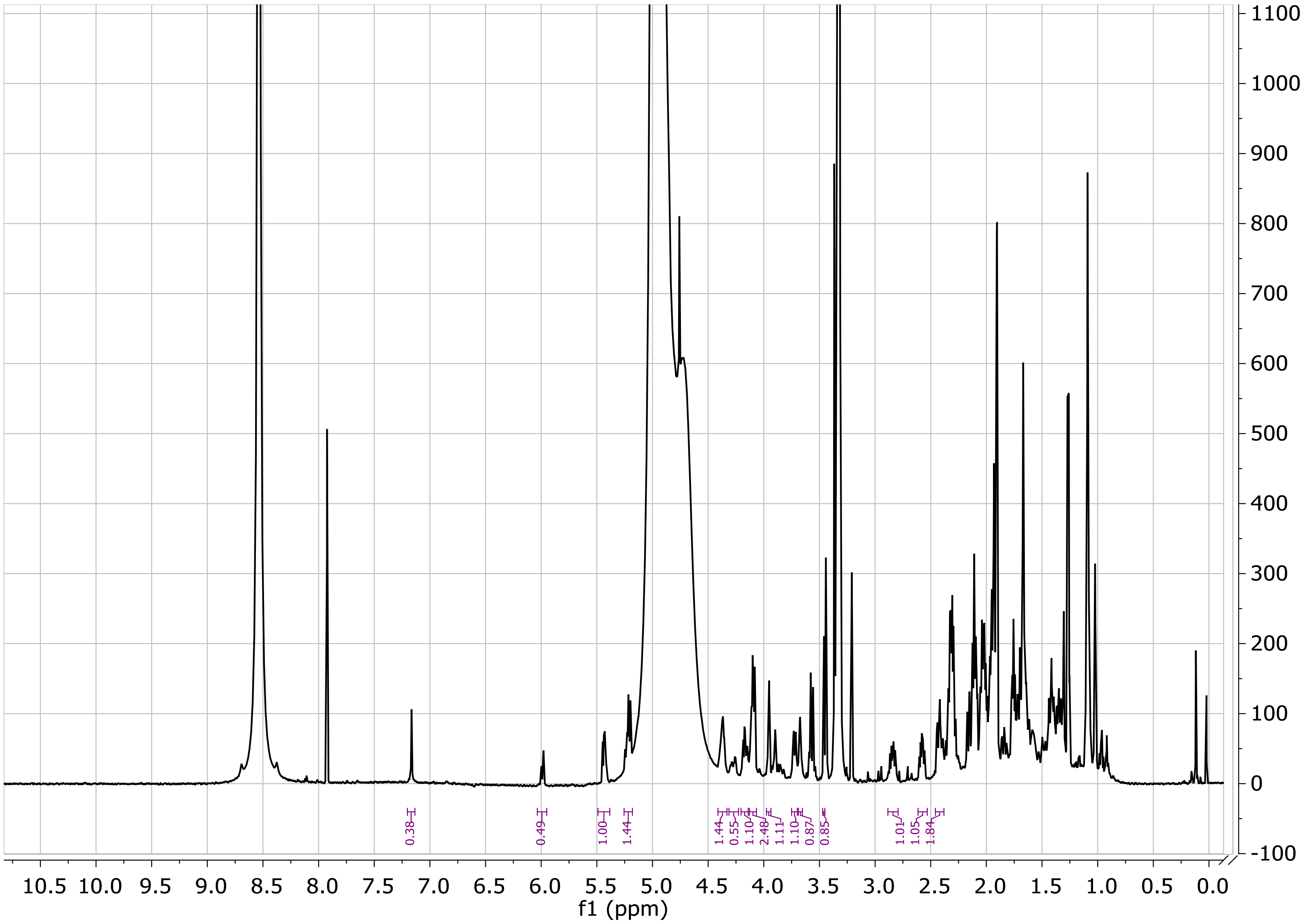

Figure S54: 1D Proton spectra of 20-Hydroxy-13,19-didesMethyl-SPX D (600 MHz, CD_3OD).

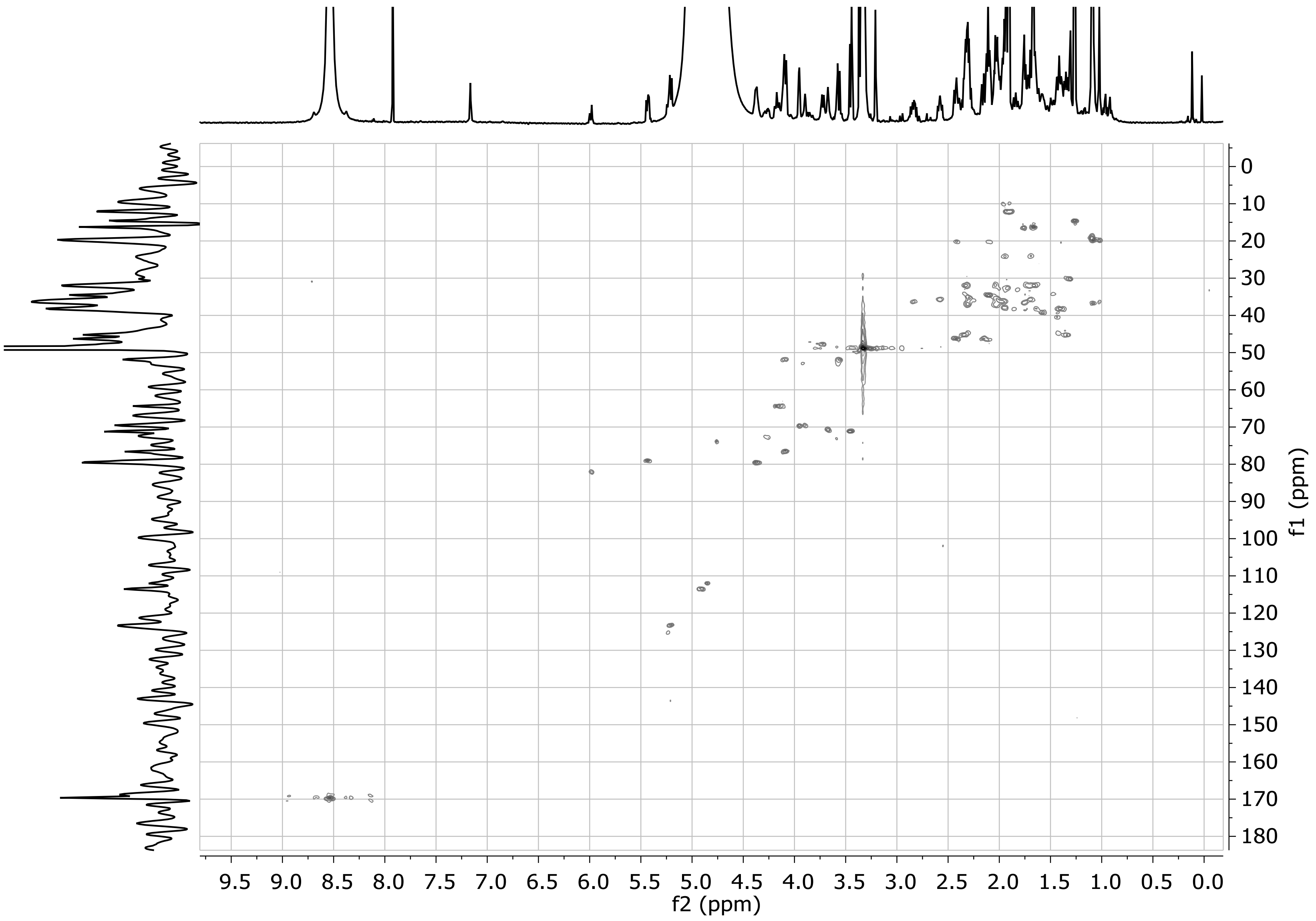

Figure S55: 2D HSQC spectra of 20-Hydroxy-13,19-didesMethyl-SPX D (600 MHz Proton frequency, CD_3OD).

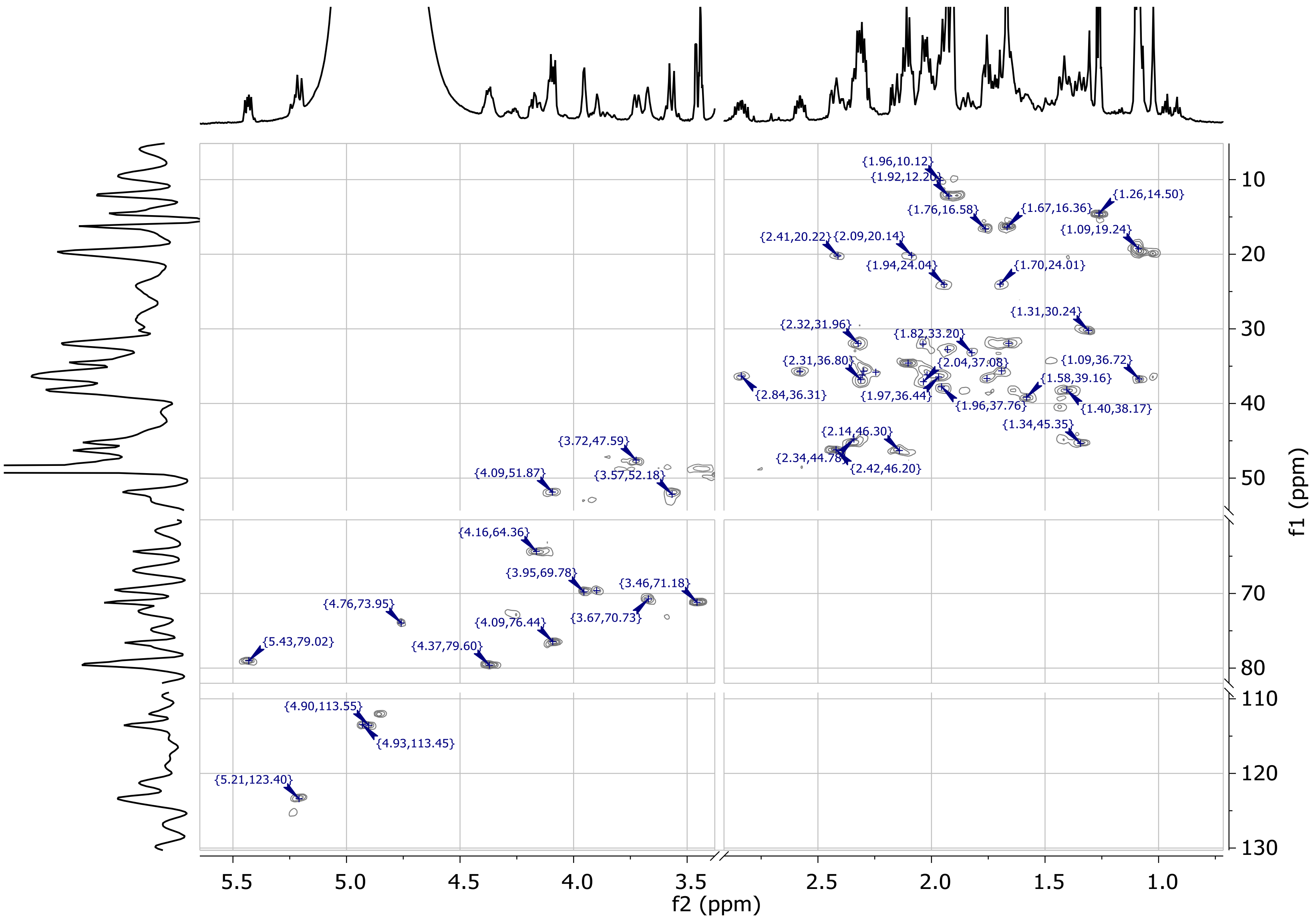

Figure S56: Slice of 2D HSQC spectra of 20-Hydroxy-13,19-didesMethyl-SPX D (600 MHz Proton frequency, CD_3OD).

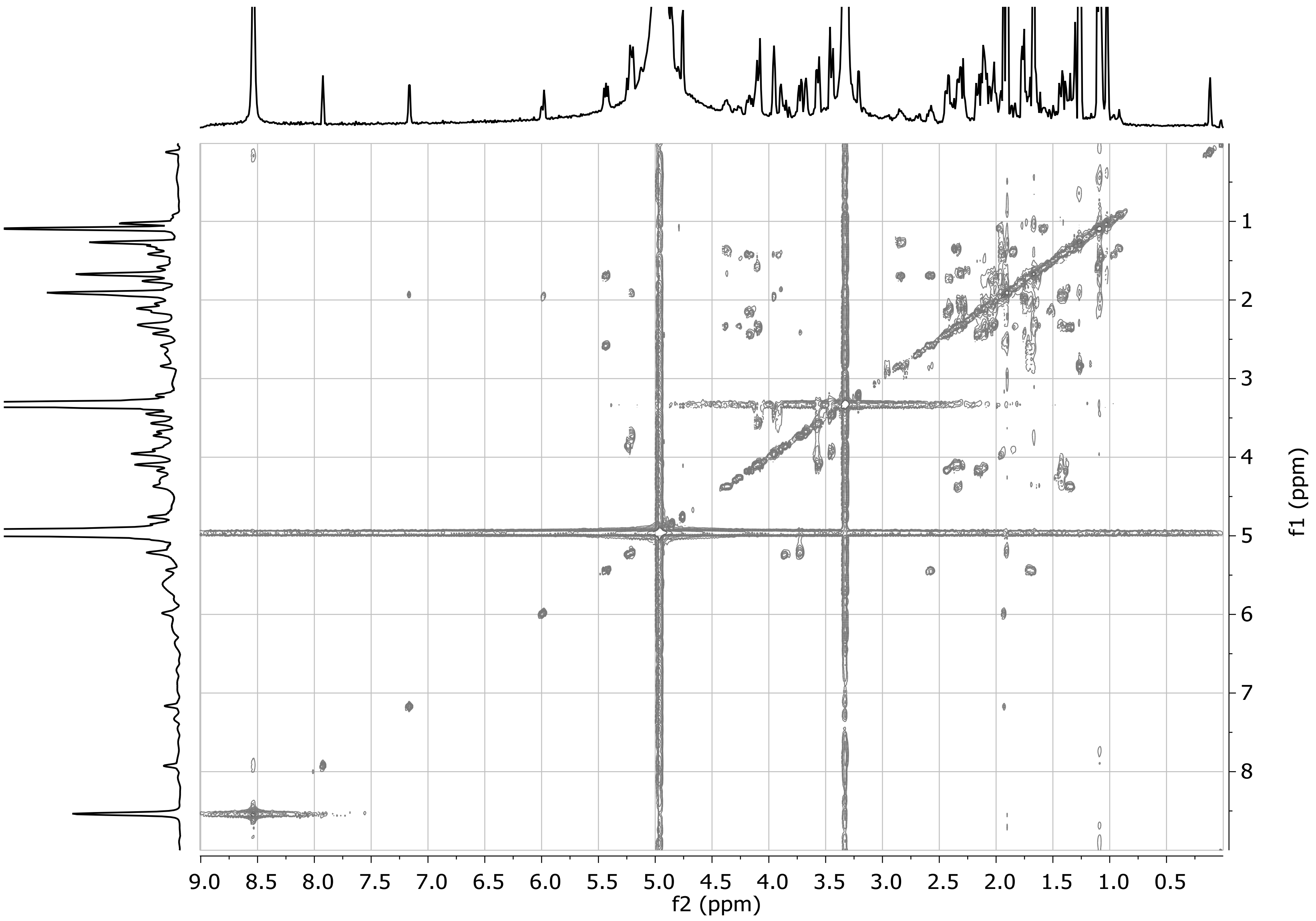

Figure S57: 2D COSY spectra of 20-Hydroxy-13,19-didesMethyl-SPX D (600 MHz, CD_3OD).

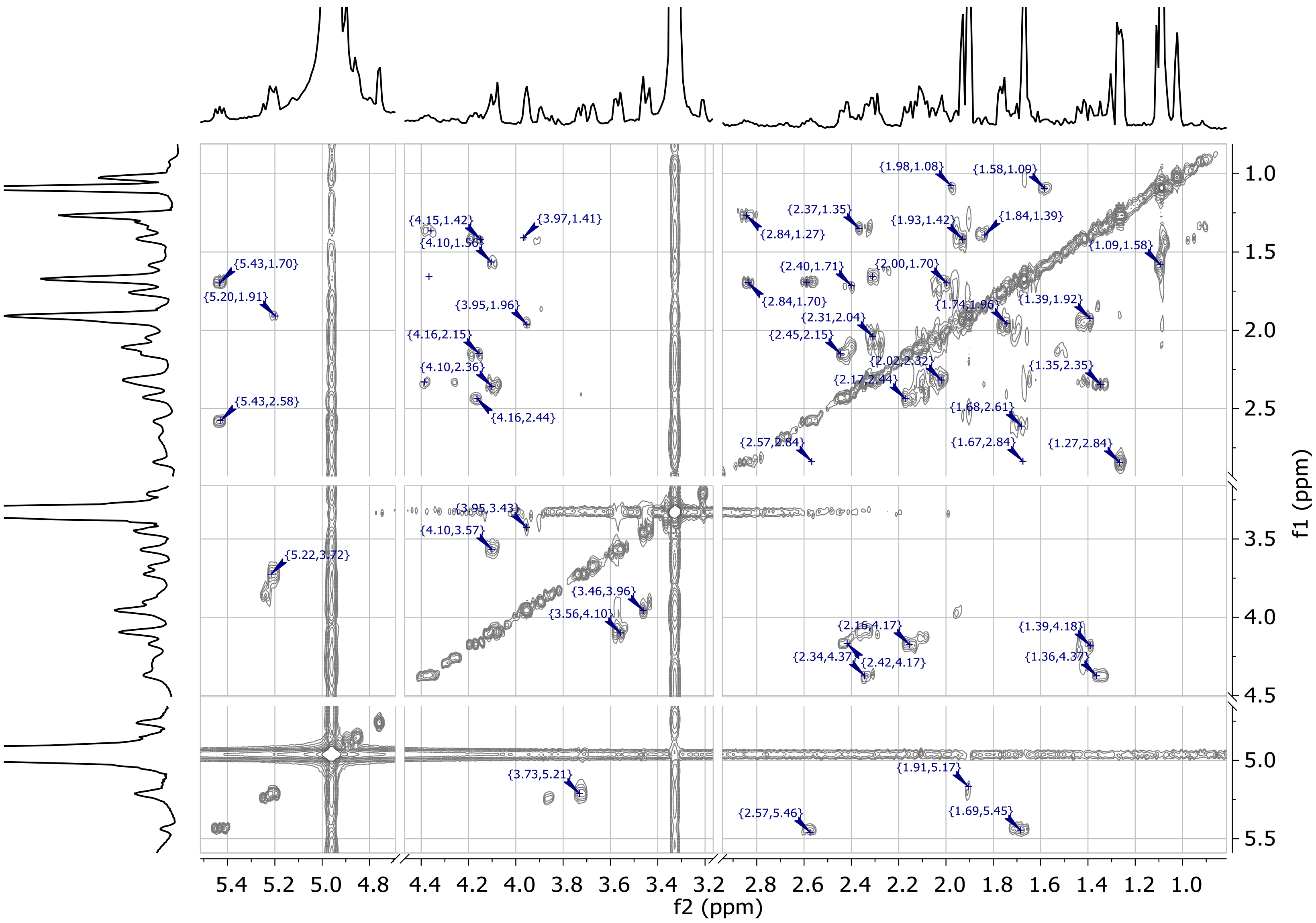

Figure S58: Slice of 2D COSY spectra of 20-Hydroxy-13,19-didesMethyl-SPX D (600 MHz, CD_3OD).

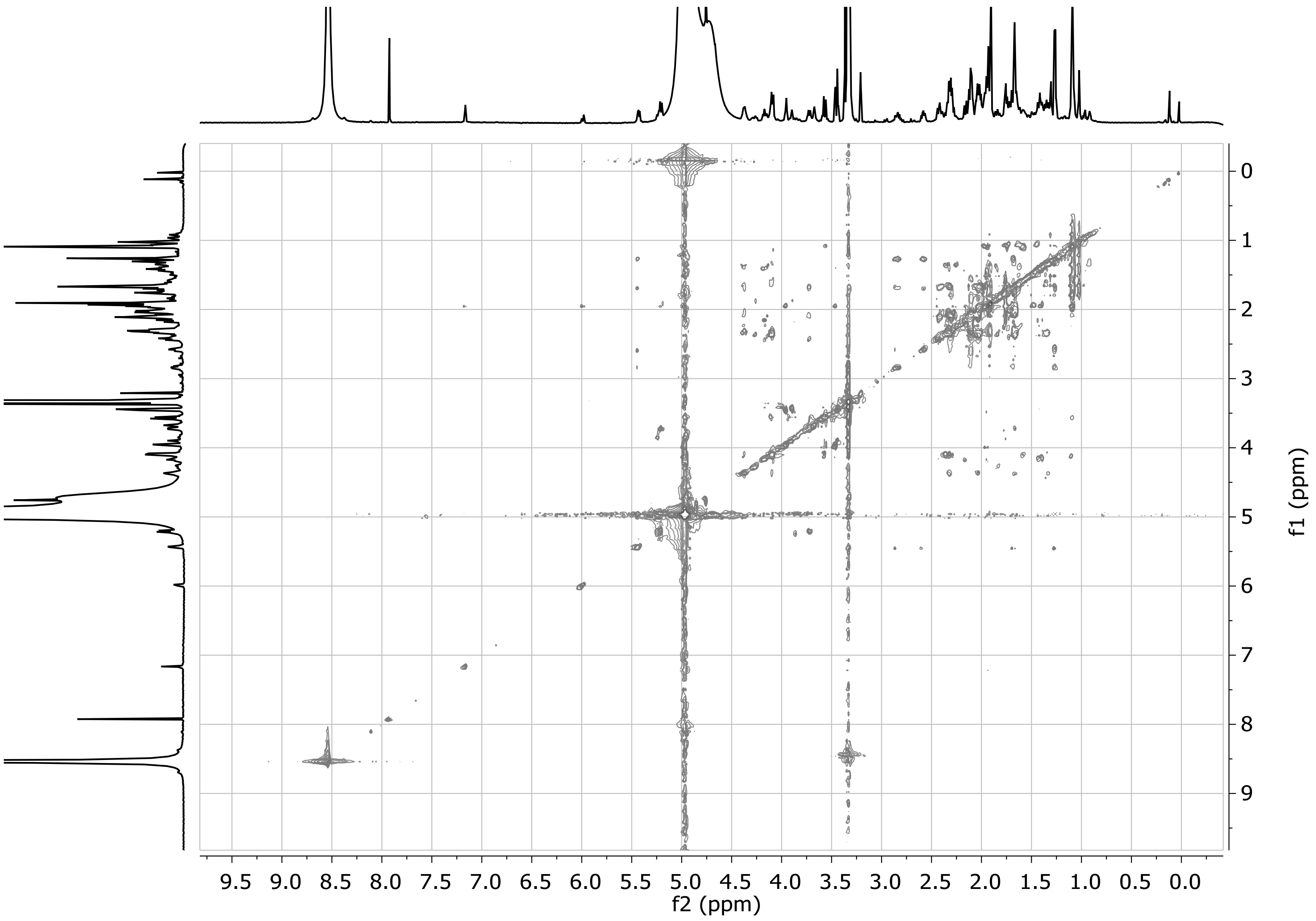

Figure S59: 2D TOCSY spectra of 20-Hydroxy-13,19-didesMethyl-SPX D (600 MHz, CD_3OD).

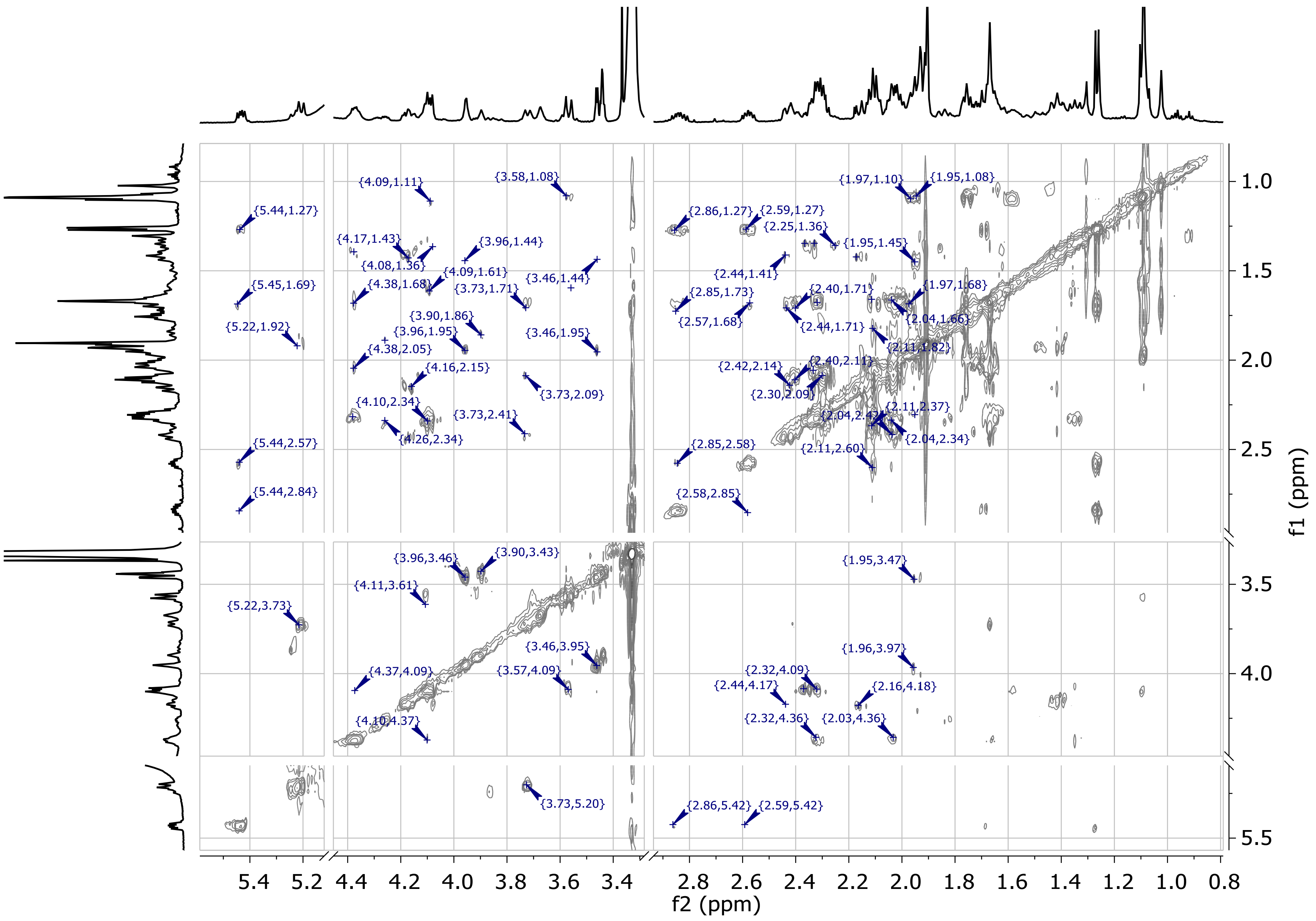

Figure S60: Slice of 2D TOCSY spectra of 20-Hydroxy-13,19-didesMethyl-SPX D (600 MHz, CD_3OD).

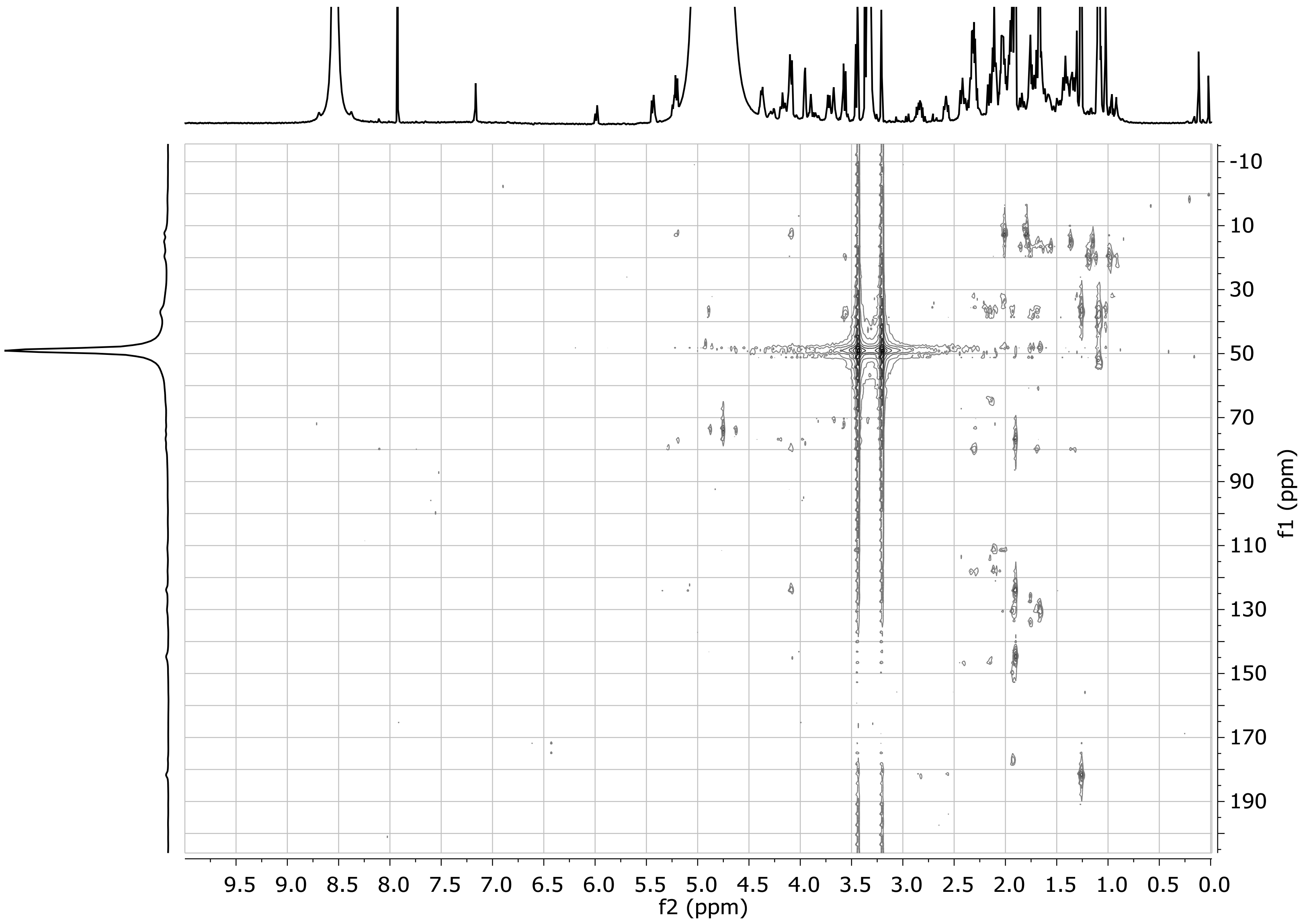

Figure S61: 2D HMBC spectra of 20-Hydroxy-13,19-didesMethyl-SPX D (600 MHz Proton frequency, CD_3OD).

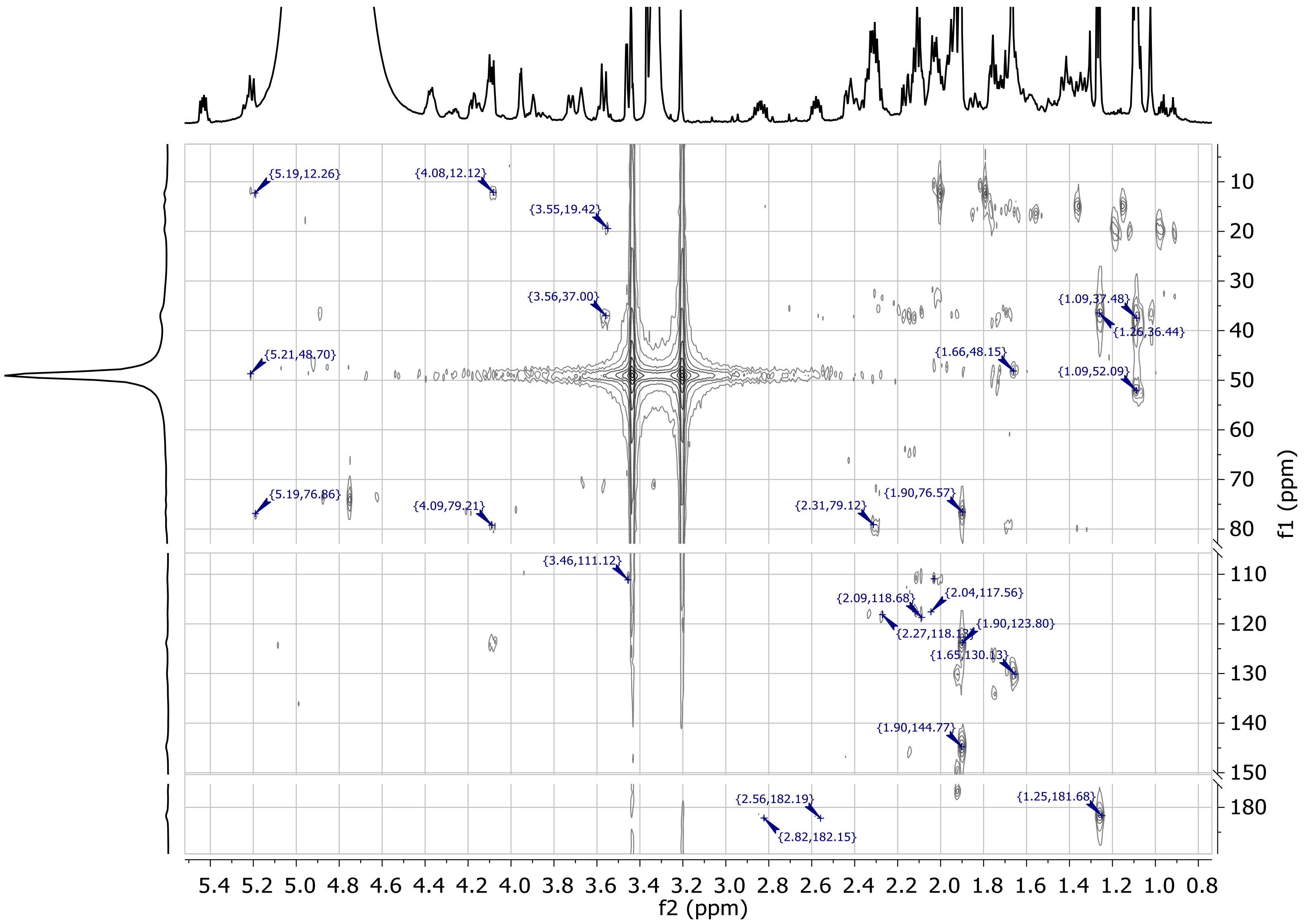

Figure S62: Slice of 2D HMBC spectra of 20-Hydroxy-13,19-didesMethyl-SPX D (600 MHz Proton frequency, CD_3OD).

Table S1: Mass transitions of spiroimines included in LC-MS/MS analysis (*m*/*z*; precursor ion → fragment ion), retention times and characteristic group fragments. Compounds detected in *A. ostenfeldii* strain OKNL48 originally isolated from Ouwerkerkse Kreek, the Netherlands, and their respective data are underlined.

**Mass Transition (*m*/*z*)**	**Common Name**	**Reference**	**Retention Time (min)**	***m*/*z***			
				**Group 1**	**Group 2**	**Group 3**	**Group 4**
			**Spirolides**				
650 → 164	Spirolide H	[18]		650/632/614	402/384	206	164
652 → 164	Spirolide I	[18]		652/634/616	402/384	206	164
678 → 164	13,19-didesMethyl-spirolide C	[19,20]		678/660/642/624	448/430/412/394		164
692 → 150	Spirolide A	[13]		692/674/624	444/390	190	150
692 → 164	13-desMethyl-spirolide C	[6,13]	12.76	692/674/656/638	462/444/426		164
692 → 164	Spirolide G	[21]		692/674/656/638	378		164
694 → 150	Spirolide B	[22,23]		694/676/658/640	462/444/426		150
694 → 164	13-desMethyl spirolide D	[24]		694/676/658/640	444/426	230/204/177	164
694 → 164	(10)	[6]	12.33	694/676/658/640/622	446/428/410	292/274/248	164
696 → 164	(11)	[6]	12.39	696/678/660/642/624	464/446/428/410	292/274/248	164
706 → 164	Spirolide C	[13]		706/688/638	458/404	204	164
706 → 164	20-Methyl-spirolide G	[25]		706/688/670/652	392/374/346	258	164
708 → 164	Spirolide D	[22,25]		708/690/672/654	458/440	230/206/204/177	164
708 → 180	27-Hydroxy-13-desMethyl-spirolide C	[6]	13.06	708/690/672/654/636	478/460/442/424		180
710 → 164	(12)	[6]	12.97	710/692/674/656/638	462/444/426		164
720 → 164	(13)	[6]	13.29	720/702/684/666	490/472/454		164
722 → 164	(14)	[6]	13.40	722/704/686/668	490/472/454		164
			Gymnodimines				
508 → 490	Gymnodimine A	[6,12]	11.81	508/490/392/286/246/202/174/162/136/121			
510 → 492	(1)	[6]	12.21	510/492/482/332/302/136/120			
522 → 504	12-Methyl-gymnodimine A	[5,6]	12.17	522/504/406/300/246/202/174/162/136/120			
526 → 508	(2)	[6]	11.32	526/508/348/262/174/162/136/120			

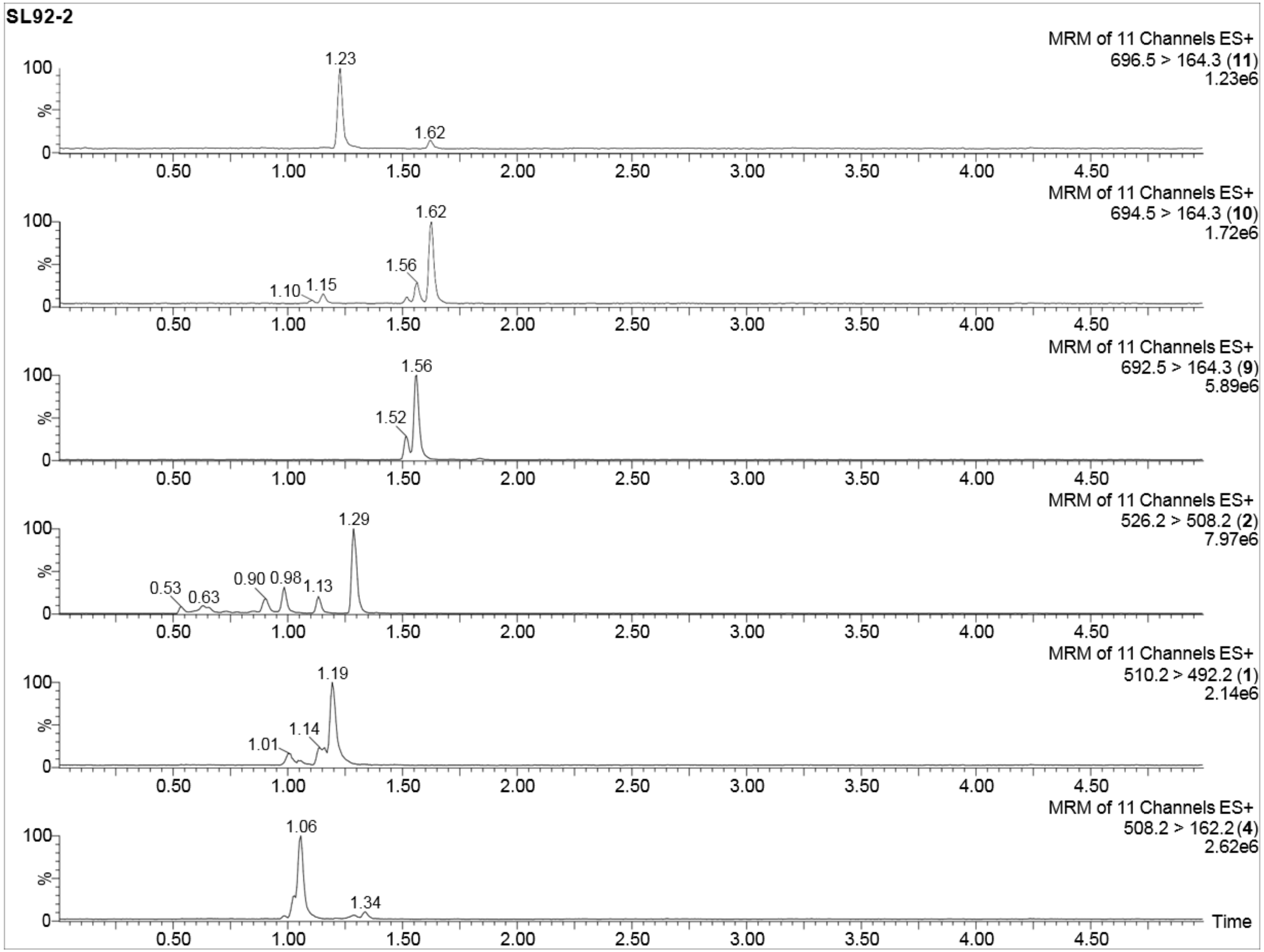

Figure S63: LC-MS/MS chromatogram of station SL92-2.
